# Longitudinal lipoprotein and inflammatory mediators analysis uncover persisting inflammation and hyperlipidemia following SARS-CoV-2 infection in long COVID-19

**DOI:** 10.1007/s11306-025-02262-y

**Published:** 2025-05-07

**Authors:** Gyuntae Bae, Zhiqi Yang, Daniele Bucci, Claire Wegner, Hartmut Schäfer, Yogesh Singh, Caterina Lonati, Christoph Trautwein

**Affiliations:** 1 Werner Siemens Imaging Center, Department of Preclinical Imaging and Radiopharmacy, University Hospital Tübingen, Tübingen, Germany; 2 Cluster of Excellence iFIT (EXC2180) ’Image-Guided and Functionally Instructed Tumor Therapies’, University of Tübingen, Tübingen, Germany; 3 Research Institute of Women’s Health, University of Tübingen, Tübingen, Germany; 4 Bruker BioSpin GmbH & Co. KG, Biopharma & Applied Division, Ettlingen, Germany; 5 Institute of Medical Genetics and Applied Genomics, University of Tübingen, Tübingen, Germany; 6 Next Generation Sequencing (NGS) Competence Center Tübingen (NCCT), University of Tübingen, Tübingen, Germany; 7 Center for Preclinical Research, Fondazione IRCCS Ca’ Granda Ospedale Maggiore Policlinico, Milan, Italy; 8 M3 Research Center for Malignome, Metabolome and Microbiome, Faculty of Medicine, University of Tübingen, Tübingen, Germany; 9 Core Facility Metabolomics, Faculty of Medicine, University of Tübingen, Tübingen, Germany

**Keywords:** Apolipoproteins, lipoproteins/Metabolism, Dyslipidemia, Lipoproteomics, Inflammatory mediators, Anti-inflammatory mediators

## Abstract

**Introduction:**

Individuals suffering from acute COVID-19 (AC) often develop long COVID-19 (LC) syndrome that is associated with aberrant levels of lipoproteins and inflammatory mediators. Yet, these dysregulations are heterogenous due to the uncertain prevalence and require a more extensive characterization.

**Objectives:**

This study aimed to investigate LC-associated dysregulations in inflammatory mediators and lipids by longitudinal Nuclear Magnetic Resonance (NMR) lipoprotein analysis and cytokine profiling in human blood.

**Methods:**

We quantitatively profiled lipoproteins and inflammatory parameters in LC patients at 5 (n = 95), 9 (n = 73), 12 (n = 95), 16 (n = 78), and 20 (n = 85) months post AC by in vitro diagnostics research (IVDr)-based NMR spectroscopy. Simultaneously, we assessed inflammatory meditators with a 13-plex cytokine panel by flow cytometry. We then compared the lipoprotein profiles with historical data from AC (*N* = 307) and healthy cohorts collected before the COVID-19 pandemic (*N* = 305), whereas the cytokine profiles were correlated with that of the AC cohort.

**Results:**

We identified 31 main and 80 significantly altered subclass lipoproteins, respectively. LC was associated with higher serum levels of very low-density, intermediate-density, low-density, high-density lipoproteins, along with triglycerides, cholesterols, and apolipoprotein a-I & a-II lipoproteins compared to the healthy cohort. We also observed significantly lower concentrations of NMR-based inflammatory parameters in LC than in AC cohort, whilst proinflammatory mediators IFN-α2, IFN-γ, TNF-α, CXCL8/IL-8, IL-12p70, IL-17 A, and IL-23 displayed significantly higher concentrations in LC compared with the AC cohort. Conversely, CCL2/MCP-1, IL-6, and IL-18 were significantly higher in the AC cohort than in LC.

**Conclusion:**

Our findings demonstrate a persistent hyperlipidemic phenotype in LC alongside signs of chronic inflammation and lipoprotein metabolism that vary in states of acute and chronic inflammation.

**Graphical abstract:**

Long COVID-19 is characterized by persistent hyperlipidemic and progressive chronic inflammatory phenotypes for up to 2 years after the acute infection

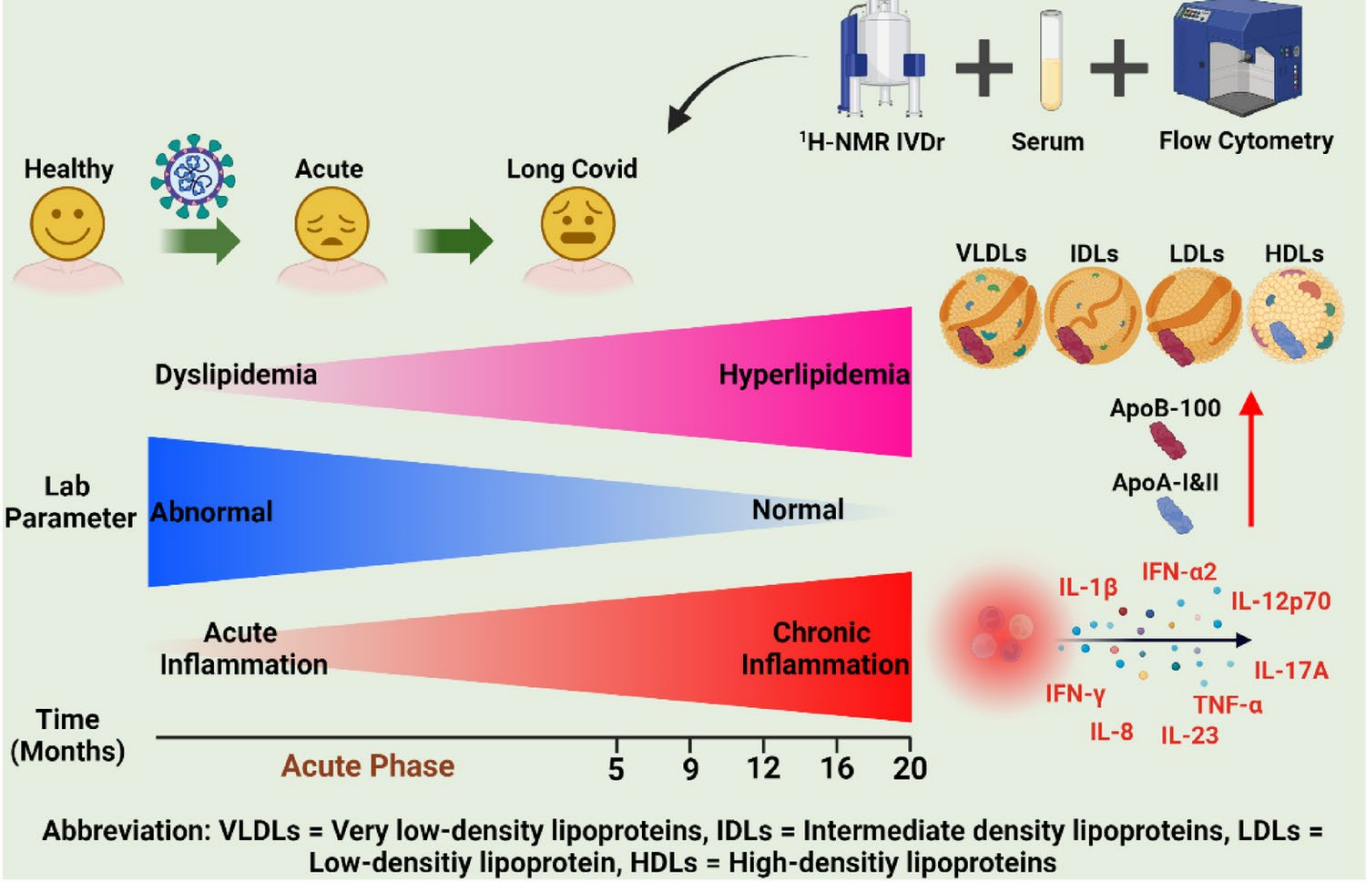

**Supplementary Information:**

The online version contains supplementary material available at 10.1007/s11306-025-02262-y.

Long COVID-19 is characterized by persistent hyperlipidemic and progressive chronic inflammatory phenotypes for up to 2 years after the acute infection.

## Introduction

The Coronavirus disease 2019 (COVID-19) arises from SARS-CoV-2 virus infection (Hu et al., 2021). This condition shows highly diverse severity, ranging from flu-like symptoms to pneumonia and multi-organ failure or dysfunction which have significantly impacted life expectancy worldwide (Huang et al., 2020; Schöley et al., 2022). Most of the individuals recover from acute COVID-19 (AC) within weeks depending on the symptoms and severities (Habtamu Tamiru et al., 2023). However, 10–20% of individuals are diagnosed as long COVID-19 or post-acute COVID-19 syndrome (LC) (Organization, 2022). It is defined as the development of persistent or new symptoms for more than 6 months to years after AC infection (Mizrahi et al., 2023; Munblit et al., 2022; Seeßle et al., 2022). The most frequently observed persistent symptoms are physical, cognitive, and sleep-related (Seeßle et al., 2022).

The pathogenesis of LC is not still fully elucidated. Yet, the underlying causes of the disease may involve various systemic alterations of which immune dysregulation and persistent viral reservoirs are the emerging postulates (Davis et al., 2023; Phetsouphanh et al., 2022; Proal & VanElzakker, 2021; Proal et al., 2023). The pathogenesis is related to age, sex, BMI, severities of AC infection, and status of hospitalization (inpatient or outpatient) (Tsampasian et al., 2023; Wu et al., 2024; Zhang et al., 2021). The huge heterogeneity of LC makes its diagnosis and prognosis challenging (Rando et al., 2021). Further investigation is sought-after at the systemic level with or without refined stratifications of the cohort. Herein, metabolomics, lipidomics, and lipoproteomics come out as powerful phenotyping tools, providing an in-depth physiological snapshot of such pathogenesis, together with the opportunity to trace inflammation (Berezhnoy et al., 2023; Kazenwadel et al., 2023; Lonati et al., 2024; López-Hernández et al., 2023b; Rössler et al., 2022). Our group has established a connection between the systemic alteration and clinical presentation of the AC cohort, which highlights the potential of metabolomics techniques to gain insight into the disease (Kazenwadel et al., 2023; Lonati et al., 2024; Rössler et al., 2022). Currently, the metabolic reprogramming may include impaired energy metabolism, dysregulated lipid metabolism, and dyslipidemia in LC (Berezhnoy et al., 2023; Guntur et al., 2022; López-Hernández et al., 2023a; Scherer et al., 2022).

To date, the definition of LC is still due to its clinical heterogeneity. Therefore, we aimed at investigating the lipidomic and inflammatory mediator profiles of longitudinal serum samples collected from LC patients at different time intervals after the initial acute infection. To this end, we exploited commercial in vitro diagnostics research (IVDr) nuclear magnetic resonance (NMR) standard operating procedures (SOPs) and flow cytometry-based cytokine assay. We examined the phenotypes of the cohort at 5 time points (5 months (M), 9 M, 12 M, 16 M, and 20 M) to point towards hallmarks of the disease in comparison to independent healthy and AC cohorts. Moreover, clinical lab parameters, including key hematological, inflammatory, hepatic, renal, and coagulation biomarkers were likewise investigated between AC and LC cohorts.

## Methods

### Study design, sample and data collection and definitions

The study was designed to include both AC inpatients and outpatients, treated at the Department of Internal Medicine, University Hospital Heidelberg. More specifically, we recruited patients who had a confirmed SARS-CoV-2 infection between 22nd February 2020 and 18th April 2020 and who continued to experience persistent symptoms at 5, 9, 12, 16, and 20 months (M) after the AC infection. By the time the study was enrolled (early 2020), there was no official definition and inclusion criteria for LC.

The Ethics Committee reviewed and approved the study protocol at the Medical Faculty of Heidelberg University and the University Hospital Heidelberg (Ethics approval number S-546/2020). We ended up collecting a total of 426 prospectively followed LC serum samples; 95, 73, 95, 78, and 85 were collected at 5 M, 9 M, 12 M, 16 M, and 20 M, respectively, and stored at −80 °C.

**Fig. 1:**
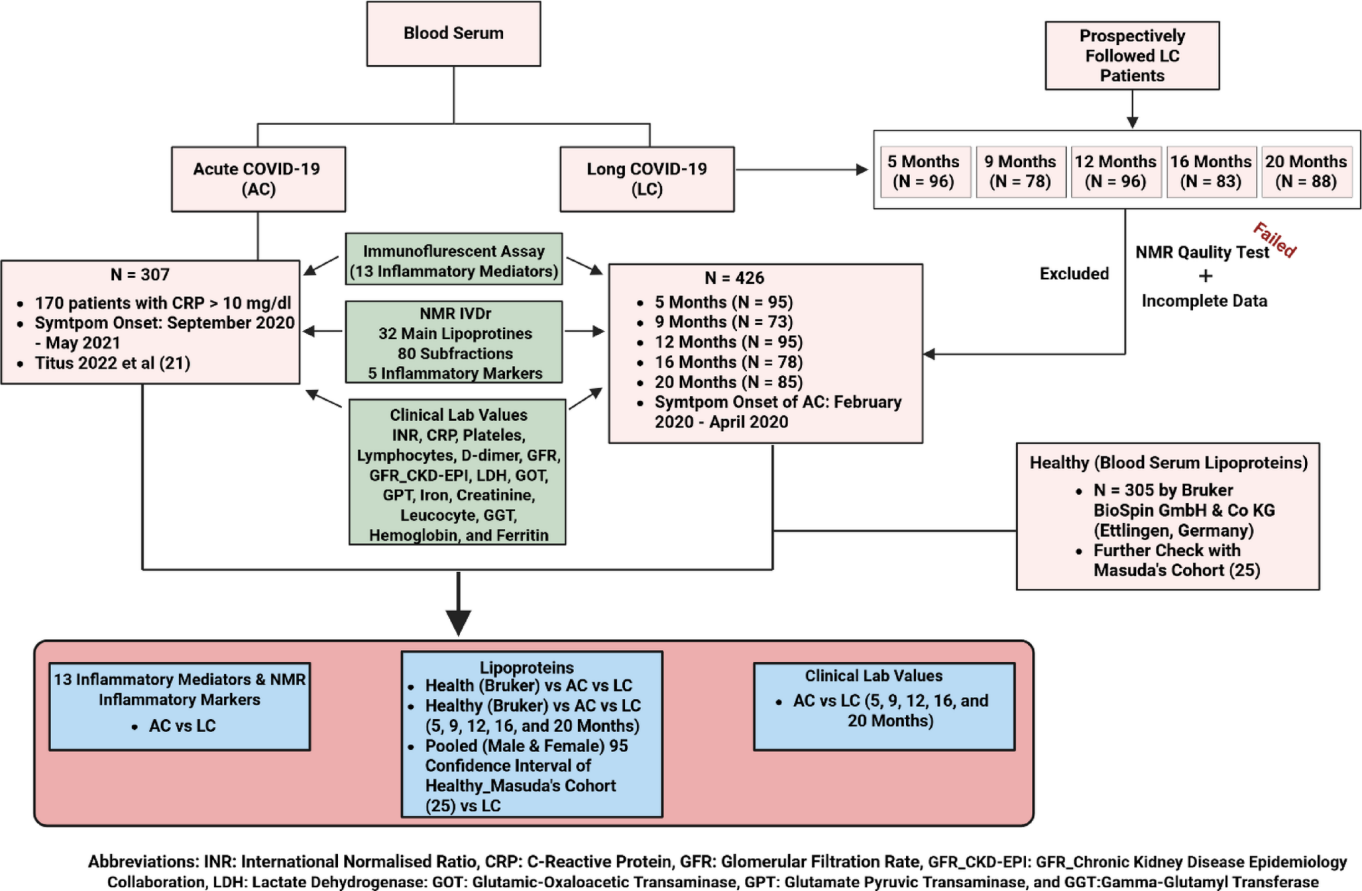
Summary of the study design for investigating the lipoproteomic and inflammatory profile of long COVID-19 (LC) cohort. Overview on data collection of each independent healthy, acute (AC), and LC cohorts for identification of lipoproteins, NMR-based inflammatory parameters, and pro- and anti-inflammatory mediators in LC cohort

Initially, a total of 194 AC patients donated blood where 80 and 60 out of patients were drawn blood twice and three times, respectively (Rössler et al., 2022). We collected NMR IVDr analytes (32 main lipoproteins, 80 subfractions of the main lipoprotein, and 5 inflammatory parameters), the concentration of 13 cytokines (proinflammatory mediators: interleukin (IL)-1β, interferon (IFN)-α2, IFN-γ, tumor necrosis factor (TNF)-α, IL-8, IL-12p70, IL-17 A, IL-23, IL-33, monocyte chemoattractant protein (MCP)-1 (CCL2/MCP-1) IL-6, IL-18, anti-inflammatory cytokine: IL-10) and clinical lab values (Method: 2.2. Clinical data collection of acute and long COVID-19 cohorts.) of 307 unvaccinated AC samples (170 patients) with CPR > 10 mg/dl. Of note, the symptom onset was between September 2020–May 2021 (Rössler et al., 2022). We also involved the IVDr data sets of 305 serum of the prehistoric COVID-19 healthy cohort that were provided by Bruker BioSpin GmbH & Co KG (Ettlingen, Germany). Furthermore, we referred to additional concentration of main lipoproteins of the healthy cohort, identified by Masuda et al. (Masuda et al., 2023). Of note, we summed up the data workflow and analysis in Fig. 1.

### Clinical data collection of acute and long COVID-19 cohorts

We collected the clinical data of AC and LC cohorts, such as international normalized ratio (INR), C-reactive protein (CRP), blood cell counts (platelets, lymphocytes, leucocytes), D-dimer, and glomerular filtration rate (GFR) calculated using the formula from chronic kidney disease epidemiology collaboration (GFR CKD-EPI), hemoglobin, ferritin, lactate dehydrogenase (LDH), glutamic-oxaloacetic transaminase (GOT), iron, creatinine, glutamic-pyruvic transaminase (GPT), and gamma-glutamyl transferase (GGT). Moreover, we gathered data of ANA titers and reduced exercised capacity of LC cohort. Details of each study assessment is found elsewhere (Rössler et al., 2022; Seeßle et al., 2022).

### IVDr SOPs-based sample Preparation of long COVID-19 serum

We thawed serum of LC at room temperature for 30 min and pipetted 150 µL of the serum and Bruker Plasma Buffer into a 1.5 ml Eppendorf tube. Then, we transferred 200 µL of the mixture into a 3 mm NMR tube for measurement and quantification.

### ^1^H-NMR spectroscopy equipment and spectral acquisition of long COVID-19 serum

We evaluated a total of 426 LC serum samples with a 600 MHz IVDr NMR system (Bruker Avance III HD 14.10 Tesla) that was operated with a triple resonance room temperature 3 mm probe at 310 K. We ran a nuclear overhauser spectroscopy experiment for 4 min, to quantify 112 lipoproteins. In addition to lipoproteins, we briefly discussed the biological role in Table S2. We also quantified the inflammatory parameters with a sequence of pulse gradient perfect echo experiment (1D-PGPE). These were GlycA, GlycB, Glyc (addition of GlycA and GlycB), supramolecular phospholipids composite (SPC), and Glyc/SPC. Of note, we excluded samples that failed to pass quality control tests even after we re-ran the experiments, and the quantified parameters were normalized to the sample volume.

### Quantification of 13 cytokines in long COVID-19 serum

To estimate cytokines by flow cytometry, we used 13-plex inflammation panel I kit (LEGENDplex™ Human Inflammation Panel 1 (13-plex) #740809, BioLegend, USA). We pipetted 25 µL of the serum and mixed them with 25 µL of assay buffer as recommended by manufacture and described earlier by us (PMID: 37228606). We then transferred 25 µL of 13-plex beads to a 96-well microplate. To allow the binding of the 13 cytokines to an antibody-conjugated capture bead, we incubated and shook the microplate for 2 h at room temperature (800 rpm). We added 25 µL of biotinylated detection antibodies after washing the plate. We subsequently added 25 µL of Streptavidin–phycoerythrin and incubated for 1 h. Lastly, we washed the beads and used a flow cytometer to quantify the concentration of each cytokine based on the standard curve using LEGENDplex™ data analysis software (BioLegend, USA). Of note, the biological functions of 13 cytokines are described in Table S3. Moreover, accuracy of serum measurement for IFN-α was indicated by the strong correlation between IFN-induced gene expression and serum IFN-α level (Kim et al., 2023).

### Data mining of the quantified and clinical lab values

We removed NMR lipoprotein and inflammatory parameters of AC and LC cohorts that have missing values of more than 50%. Herein, the missing value reflects that the signal of such parameters was not detected in the respective serum spectrum. Afterwards, we addressed missing values of the NMR parameters by imputing a low constant value on MetaboAnalyst 6.0 (Ewald et al., 2024). The same strategy was applied to healthy cohort. Moreover, we imputed a total of 12 missing body mass index (BMI) scores based on montone and linear regression method with IBM SPSS statistics version 28.0.0.0 (190). To remove skewness and heteroscedasticity of 13 cytokines concentration, we replaced a value that was lower than the limit of detection with 0 and applied cube-root transformation. The minimum detectable concentration of each cytokine is described in Table S5.

### Statistical analysis

We performed comparative statistics using both MetaboAnalyst 6.0 (Ewald et al., 2024), Prism software version 10.1.1 (323), and IBM SPSS statistics version 28.0.0.0 (190). Following F and normal distribution tests, we ran Mann–Whitney test for a comparison of the cytokines between 2 groups (AC vs. LC = 12 M + 16 M + 20 M) and NMR inflammatory parameters between 2 groups (AC vs. LC = 5 M + 9 M + 12 M + 16 M + 20 M). We compared 112 lipoproteins in 2 separate designs; (1) we compared between healthy vs. AC vs. LC (5 M + 9 M + 12 M + 16 M + 20 M) and (2) between healthy vs. acute vs. each time point of LC. Herein, we used one-way analysis of variance (ANOVA); ordinary ANOVA, Brown Forsythe & Welch ANOVA, and Kruskal Wallis tests following a normal distribution and equality of variance tests. We used the same statistics to compare NMR inflammatory and cytokines parameters in the same manner except clinical lab values (Method: 2.2. Clinical data collection of acute and long COVID-19 cohorts.). We compared the lab values only between AC and each time point of LC. Of note, we corrected the p-value, including p-value of post-hoc tests of parametric and non-parametric ANOVA by false rate discovery (FDR) of the Benjamini, Krieger, and Yekutieli method, and considered it significant when corrected p-value or q-value (corrected p-value) was less than 0.05. We further checked our significant findings, such as NMR parameters and cytokines, with analysis of covariance (ANCOVA) and Quade nonparametric ANCOVA by IBM SPSS statistics version 28.0.0.0 (190) since we took account into that sex, BMI, and age were the major confounders. Of note, we ran Quade nonparametric ANCOVA with each covariate one by one, since ranks of the multiple covariates may lead to an inaccuracy of the test. Particularly, covariate-adjusted p-value < 0.05 was considered significant. In addition to confounder, we determined the effect of vaccination with the ANOVA tests in which we compared between the healthy, unvaccinated AC, unvaccinated LC, and quartiles of vaccinated LC cohorts. We merged each time point of LC cohort and calculated the quartiles based on the delta timepoint in days that was between the time point before and after vaccination. The details of quartiles-based vaccinated LC are described in Table S6.

### Further validation of lipoprotein parameters with healthy cohort from Masuda’s study

We referred to the concentration of the main lipoproteins in the previously reported healthy cohort by Masuda et al. (Masuda et al., 2023) and then calculated a pooled 95% confidence interval (Cl) (male + female) to compare with the lipoproteins identified in our LC cohort. Lastly, we acquired triglycerides (mg/dL), HDCH (mg/dL), cholesterols (mg/dL), and LDCH (mg/dL) that were determined in clinical routine tests and ran Spearman and Pearson correlation tests to highlight the sensitivity and validity of NMR lipoprotein parameters. We further calculated root mean square error (RMSE) and adjusted (adj) R^2^ and generated Bland-Altman plots to confirm the validation. We considered the agreement between two different methods when adjusted R^2^ is higher than 0.5 and RMSE is lower. Of note, we addressed 4 missing LDLC values, which were evaluated in a clinical routine test, by imputation based on montone and linear regression methods with IBM SPSS statistics version 28.0.0.0 (190).

### Re-stratification of long COVID-19 cohort based on rank of each inflammatory mediator

We examined each cytokine concentration of the healthy cohort (de Lemos et al., 2003; Ruchakorn et al., 2019) and transformed the raw concentration of LC cohort´s cytokine into rank data (1, 2, 3, and 4) with IBM SPSS statistics version 28.0.0.0 (190) where higher ranks indicate more intensified inflammatory phenotype. We performed one-way ANOVA; ordinary ANOVA, Brown Forsythe & Welch ANOVA, and Kruskal Wallis tests to find out the relation between lipoproteins and cytokines and corrected the p-value by FDR. We also ran two-sided linear by linear association test to determine the uniqueness of the re-stratified group. Herein, we checked the association of the clinical presentations (reduced exercise capacity and ANA_1:80 (state of autoimmunity) with the rank of each cytokine, and then based on sex and BMI using the same statistics. To check relation of the findings with age, we exploited Spearman and Pearson correlation tests following a normal distribution test since the expected count of age group did not meet the requirement to run the association test. Of note, a p-value < 0.05 was considered significant.

## Results

### Characteristics of long COVID-19 cohort from month 5 to 20 and the baseline difference between independent healthy, acute, and long COVID-19 cohorts

We first summarized the baseline of the independent healthy, AC, and LC cohorts (Table 1). The characteristics of LC patients are previously summarized up to 12 M by Seeßle et al. (Seeßle et al., 2022). We finalized the summary of these patients from 5 months up to 20 months after the exclusion based on NMR experiment quality test and incomplete data (Table S1 and Fig. 1). Of note, only 7 LC patients were vaccinated at month 12. Furthermore, the characteristics of AC cohort with CRP higher than 10 mg/dl are described in Supplementary Table S2.

**Table 1:** Demographics of the independent healthy, acute, and long COVID-19 patient groups

	Health (*N* = 305)	Acute (*N* = 307)	5 M (*N* = 95)	9 M (*N* = 73)	12 M (*N* = 95)	16 M (*N* = 78)	20 M (*N* = 85)
Sex (Male)	57.70%	56.03%	47.64%	39.72%	45.26%	47.43%	45.88%
Age Median (Min–Max)	54 (21–88)	59 (23–96)	57 (18–85)	57 (18–85)	57(18–85)	57 (18–85)	57 (27–78)
< 30	6.22%	1.30%	3.31%	4.10%	3.15%	2.56%	1.17%
31–40	14.42%	8.14%	9.96%	6.84%	9.47%	8.97%	9.41%
41–50	17.37%	16.28%	12.17%	10.95%	12.63%	11.53%	12.94%
51–60	35.08%	27.03%	44.31%	41.09%	41.05%	42.30%	43.52%
61–70	12.78%	30.61%	22.15%	21.91%	21.05%	21.79%	20%
71–80	8.85%	12.37%	12.17%	13.69%	11.57%	11.53%	12.94%
> 80	5.24%	4.23%	1.10%	1.36%	1.05%	1.28%	0%
BMI (kg/m^2^) Median (Min–Max)	25 (18–42)	28 (18–59)	26 (19–58)	25 (19–59)	26 (19–59)	25 (19–35)	26 (19–59)
< 18.5	0.98%	0.65%	0.00%	0.00%	0.00%	0.00%	0.00%
≥ 18.5–24.9	36.39%	15.30%	34.73%	39.72%	31.57%	37.17%	38.82%
≥ 25-29.9	47.21%	41.69%	41.05%	34.24%	40%	35.89%	36.47%
≥ 30	15.40%	42.34%	24.21%	26.02%	28.42%	26.92%	24.70%

Baseline of the independent healthy, acute, and long COVID-19 cohorts are described in percentage. A total of 307 serum blood samples were collected from 170 acute COVID-19 patients with CRP serum concentrations higher than 10 mg/dl.

### Validity of the lipoprotein parameters quantified by NMR-IVDr and clinical routine test

We validated TPTG, TPCH, LDCH, and HDCH assessed by NMR-IVDr and clinical routine tests, using correlation and Bland-Altman analysis, including adjusted adj R^2^ and RMSE. We observed the agreement between two different methods of the measurement (Figure S1A-H). We found adj R^2^ of each lipoprotein were higher than 0.5 and the respective RMSE was low. We also observed the absence of proportional bias, and outliers in the Bland-Altman. We determined that the outliers may represent patient-specific or may be due to the manual calculation of each lipoprotein in the clinical routine test without a quality control test, unlike NMR IVDr method.

### Long COVID-19 shows a discernible main lipoprotein profile compared with both acute COVID-19 and healthy cohorts

**Fig. 2:**
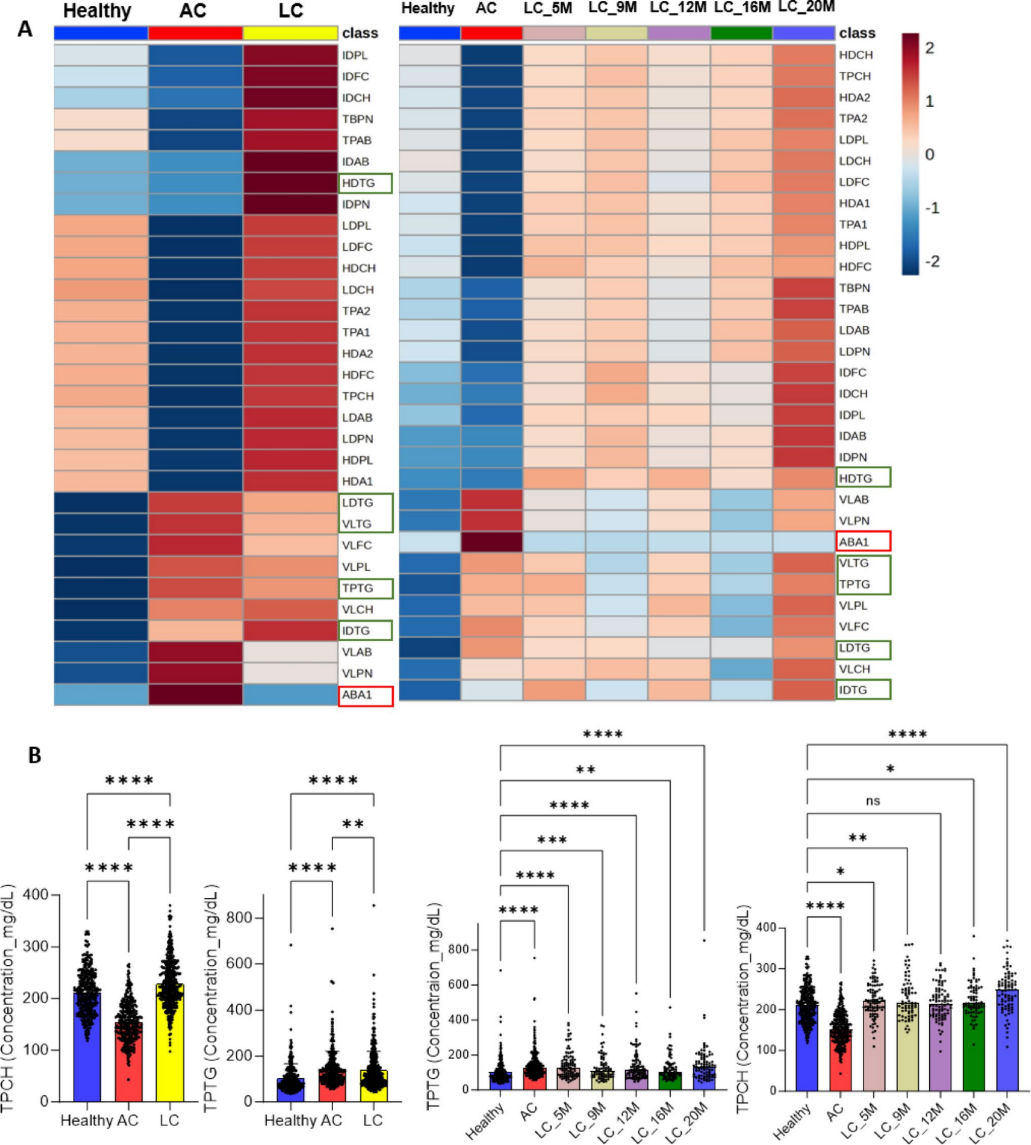
Hyperlipidemic phenotype in long COVID-19 (LC) patients for 20 months compared with acute COVID-19 (AC) and healthy cohorts. **A** Both left and right averaged heatmaps show 31 significantly altered main NMR lipoproteins between healthy, AC, and LC cohorts (adjusted p-value < 0.05 by one-way ANOVA; Ordinary ANOVA, Kruskal Wallis, and Brown Forsythe & Welch ANOVA tests). Heatmap (left) is the comparison between healthy, AC, and LC (5, 9, 12, 16, and 20 M merged). The average concentration of each lipoprotein parameter is displayed based on the color scale. Markers for the balance of pro and anti-inflammatory state and hypertriglyceridemia are highlighted in red and green boxes, respectively. **B** Total plasma cholesterol (TPCH) was significantly higher in LC cohort and over time than in healthy and AC cohorts. Total plasma triglycerides (TPTG) were found significantly higher in LC cohort and over time compared to healthy cohort, but it was significantly lower in contrast to AC cohort (post hoc test of Brown Forsythe & Welch ANOVA and Kruskal Wallis tests: q-value = NS > 0.05, *<0.05, **< 0.01, ***<0.001, ****<0.0001). **A** and **B** Healthy (*N* = 305), AC (*N* = 307), and LC (*N* = 426) involves the following subsets: 5 M (*N* = 95); 9 M (*N* = 73);12 M (*N* = 95); 16 M (*N* = 78); 20 M (*N* = 85)

We found 31 significantly altered main NMR lipoprotein parameters. We observed TPCH, LDCH, HDCH, TPA1, TPA2, TPAB, TBPN, IDPN, LDPN, IDTG, HDTG, VLCH, IDCH, IDFC, LDFC, HDFC, IDPL, LDPL, HDPL, HDA1, HDA2, IDAB, and LDAB were significantly higher in LC cohorts (all time points merged) than in AC and healthy cohorts (Fig. 2, Supplementary excel file, and Figure S2). We also identified TPTG, VLPN, VLTG, VLFC, VLPL, and VLAB significantly increased in LC cohort than in healthy cohort, but significantly lower in comparison to AC cohort, while LDHD (LD/HD) was similar between AC and healthy cohorts (Fig. 2 and Supplementary excel file). In addition to LDL parameter, LDTG was found significantly higher in LC cohort than in healthy cohort and the average concentration was lower than AC cohort. We observed the same observation in comparing healthy vs. acute vs. each time of LC; TPA1, TPA2, IDPN, IDCH, IDPL, HDTG, HDPL, HDA1, HDA2, and IDAB were significantly higher in all time points of LC cohort than healthy and AC cohorts. Furthermore, TPCH, LDCH, HDCH, TPA1, TPAB, TBPN, LDPN, IDFC, LDFC, HDFC, LDPL, IDAB, and LDAB were higher in all time points of LC cohort compared to healthy and AC cohorts, yet the statistical significance was not achieved at all time points (Fig. 2, Supplementary excel file, and Figure S2). Lastly, we highlighted lipoprotein parameters that indicate hypertriglyceridemia (Fig. 2A green boxes) and the balance of pro and anti-inflammatory state (Fig. 2A red box). TPTG, VLTG, LDTG, and IDTG were significantly higher in both AC and LC cohorts compared to healthy cohort, while HDTG was only found extraordinarily higher in LC cohort (Fig. 2A green boxes). We uncovered that ABA1 (AB/A1) was significantly higher in AC cohort and remained unchanged between healthy and LC cohorts (Fig. 2A red box).

### Impact of long COVID-19 on subfractions of each main lipoprotein

We identified 80 significantly altered subfractions of the main lipoproteins. Notably, L1PN, L2PN, L3PN, V3CH, V4CH, V1FC, V4FC, V3PL, L2TG, L1CH, L2CH, L3CH, L1FC, L2FC, L3FC, L6FC, L1PL, L2PL, L3PL, L1AB, L2AB, L3AB, H1TG, H2TG, H3TG, H4TG, H1CH, H2CH, H3CH, H1FC, H2FC, H3FC, H4FC, H1PL, H2PL, H3PL, H4PL, H1A1, H2A1, H3A1, H4A1, H1A2, H2A2, and H3A2 were significantly higher in LC cohort (all time points merged) than in AC and healthy cohorts (Fig. 2, Supplementary excel file and Figure S2). V1TG, V1CH, V2CH, V2FC, V3FC, V1PL, and L1TG, L6TG were significantly higher in LC cohort in the comparison to healthy cohort, while they were significantly lower than in AC cohort (Fig. 2, Supplementary excel file and Figure S2). We further saw some of the subfractions remain unaffected in LC compared to healthy cohort and significantly higher than in AC cohort. These were L4CH, L4FC, L4PL, L4AB, L4PN, L5PN, L6PN, V5FC, L5CH, L6CH, L5FC, L5PL, L6PL, L5AB, H4CH, and H4A2 (Fig. 2, Supplementary excel file, and Figure S2). Moreover, we revealed that L6AB remained unaffected between three cohorts, whileV5CH was significantly lower in LC than healthy and AC cohorts (Fig. 2, Supplementary excel file and Figure S2). Of note, the same result was obtained in the comparison between healthy vs. acute vs. each time point of LC.

### Dysregulated lipoprotein metabolism in acute COVID-19 cohort compared to healthy cohort

We identified TPTG, ABA1 (AB/A1), VLPN, VLTG, IDTG, LDTG, VLCH, VLFC, VLPL, and VLAB were significantly higher in AC cohort than healthy cohort, while we observed TPCH, LDCH, HDCH, TPA1, TPA2, TPAB, TBPN, LDPN, IDCH, IDFC, LDFC, HDFC, IDPL, LDPL, HDPL, HDA1, HDA2, and LDAB were significantly lower in AC cohort than in healthy cohort (Fig. 2, Supplementary excel file, and Figure S2). The rest of lipoproteins such as TPAB, IDPN, and IDAB did not pass the significance, however the average concentrations were lower in AC cohort compared to healthy cohort (Fig. 2, Supplementary excel file, and Figure S2). We found all the subfractions of LDL and VLDL with triglycerides were significantly higher in AC cohort than in healthy cohort. Moreover, we determined larger and less dense subfractions of VLDL with cholesterols and free cholesterols (V1CH, V2CH, V3CH, V1FC, V2FC, and V3FC) were significantly higher in AC cohort than in healthy cohort (Fig. 2, Supplementary excel file, and Figure S2). We also revealed the average concentration of smaller and denser subfractions of VLDL with cholesterols (V4CH and V5CH) were lower in AC cohort compared to healthy cohort. We found the same observation in V4FC, however AC cohort showed significantly higher level of V5FC than healthy cohort (Fig. 2, Supplementary excel file, and Figure S2). Interestingly, although we observed HDTG remained unaffected between AC and healthy cohorts (Fig. 2, Supplementary excel file, and Figure S2), subfractions of such main lipoprotein were significantly different between the cohorts. We identified AC cohort exhibited significantly lower level of H4TG and higher level of H2TG and H3TG in comparison to healthy cohort. Moreover, the average concentration of the largest and least dense HDL with triglyceride (H1TG) was lower in AC cohort than in healthy cohort (Fig. 2, Supplementary excel file, and Figure S2). Rest of the lipoprotein subfractions such as L1PN, L2PN, L3PN, L4PN, L5PN, L1CH, L2CH, L3CH, L4CH, L5CH, L6CH, L1FC, L2FC, L3FC, L4FC, L5FC, L6FC, L1PL, L2PL, L3PL, L4PL, L5PL, L6PL, L1AB, L2AB, L3AB, L4AB and L5AB were significantly lower in AC cohort than in healthy cohort regardless of their size and density (Fig. 2, Supplementary excel file, and Figure S2).

### Further confirmation on hyperlipidemia metabolism in long COVID-19 cohort

**Fig. 3:**
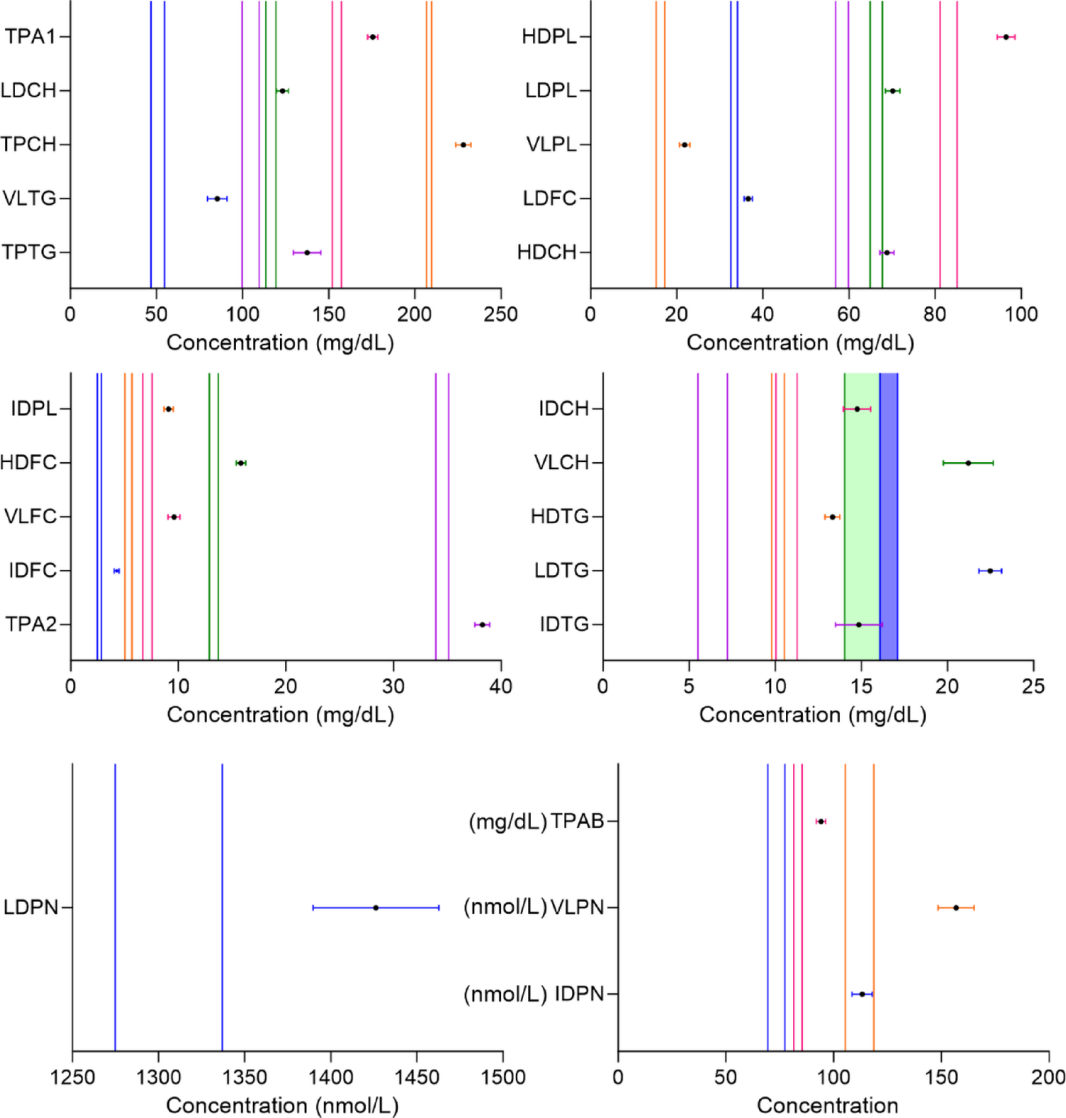
Dysregulated lipoprotein metabolism toward hyperlipidemia in long COVID-19 (LC) cohort. Forest plots consist of each main NMR lipoprotein parameter (*TPA1* total plasma apolipoprotein-A1, *LDCH* LDL-cholesterol, *TPCH* total plasma cholesterol, *VLDL* VLDL-triglycerides, *TPTG* total plasma triglycerides, *HDPL* HDL-phospholipids, *LDPL* LDL-phospholipids, *VLPL* VLDL- phospholipids, *LDFC* LDL-free cholesterol, *HDCH* HDL-cholesterol, *IDPL* IDL-phospholipids, *HDFC* HDL-free cholesterol, VLFC-VLDL-free cholesterol, *IDFC* IDL-free cholesterol, *TPA2* total plasma apolipoprotein-A2, *IDCH* IDL-cholesterol, *VLCH* VLDL-cholesterol, *HDTG* HDL- triglycerides, *LDTG* LDL-triglycerides, *IDTG* IDL-triglycerides, *LDPN* LDL particle number, *TPAB* total plasma apolipoprotein-B100, *VLPN* VLDL particle number, and *IDPN* IDL particle number) where each vertical line represents a pooled (sex-merged) 95% confidence interval of the respective lipoprotein of the healthy cohort (Masuda et al., 2023). The corresponding lipoprotein parameters of long COVID-19, are illustrated as mean and 95% confidence interval and are displayed with different colors (5, 9, 12, 16, and 20 M merged). The hyperlipidemic phenotype of long COVID-19 was further determined

We first merged all the time points of LC, considering the data from both female and male subjects. Similarly, we calculated pooled 95% Cl based on the results reported by Masuda et al. (Masuda et al., 2023). We found the concentration of main lipoproteins in LC, such as TPCH, TPTG, HDCH, LDCH, IDCH, VLCH, HDTG, IDTG, LDTG, VLTG, HDFC, LDFC, IDFC, VLFC, HDPL, LDPL, IDPL, VLPL, LDPN, IDPN, VLPN, TPA1, TAP2, and TPAB did not fall within a pooled 95% CI of the respective main lipoprotein in healthy cohort (Table S7 and S8). We first merged all the time points of LC and then generated forest plots. Each vertical line represented a pooled 95% Cl of the respective lipoprotein of the healthy cohort and the plots showed 95% Cl and mean of each main lipoprotein in LC cohort, with different colors (Fig. 3).

### Inflammatory biomarkers in acute and long COVID-19 cohorts

**Fig. 4:**
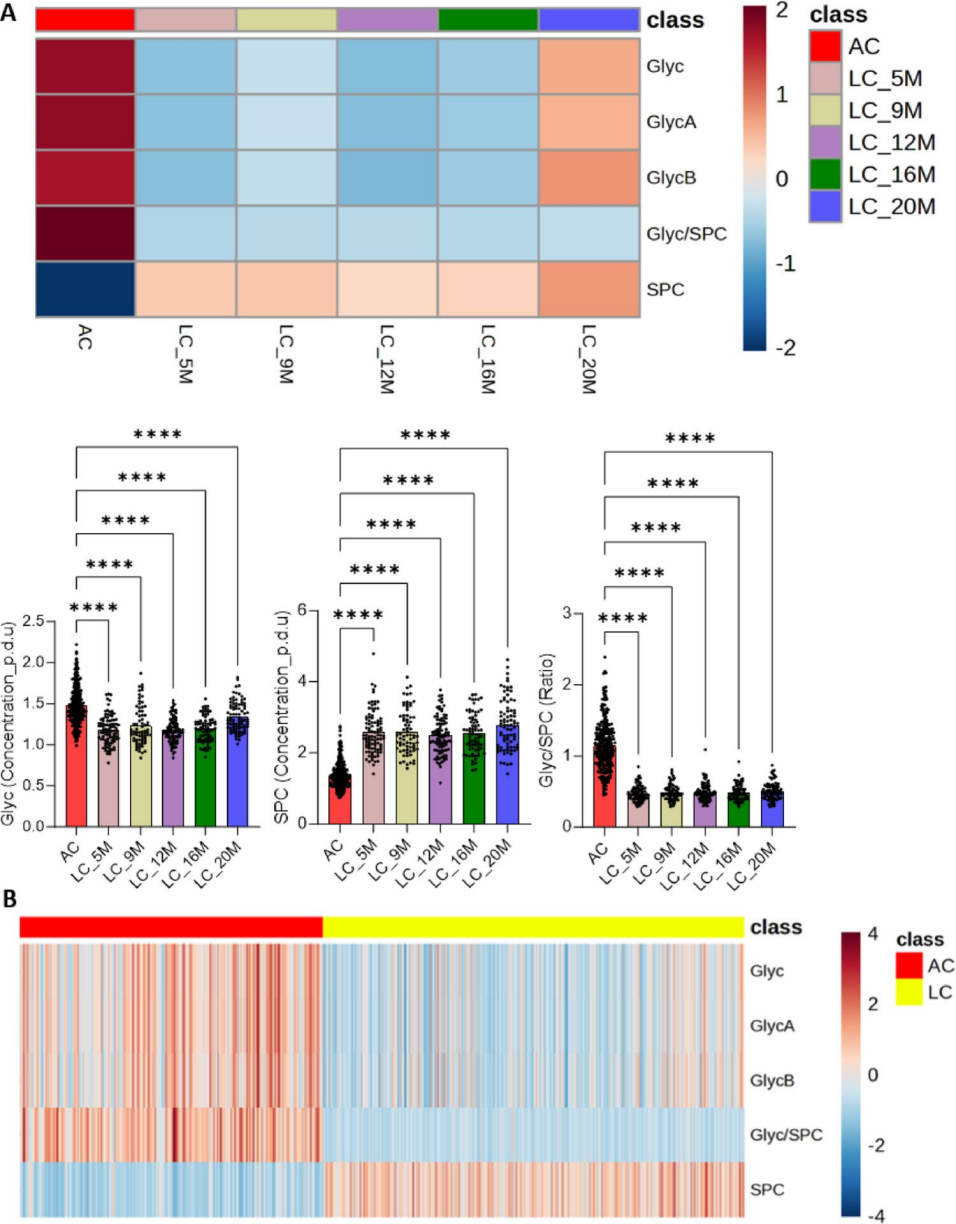
Discernible active inflammatory state in acute COVID-19 cohort. **A** Average Heatmap shows that 5 inflammatory NMR parameters were significantly lower at each time point of long COVID-19 (LC) cohort in contrast to acute COVID-19 (AC) cohort (adjusted p-value < 0.05 by one-way ANOVA; Kruskal Wallis and Brown Forsythe & Welch ANOVA tests). The average concentration of each inflammatory parameter is displayed based on the color scale. Glyc (GlycA + GlycB), supramolecular phospholipid composite (SPC), and Glyc/SPC were significantly higher, lower, and higher in acute COVID-19 cohorts compared to each time point of LC cohort, respectively (post hoc test of Brown Forsythe & Welch ANOVA and Kruskal Wallis tests: q-value = *<0.05, **< 0.01, ***<0.001, ****<0.0001). **B** Heatmap shows that 5 significantly higher inflammatory NMR parameters in ACcohort in the comparison to LC (5, 9, 12, 16, and 20 M merged) cohort. The average concentration of each inflammatory parameter is displayed based on the color scale (adjusted p-value < 0.05)

**Fig. 5:**
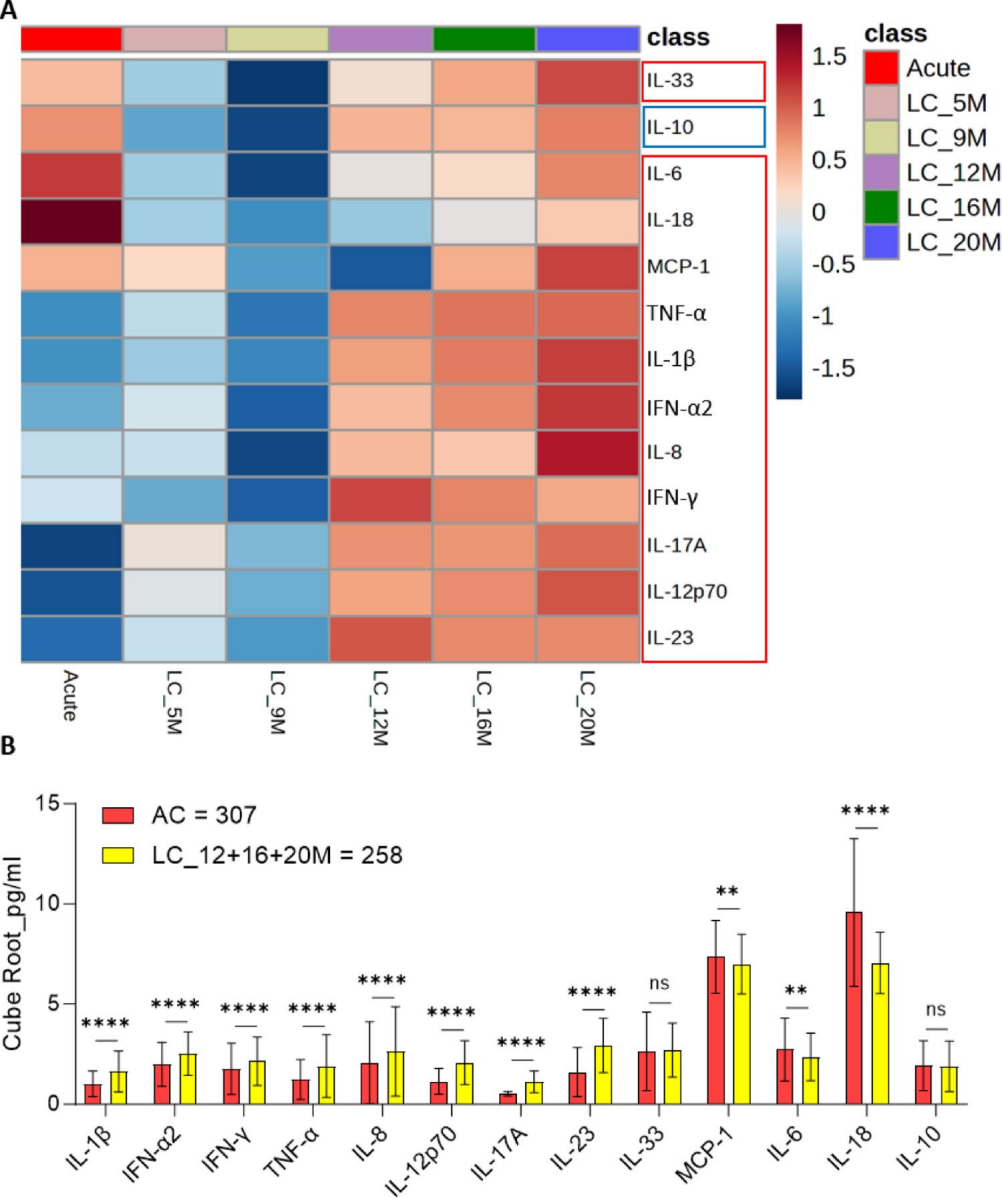
Development of progressive low-grade inflammation in long COVID-19 (LC) cohort. **A** The averaged heatmap shows 12 significantly altered pro-inflammatory (red boxes) anti-inflammatory (blue box) mediators between acute and each time point of LC cohorts (adjusted p-value < 0.05 by Kruskal Wallis test). The average concentration of each cytokine is displayed based on the color scale. **B** The evaluated cytokines were significantly higher in long COVID-19 cohort (12, 16, and 20 M merged) than acute COVID-19 cohort except for monocyte chemoattractant protein (MCP)-1, interleukin (IL)-6, and IL-18, while the anti-inflammatory cytokine IL-10 was similar across the two groups (Mann-Whitney test: q-value = NS > 0.05, *<0.05, **< 0.01, ***<0.001, ****<0.0001)

We identified NMR-based proinflammatory parameters, which were significantly higher in AC cohort than in LC cohort and each time point of LC. (Fig. 4 and Supplementary excel file). From the cytokine panel, we found LC cohort exhibited IL-1β, IFN-α2, IFN-γ, TNF-α, IL-8, IL-6, IL-33, and IL-10 started to be discernibly higher from 12 M and so on, while we observed MCP-1 and IL-18 increased higher from 16 M. However, IL-18 was significantly lower over time in LC cohort than AC cohort. We discovered a similar trend in IL-6 and MCP-1 that were significantly lower until 12 M than AC cohort and the average of IL-6 and MCP-1 was still lower onwards and higher, respectively (Fig. 5, Table S9, and Supplementary excel file) We also observed IL-1β, IFN-α2, IFN-γ, TNF-α, and IL-8 were significantly higher from 12 M onwards. Notably, IFN-γ and IL-8 were significantly lower in LC cohort at 9 M compared to AC cohort. Moreover, we revealed that IL-12p70, IL-17 A, and IL-23 were significantly higher in LC cohort over time than AC cohort whilst the IL-33 and IL-10 exhibited a similar trend (Fig. 5, Table S9, and Supplementary excel file). We then compared AC cohort with pooled concentrations from the 12 M + 16 M + 20 M time intervals of LC, to identify the extent of inflammation. We disclosed IL-1β, IFN-α, IFN-γ, TNF-α, IL-8, IL-12p70, IL-17 A, and IL-23 were significantly higher in LC cohort than in AC cohort, while CCL2/MCP-1, IL-6, and IL-18 were significantly lower than in AC cohort (Fig. 5 Table S9, and Supplementary excel file). Finally, the anti-inflammatory cytokine “IL-10” was significantly lower in LC cohort until month 9 compared to AC cohort. However, the cytokine concentration was similar between the AC and LC (pooled IL-10 level from the 12 M + 16 M + 20 M) cohorts (Fig. 5 Table S9, and Supplementary excel file).

### Re-stratified long COVID-19 cohort according to rank of each cytokine and its clinical presentations

We re-stratified our LC cohort, including all time points, based on the rank of each cytokine. We found rank of MCP-1 (Figure S5A & Figure S3), IL-6 (Figure S3), IL-18 (Figure S3), and IL-23 (Figure S3) could stratify our LC cohort, while the rest of the rank of cytokines could not achieve refined stratification (Figure S4). These results clearly suggest that the lipoprotein metabolism of LC cohort was highly influenced by inflammation. We also revealed that ANA_1:80 was significantly associated with the rank of MCP-1 (Figure S5B), IL-6 (Figure S6A), and IL-23 (Figure S6A), while the rank of IL-18 (Figure S6A) was only significantly associated with reduced exercise capacity. We further examined the association based on sex, BMI, and age. We observed females of each ranked cytokine group tended to be associated with reduced exercise capacity and ANA_1:80 (Figure S5C and Figure S6B). We also determined BMI was only associated with reduced exercise capacity in the lower rank of each cytokine group, while BMI was not associated at all with ANA_1:80 except the 4th rank of MCP-1 (Figure S5D and Figure S7). Yet, we considered it was due to a low N number of the category in the group. Moreover, we demonstrated age was positively (*r* = 0.22) in 1st rank of IL-6 ANA_1:80 and negatively (*r* = -0.23) correlated with ANA_1:80 in 3rd rank of IL-18 (Table S10). However, we supposed the correlation values were negligible. Next, we performed the same analysis on the AC cohort and further suggested a link between inflammation and lipid metabolism in AC (Figure S8 &Table S11).

### Inability of standard clinical parameters to capture the derangements of long COVID-19

We compared INR, CRP, platelets, lymphocytes, D-dimer, and GFR CKD-EPI of AC cohort with LC cohort. Of note, we merged sex since the baseline of each clinical lab parameter to male and female is the same. INR, CRP, and D-dimer were significantly higher in AC cohort than in LC cohort, while platelet and lymphocyte counts were significantly lower than LC cohort. Therefore, almost all of our LC cohort had a physiological level of such lab values (Figure S9). Moreover, we found GFR CKD-EPI was similar between the cohorts (Figure S9). We compared the other additional lab values, such as LDH, GOT, iron, creatinine, GPT, leucocyte, hemoglobin, ferritin, and GGT between AC and LC cohorts based on sex. Similarly, these lab values of our LC cohort fell within the established physiological reference ranges (Figure S9).

### The effect of sex, BMI, and age on the significant findings and the association of vaccination with inflammation and lipoprotein metabolism

As lipoprotein is relevant to BMI, we ran ANOVA tests first. We observed that AC cohort displayed significantly higher BMI compared to healthy and long COVID-19 cohorts. Moreover, BMI was not significantly different between healthy and LC cohorts (Figure S10). We found all the NMR parameters, such as proinflammatory and lipoprotein parameters remained significant in the comparison between healthy, acute, and LC cohorts even after adjusting to sex, BMI, and age, where we included LC cohort as 5, 9, 12, 16, and 20 M merged and time point based separately (Figure S12). We also observed that all the proinflammatory and anti-inflammatory mediators remained significant between AC and LC cohorts and AC and all time points of LC. However, we saw only IL-6 was no longer significant in the comparison between acute and long COVID-19 (12 + 16 + 20 M merged) after adjusting to BMI. We further ran the covariate analysis to check whether the re-stratified long COVID-19 cohorts maintained the impact. We revealed the group effect of MCP-1 on V5TG, L6CH, L6PL, H1CH, and H1PL did not remain significant after adjusting to sex. Similarly, V5TG, L3TG, and L4TG, and L3FC were not significant with age and BMI adjustment, respectively. Additionally, we identified TPA1, ABA1 (AB/A1), VLFC, HDA1, L5PN, V3TG, V4TG, V1CH, V2FC, V3FC, L2PL, L5PL, L5AB, H3CH, H2FC, H2PL, H3PL, H2A1, H3A1, and H1A2 were not significantly relevant to the group effect of IL-18 after adjusting to sex. We also demonstrated that both H3PL and H3A1 were not significant between the rank of IL-18 with adjustment to age and BMI. Lastly, we found vaccination might induce more intensified inflammatory phenotype in the quartile of vaccinated LC cohort compared to unvaccinated LC cohort, since the alteration of lipoprotein metabolism was regardless of the vaccination (Figure S11).

## Discussion

The present study demonstrates hyperlipidemia and chronic inflammation as hallmarks of LC. In fact, LC was associated with a distinct lipoprotein profile compared with both AC and healthy cohorts. Concerning inflammation, the analysis of NMR-based inflammatory biomarkers and traditional inflammatory mediators, including cytokines and chemokines, indicated a progressive inflammatory phenotype in LC cohort. Of interest, patient stratification into different categories of inflammation severity showed consistent changes in the lipoprotein metabolism. Taking together, our results suggest that lipoprotein metabolism varied with the extent of inflammation.

### Development of hyperlipidemia in long COVID-19 alongside chronic inflammation

Our results demonstrate fluctuations in the lipoproteins in LC at some time points but become consistent from month from month 5 to month 20. The fluctuating lipoproteins TPTG, VLAB, VLPN, VLTG, VLPL, VLFC, VLCH, IDTG, and LDTG, whereas we found HDCH and LDCH consistently higher in LC cohort compared to healthy cohort. We postulate such shifts were associated with the activity of cholesterol ester transfer protein (CETP). A high rate of the protein activity promotes the transfer of cholesteryl esters from HDL to apolipoprotein B-100 (Apo B-100)-containing lipoproteins, resulting in a decrease in HDCH and an increase in cholesterols of lipoproteins (Brousseau et al., 2004; Tatò et al., 1995). Moreover, the production rate of VLDL-Apo B-100 decreases, VLPN become more prone to hepatic uptake, and the distribution rate of phospholipids is influenced by a low rate of the protein activity (Barter et al., 2007; van der Tuin et al., 2015; Watts et al., 2006). Recent studies show COVID-19 convalescents had a higher level of CETP than the healthy cohort and lipoprotein lipase was impaired by the elevated IL-6 and TNF-α in addition to increased hepatic lipase in the LC cohort(Almulla et al., 2025; Pushalkar et al., 2024). Hence, we suggest that CETP and hepatic lipase activity was still low, resulting to abnormal levels of the lipoproteins in our LC cohort.

Nevertheless, we observed the main lipoprotein levels were consistently higher over time after AC infection in comparison to healthy cohort Our finding is in line with the results reported by Bizkarguenaga et al. (Bizkarguenaga et al., 2022). They examined on recovered AC patients with NMR IVDr, where the average recovery time was 6 ± 2 months: two clusters, such as Recovered cohort (RE)_I and RE_II were revealed. They found RE_I exhibited almost all the lipoproteins were at the normal physiological level as in the healthy cohort except the subfractions of VLTG, VLFC, and VLPL, while RE_II was distinct from the RE_I and healthy cohort. RE_II showed higher concentrations of the lipoproteins with triglycerides, cholesterol, and phospholipids and lower concentrations of VLPL, VLPL1-5, VLCH, VLCH1-5, VLFC, VLFC1-5, VLTG, VLTG1-5, and IDTG compared to the healthy cohort and RE_I, respectively. However, we found these lipoproteins were higher over time in LC compared to healthy cohort except the VLDL-5. We identified that VLDL5 with phospholipid, triglycerides and free cholesterol fluctuated over time and V5CH was consistently lower in LC compared to healthy and AC cohorts.

We further validated our finding with the results identified by Masuda et al. (Masuda et al., 2023). Therefore, we observed persistent hyperlipidemic phenotype was not by random chance in our LC cohort, suggesting a potential link between lipoprotein metabolism and LC pathogenesis. Our findings are in accordance with the previous findings. Roccaforte et al. found the levels of TPCH, TPTG, LDCH, and HDCH were higher in the recovery cohort than in AC cohort (Roccaforte et al., 2020). Similarly, Uyaroğlu et al. identified such lipoproteins highly increased than at the time of admission by AC infection, while a 3–6 months follow-up study concluded LDCH and HDCH were higher than AC cohort with severe and critical cases (Li et al., 2021; Uyaroğlu et al., 2022). In contrast to our findings, the LC cohort tended to have a lower level of HDCH at month 12 (Xu et al., 2023). Moreover, the recovery and severity of the LC cohort was associated with decreased and increased levels of HDCH and ferritin, respectively (Al-Zadjali et al., 2024; Ansone et al., 2024), which indeed highlights multifaceted heterogeneity of the disease. We postulate an extremely high level of HDCH may be linked to chronic inflammation in our LC cohort. Previous findings inferred HDCH turned into a proinflammatory stimulus during acute phase response and facilitated in expression and secretion of MCP-1 (Tölle et al., 2012; Van Lenten et al., 1995). An extremely high level of HDCH was also found to be more proinflammatory in coronary artery disease cohort compared to those with a normal level of HDCH and relevant to mortality of cardiovascular diseases (Ansell et al., 2003; Madsen et al., 2017). These collective results further support our hypothesis that we identified the link of lipoproteins with MCP-1, IL-6, IL-18, and IL-23 in LC cohort and lipoprotein metabolism altered between AC and LC cohorts, suggesting a shift of the lipoprotein metabolism in states of acute and chronic inflammation.

### Development of low-grade inflammation after recovery from acute COVID-19 infection in long COVID-19 cohort

To date, the mechanism of LC is not fully elucidated. Several studies have suggested that epigenetic modification of immune cells induce immune dysregulation toward chronic inflammation in such patients (Balnis et al., 2022; Cunha et al., 2020; Liu et al., 2023; Ryan et al., 2022). Our findings further favor these studies. We identified NMR-based inflammatory parameters and ABA1 (AB/A1) were lower in LC cohort than in AC cohort. Moreover, CRP remained unchanged over the time point in long COVID-19 cohort. Hence, we determined such parameters were only relevant to AC phase infection, suggesting the association with acute inflammation. Such findings are in accordance with previous observations, including the connection between the parameters and CRP (Kazenwadel et al., 2023; Rössler et al., 2022).

Our results demonstrate fluctuations in the inflammatory profile of LC at some time points but become consistent from month 12 to month 16. We explain this by different individual disease-courses next to cytokine neutralization. Chronic inflammation can impair the immune system, potentially leading to secondary immunodeficiency and/or autoimmunity (Fleit, 2014; Tuano et al., 2021; Wang & DuBois, 2015). In our study, IL-6 and MCP-1 concentrations were lower in the LC cohort compared to AC patients at month 5 and 9. IFN-γ and IL-8 exhibited a similar pattern, with reduced levels compared to AC at month 5 and 9. Moreover, IL-18 was consistently lower in LC cohort than in AC cohort across all time points. These findings may reflect cytokine neutralization mechanisms, a process where naturally produced auto-antibodies target certain chemokines. This phenomenon has been described in Long COVID patients and correlate with a favorable disease course (Muri et al., 2022). Furthermore, MCP-1 and IL-18 were lower in LC relative to the what observed in recovered LC individuals, while the level of IL-6, IFN-γ, and IL-8 decreased in the LC cohort compared to the healthy cohort (Kovarik et al., 2023; Williams et al., 2022). However, in our LC cohort IFN-γ and IL-8 started to increase from month 12. We also found higher concentrations of IL-1β, IFN-α2, TNF-α, IL-12p70, IL-17 A, and IL-23 in LC cohort than in AC cohort, whilst the anti-inflammatory cytokine IL-10 was similar between AC and LC cohorts. Our observations are in line with previous results. The LC cohort showed increased levels of IL-1β, IFN-γ, IL-8, IL-12p70, and IL-17 A than healthy cohort (Gomes et al., 2023; Ong et al., 2021; Phetsouphanh et al., 2022; Schultheiß et al., 2022; Woodruff et al., 2023). Furthermore, IFN-α was higher in the LC cohort than in those who have recovered from COVID-19 infection without any persistent clinical symptoms and the LC cohort showed a higher level of I and III IFN up to 8 months together with prolonged activation of plasmacytoid dendritic cells (Phetsouphanh et al., 2022; Queiroz et al., 2024). Other researchers compared acute covid vs. 2 months vs. 6 months after hospital discharge cohorts (Andrejčinová et al., 2024), revealing that the level of IL-23 remained stable between the time points but the T-cells level was persistently elevated. In addition, a recent study demonstrated that IL-23 inhibitor treatment has the potential to lower the risk of the AC and LC cohort (Hu et al., 2024).

We found IL-6, IL-18, and MCP-1 were overall higher in AC cohort than in LC cohort, despite the progressive elevations observed over the latest time points. Our result is consistent with previous findings. The LC cohort had a decreased level of IL-6 compared to the AC cohort (Andrejčinová et al., 2024; Queiroz et al., 2022; Yin et al., 2023) but higher in contrast to the healthy cohort (Gomes et al., 2023; Woodruff et al., 2023). Moreover, it was found that IL-6 was one of the key cytokines in providing class separation between the LC and asymptomatic healthy cohorts at month 8 (Phetsouphanh et al., 2022). IL-18 also exhibited a similar pattern. IL-18 increased in the AC cohort more than the LC cohort and some of the patients showed increased concentrations even 6 months after AC infection (Andrejčinová et al., 2024). Such findings further solidify our result that we revealed IL-18 increased along with IFN-γ. IL-18 is known to enhance the production of IFN-γ and a recent study concluded that increased IFN-γ level of the LC cohort was promoted by T-cell-mediated peripheral blood mononuclear cells (Krishna et al., 2024; Nakanishi, 2018). Similarly, MCP-1 was found unchanged between the LC and healthy cohorts at month 4, while its level remained unaffected up to 12 months in the LC cohort (Phetsouphanh et al., 2022; Santopaolo et al., 2023). Yet, Schultheiß et al. showed CCL2/MCP-1 prominently increased in the LC cohort than in healthy cohort in which the range of sampling time point was from up to 17 months (Schultheiß et al., 2023). These results collectively consolidate our finding that CCL2/MCP-1 started to be higher from month 16 and so on in LC cohort.

### The interplay of vaccination, lipoprotein metabolism and inflammation in long COVID-19 cohort

The prevailing understanding is that vaccination is effective against the development of LC. Yet, the effectiveness is enhanced when the vaccination occurs before AC infection. Several epidemiological results show a 34–36% reduction in the risk compared to unvaccinated individuals before AC infection. (Brannock et al., 2023; Lundberg-Morris et al., 2023; Trinh et al., 2024). 7 LC patients were vaccinated at month 12 before AC infection and pro & anti-inflammatory mediators were significantly higher in 12–20 months compared to 5–9 months in our study. Herein, we suggest that alteration of pro and anti-inflammatory mediators across the delta groups (quartiles) may reflect the influence of other factors, rather than a solely vaccination effect and vaccination may have a beneficial effect on lipoprotein homeostasis before AC infection.

## Summary and conclusion

We performed a longitudinal study with human blood to uncover hallmarks of LC. We concluded that clinical laboratory test was not informative enough to define LC. Instead, we could phenotype LC with NMR-based lipoprotein analysis and a cytokine assay where we discovered persistent hyperlipidemic phenotypes for 20 M, alongside the development of low-grade inflammation. Our findings could contribute to improve the diagnosis, prognosis, and therapeutics for the disease. Further investigation is required to elucidate the mechanism of COVID-19 pathogenesis while considering the complex heterogeneity and re-stratification within a large population.

## Electronic supplementary material

Below is the link to the electronic supplementary material.

Supplementary Material 1

Supplementary Material 2

Supplementary Material 3

## Data Availability

Raw NMR spectra (NOESY & PGPE) were annotated with a commercial client-server approach as provided by Bruker BioSpin GmbH & Co. KG (https://www.bruker.com/de/products-and-solutions/mr/nmr-clinical-research-solutions/b-i-methods.html). We thereof did not annotate raw spectra ourselves and thus cannot match raw NMR signals with lipoprotein annotations. Instead of providing raw NMR data on a public repository, all individual patient and lipoprotein concentrations are provided as a spreadsheet supplement file “Springer_Metabolomics_Supplementary File_2_NMR Parameters_Raw Concentrations_Bae et al”. Of note, Healthy control raw concentrations cannot be shared since they are IP of Bruker BioSpin GmbH & Co. KG.

## Abbreviations

ABA1 or AB/A1: Apolipoprotein-B100/Apolipoprotein-A1
H1A1: Apolipoprotein-A1 HDL-1
H1A2: Apolipoprotein-A2 HDL-1
H1CH: Cholesterol HDL-1
H1FC: Free Cholesterol HDL-1
H1PL: Phospholipids HDL-1
H1TG: Triglycerides HDL-1
H2A1: Apolipoprotein-A1 HDL-2
H2A2: Apolipoprotein-A2 HDL-2
H2CH: Cholesterol HDL-2
H2FC: Free Cholesterol HDL-2
H2PL: Phospholipids HDL-2
H2TG: Triglycerides HDL-2
H3A1: Apolipoprotein-A1 HDL-3
H3A2: Apolipoprotein-A2 HDL-3
H3CH: Cholesterol HDL-3
H3FC: Free Cholesterol HDL-3
H3PL: Phospholipids HDL-3
H3TG: Triglycerides HDL-3
H4A1: Apolipoprotein-A1 HDL-4
H4A2: Apolipoprotein-A2 HDL-4
H4CH: Cholesterol HDL-4
H4FC: Free Cholesterol HDL-4
H4PL: Phospholipids HDL-4
H4TG: Triglycerides HDL-4
HDA1: HDL-Apolipoprotein-A1
HDA2: HDL-Apolipoprotein-A2
HDCH: HDL-Cholesterol
HDFC: HDL Free Cholesterol
HDPL: HDL Phospholipids
HDTG: HDL Triglycerides
IDAB: IDL-Apolipoprotein-B100
IDCH: IDL Cholesterol
IDFC: IDL Free Cholesterol
IDPL: IDL Phospholipids
IDPN: ILDL Particle Number
IDTG: IDL Triglycerides
L1AB: Apolipoprotein-B100 LDL-1
L1CH: Cholesterol LDL-1
L1FC: Free Cholesterol LDL-1
L1PL: Phospholipids LDL-1
L1PN: Particle Number LDL-1
L1TG: Triglycerides LDL-1
L2AB: Apolipoprotein-B100 LDL-2
L2CH: Cholesterol LDL-2
L2FC: Free Cholesterol LDL-2
L2PL: Phospholipids LDL-2
L2PN: Particle Number LDL-2
L2TG: Triglycerides LDL-2
L3AB: Apolipoprotein-B100 LDL-3
L3CH: Cholesterol LDL-3
L3FC: Free Cholesterol LDL-3
L3PL: Phospholipids LDL-3
L3PN: Particle Number LDL-3
L3TG: Triglycerides LDL-3
L4AB: Apolipoprotein-B100 LDL-4
L4CH: Cholesterol LDL-4
L4FC: Free Cholesterol LDL-4
L4PL: Phospholipids LDL-4
L4PN: Particle Number LDL-4
L4TG: Triglycerides LDL-4
L5AB: Apolipoprotein-B100 LDL-5
L5CH: Cholesterol LDL-5
L5FC: Free Cholesterol LDL-5
L5PL: Phospholipids LDL-5
L5PN: Particle Number LDL-5
L5TG: Triglycerides LDL-5
L6AB: Apolipoprotein-B100 LDL-6
L6CH: Cholesterol LDL-6
L6FC: Free Cholesterol LDL-6
L6PL: Phospholipids LDL-6
L6PN: Particle Number LDL-6
L6TG: Triglycerides LDL-6
LDAB: LDL-Apolipoprotein-B100
LDCH: LDL-Cholesterol
LDFC: LDL Free Cholesterol
LDHD or LD/HD: LDL-cholesterol/HDL-cholesterol
LDPL: LDL Phospholipids
LDPN: LDL Particle Number
LDTG: LDL Triglycerides
TBPN: Total Particle Number (apolipoprotein-B100 carrying particles)
TPA1: Total Plasma Apolipoprotein-A1
TPA2: Total Plasma Apolipoprotein-A2
TPAB: Total Plasma Apolipoprotein-B100
TPCH: Total Plasma Cholesterol
TPTG: Total Plasma Triglycerides
V1CH: Cholesterol VLDL-1
V1FC: Free Cholesterol VLDL-1
V1PL: Phospholipids VLDL-1
V1TG: Triglycerides VLDL-1
V2CH: Cholesterol VLDL-2
V2FC: Free Cholesterol VLDL-2
V2PL: Phospholipids VLDL-2
V2TG: Triglycerides VLDL-2
V3CH: Cholesterol VLDL-3
V3FC: Free Cholesterol VLDL-3
V3PL: Phospholipids VLDL-3
V3TG: Triglycerides VLDL-3
V4CH: Cholesterol VLDL-4
V4FC: Free Cholesterol VLDL-4
V4PL: Phospholipids VLDL-4
V4TG: Triglycerides VLDL-4
V5CH: Cholesterol VLDL-5
V5FC: Free Cholesterol VLDL-5
V5PL: Phospholipids VLDL-5
V5TG: Triglycerides VLDL-5
VLAB: VLDL-Apolipoprotein-B100
VLCH: VLDL Cholesterol
VLFC: VLDL Free Cholesterol
VLPL: VLDL Phospholipids
VLPN: VLDL Particle Number
VLTG: VLDL Triglycerides
AC: Acute COVID-19
LC: Long COVID-19 or long term COVID-19 syndrome
IL: Interleukin
IFN: Interferon
TNF: Tumor necrosis factor
MCP-1, CCL2/MCP-1: Monocyte chemoattractant protein
INR: International normalized ratio
CRP: C-reactive protein
GFR CKD-EPI: Glomerular filtration rate using the formula from chronic kidney disease epidemiology collaboration
LDH: Lactate dehydrogenase
GOT: Glutamic-oxaloacetic transaminase
GPT: Glutamic-pyruvic transaminase
GGT: Gamma-glutamyl transferase
CXCL8/IL-8, IL-8: Interleukin-8
