# Supplementary material for: Longitudinal lipoprotein and inflammatory mediators analysis uncover persisting inflammation and hyperlipidemia following SARS-CoV-2 infection in long COVID-19
Source: Metabolomics. 2025 May 7;21(3):65. doi: 10.1007/s11306-025-02262-y (PMC12058914; doi:10.1007/s11306-025-02262-y)
Supplement: Supplementary file 1 — Supplementary Material 1 [file 11306_2025_2262_MOESM1_ESM.docx]

**SUPPLEMENTARY INFORMATION**

**Longitudinal lipoprotein and inflammatory mediators analysis uncover persisting inflammation and hyperlipidemia following SARS-CoV-2**

Gyuntae Bae ^1,2^, Zhiqi Yang ^3^, Daniele Bucci ^1^, Claire Wegner ^4^, Hartmut Schäfer ^4^, Yogesh Singh ^3,5,6^, Caterina Lonati ^7 *^, Christoph Trautwein ^1,2,8,9 *^

^1^ Werner Siemens Imaging Center, Department of Preclinical Imaging and Radiopharmacy, University Hospital Tübingen, Tübingen, Germany

^2^ Cluster of Excellence iFIT (EXC2180) 'Image-Guided and Functionally Instructed Tumor Therapies', University of Tübingen, Tübingen, Germany

^3^ Research Institute of Women’s Health, University of Tübingen, Tübingen, Germany

^4^ Bruker BioSpin GmbH & Co. KG, Biopharma & Applied Division, Ettlingen, Germany

^5^ Institute of Medical Genetics and Applied Genomics, University of Tübingen, Tübingen, Germany

^6^ Next Generation Sequencing (NGS) Competence Center Tübingen (NCCT), University of Tübingen, Tübingen, Germany

^7^ Center for Preclinical Research, Fondazione IRCCS Ca’ Granda Ospedale Maggiore Policlinico, Milan, Italy

^8^ M3 Research Center for Malignome, Metabolome and Microbiome, Faculty of Medicine University Tübingen, Tübingen, Germany

^9^ Core Facility Metabolomics, Medical Faculty University of Tübingen, University Tübingen, Tübingen, Germany

***Corresponding authors:**

caterina.lonati@unimi.it

christoph.trautwein@med.uni-tuebingen.de

**Running tittle:** Persistent inflammation and hyperlipidemia in Long COVID-19

**Supplementary table 1 –** **Clinical characteristics of long COVID-19 patients at month 5, 9, 12, 16, and 20.**

|  | 5M (N = 95) | 9M(N=73) | 12M(N=95) | 16M (N = 78) | 20M (N = 85) |
| --- | --- | --- | --- | --- | --- |
| Inpatient (Acute COVID-19 time point) | 34.34% | 35.61% | 32.63% | 32.05% | 31.76% |
| Asthma | 14.40% | 13.69% | 12.63% | 11.53% | 12.94% |
| Hypertension | 37.66% | 36.98% | 42.10% | 43.58% | 43.52% |
| Coronary heart disease | 4.43% | 4.10% | 5.26% | 3.84% | 5.88% |
| Dilated cardiomyopathy | 2.21% | 2.73% | 2.10% | 2.56% | 1.17% |
| Heart palpitations | 16.61% | 16.43% | 11.57% | 8.97% | 17.64% |
| Diabetes mellitus type 2 | 7.74% | 6.84% | 10.52% | 8.97% | 8.23% |
| Active malignancy | 4.43% | 4.10% | 4.21% | 5.12% | 7.05% |
| Autoimmune disease | 8.86% | 9.58% | 7.36% | 6.41% | 9.41% |
| Depression | 7.74% | 5.47% | 8.42% | 8.97% | 9.41% |
| Oxygen support category (Acute COVID-19 time point) |  |  |  |  |  |
| Supplemental oxygen via nasal tube | 23.26% | 23.28% | 22.10% | 23.07% | 22.35% |
| Noninvasive ventilation via high-flow nasal oxygen | 3.31% | 4.10% | 3.15% | 1.28% | 3.52% |
| Invasive ventilation | 4.43% | 4.10% | 4.21% | 5.12% | 3.52% |
| Disease severity (Acute COVID-19 time point) |  |  |  |  |  |
| Mild | 15.50% | 17.80% | 15.78% | 17.94% | 15.29% |
| Moderate | 58.71% | 50.68% | 54.73% | 52.56% | 55.29% |
| Mild/moderate, ≥60 y | 46.53% | 26.02% | 13.68% | 23.07% | 24.70% |
| Mild/moderate, male | 28.80% | 21.91% | 17.89% | 29.48% | 28.23% |
| Severe | 26.58% | 27.39% | 75.78% | 24.35% | 25.88% |
| Critical | 4.43% | 4.10% | 4.21% | 5.12% | 4.70% |
| Severe/critical, ≥60 y | 15.50% | 16.43% | 7.36% | 16.66% | 14.11% |
| Severe/critical, male | 18.83% | 17.80% | 17.89% | 17.94% | 17.64% |
| Vaccinated | 0% | 0% | 7.36% | 91.02% | 96.47% |
| Medication |  |  |  |  |  |
| Angiotensin-converting enzyme (ACE) inhibitors | 14.73% | 16.43% | 14.73% | 16.66% | 16.47% |
| AT1-antagonist | 14.73% | 16.43% | 13.68% | 15.38% | 17.64% |
| Beta (ß)-lactamase inhibitor | 16.84% | 19.17% | 15.78% | 17.94% | 17.64% |
| Calcium-antagonists | 8.42% | 9.58% | 8.42% | 10.25% | 9.41% |
| Acetylsalicylic acid | 8.42% | 8.21% | 8.42% | 5.12% | 9.41% |
| Statins | 16.84% | 19.17% | 17.89% | 15.38% | 17.64% |
| Citalopram | 2.10% | 1.36% | 2.10% | 2.56% | 2.35% |
| Antidepressants | 8.42% | 6.84% | 8.42% | 8.97% | 11.76% |
| Cortisone | 3.15% | 2.73% | 3.15% | 2.56% | 3.52% |

Supplementary table 1: Presence of clinical characteristics of long COVID-19 cohort at time point 5, 9, 12, 16, and 20 months are described in percentage.

**Supplementary table 2 – Clinical presentations of acute COVID-19 patients.**

|  | (N = 307, 170 patients) |
| --- | --- |
| Dyspnea 1-4 (mild to severe) |  |
| 1 | 44.29% |
| 2 | 21.82% |
| 3 | 17.58% |
| 4 | 6.84% |
| NA | 9.44% |
| Median of max breathing rate / minute (Min, Max) | 24 (20, 48) |
| NA | 14.65% |
| Hospitalized | 38.11% |
| NA | 4.88% |
| Decease | 1.95% |
| NA | 0.32% |
| Heart palpitations | 1.30% |
| NA | 2.28% |
| Diabetes mellitus type 2 | 12.70% |
| NA | 0.32% |
| Asthma | 11.07% |
| NA | 0.32% |
| Chronic obstructive pulmonary disease | 4.88% |
| NA | 0.32% |
| Obstructive sleep apnea syndrome | 10.42% |
| NA | 89.57% |
| Depression | 5.86% |
| NA | 0.32% |
| Active malignancy | 5.86% |
| NA | 0.32% |
| Rheumatoid arthritis | 2.60% |
| NA | 97.39% |
| Cardiac insufficiency | 2.28% |
| NA | 97.71% |
| Inflammatory bowel disease | 1.95% |
| NA | 98.04% |

Supplementary table 2: Presence of clinical presentations of acute COVID-19 cohort are described in percentage. NA indicates the information is not available.

**Supplementary table 3 – Description of lipoproteins and the biological role.**

| **Lipoproteins** | **Role** |
| --- | --- |
| TPTG | Total concentration of circulating triglycerides for cellular energy metabolism (Watt and Cheng, 2017). |
| TPCH | Total concentration of circulating cholesterols for maintenance of cellular membrane (Guo *et al.*, 2024). |
| VDL | Very-low density lipoprotein is produced by the liver for triglyceride uptake of the peripheral tissues and metabolized into IDL. |
| IDL | Intermediate density lipoprotein is carried to liver and metabolized into LDL (Feingold, 2022). |
| LDL | Low-density lipoprotein carries cholesterols to the liver and all peripheral tissues (Feingold, 2022). |
| HDL | High-density lipoprotein carries cholesterols to the liver (Feingold, 2022). |
| TPA1 | Total concentration of apolipoprotein A-I, which is a main component of HDL, facilitate in reverse transportation of cholesterol and homeostasis of cellular cholesterol (Rader *et al.*, 1991). |
| TPA2 | Total concentration of apolipoprotein A-II, second major component of HDL, is important for lipid metabolism (Florea *et al.*, 2022). |
| ABA1 or AB/A1 | It is the ratio of apolipoprotein B-100 over apolipoprotein A-I and known as a marker for balance of proinflammatory and anti-inflammatory state. |
| LDHD or LD/HD | It is LDL over HDL and used as an indicator of vascular risks. |

Supplementary table 3: lipoproteins that can be measured and quantified by in vitro diagnostics research (IVDr)-based nuclear magnetic resonance (NMR) spectroscopy at 600 MHz are described in terms of functions.

**Supplementary table 4 – Summary on roles of 13 cytokines.**

| **Mediator** | **Classification** | **Main Source(s)** | **Main functions** |
| --- | --- | --- | --- |
| IL-1β | Proinflammatory | Macrophages | Host-response and resistance to pathogens and specific to chronic disease and acute tissue injury (Anquetil *et al.*, 2017; Lopez-Castejon and Brough, 2011). |
| IFN- α2 | Proinflammatory | Macrophages, fibroblasts, endothelial cells, and plasmacytoid dendritic cells. | Host defense against viruses. Activation of monocytes, antigen-presenting cells, dendritic cells, macrophages, NK cells, T cells, and B cells (McNab *et al.*, 2015; Prchal *et al.*, 2009). |
| IFN-γ | Proinflammatory | T lymphocytes and natural killer cells | Macrophage activation, enhancement of antigen presentation, modulation of T-cells, and antiviral and antitumor activity (Alspach *et al.*, 2019). |
| TNF-α | Proinflammatory | Activated macrophages, T-lymphocytes, and natural killer cells | Apoptosis, necrosis, and regulation of cell signaling pathway (Horiuchi *et al.*, 2010; Idriss and Naismith, 2000) |
| CXCL8/IL-8 | Proinflammatory | Macrophages, endothelial cells, and epithelial cells | Neutrophil and T- Cells activation, monocyte-macrophage growth and differentiation (Corre *et al.*, 1999; David *et al.*, 2016). |
| IL-12p70 | Proinflammatory | Monocytes, macrophages and dendritic cells | Activation of natural killer cells and T lymphocytes (D'Andrea *et al.*, 1992; Liu *et al.*, 2005; Macatonia *et al.*, 1995). |
| IL-17A | Proinflammatory | Th17 cells | Induction of various proinflammatory cytokines TNF-α, IL-1β, and IL-6) (Chen *et al.*, 2013; Gaffen, 2009). |
| IL-23 | Proinflammatory | Activated macrophages and dendritic cells. | Induction of chronic autoimmune inflammation and IL-17 (McKenzie *et al.*, 2006; Tang *et al.*, 2012) |
| IL-33 | Proinflammatory | Endothelial, epithelial, hematopoietic dendritic, mast, and macrophages cells | Induction of allergic inflammation and regulation of type 2 immunity (Fock *et al.*, 2013; Hsu *et al.*, 2010; Schmitz *et al.*, 2005; Yi *et al.*, 2022) |
| MCP-1 or CCL2/MCP-1 | Proinflammatory | Macrophages, endothelial cells, and adipocytes | Chemo-attractants and differentiation of monocytes (Deshmane *et al.*, 2009; Singh *et al.*, 2021) |
| IL-6 | Proinflammatory | Macrophages and monocytes | B-cell maturation, T-cell differentiation, and host defense (Gabay, 2006; Kamimura *et al.*, 2003; Korn and Hiltensperger, 2021). |
| IL-18 | Proinflammatory | Macrophages and dendritic cells | Activation of Th1 cells, cytotoxic CD8+ T cells, and natural killer (Ihim *et al.*, 2022; Swain, 2001). |
| IL-10 | Anti-inflammatory | T-helper cells, monocytes, macrophages, and dendritic cells | Reduction of proinflammatory cytokines (Iyer and Cheng, 2012) |

Supplementary table 4: Functions and sources of pro and anti-inflammatory mediators are described.

**Supplementary table 5 – Limit of detection of each cytokine in serum samples.**

| Cytokines | Limit of detection (pg/ml) |
| --- | --- |
| IL-1β | 1.5 + 0.6 |
| IFN-α2 | 2.1 + 0.2 |
| IFN-γ | 1.3 + 1.0 |
| TNF-α | 0.9 + 0.8 |
| MCP-1 or CCL2/MCP-1 | 1.1 + 1.2 |
| IL-6 | 1.5 + 0.7 |
| IL-8 | 2.0 + 0.5 |
| IL-10 | 2.0 + 0.5 |
| IL-12p70 | 2.0 + 0.2 |
| IL17A | 0.5 + 0.1 |
| IL-18 | 2.0 + 0.5 |
| IL-23 | 1.8 + 0.1 |
| IL-33 | 4.4 + 1.5 |

Supplementary table 5: Limit of detection of each cytokine that can be measured and quantified in serum is described.

**Supplementary table 6 – Delta days of quartiles-based vaccinated long COVID-19 cohort.**

| Quartiles | Delta in days before and after vaccination |
| --- | --- |
| Vac_LC_1 | 1 ≥ x ≤ 40 (N = 40) |
| Vac_LC_2 | 41 ≥ x ≤ 80 (N = 43) |
| Vac_LC_3 | 81 ≥ x ≤ 174.8 (N = 37) |
| Vac_LC_4 | 175 ≥ x ≤ 303 (N = 40) |

Supplementary table 6: Quartile-based vaccinated long COVID-19 cohort shows delta of the time point between before and after vaccination.

**Supplementary table 7 – Pooled 95% confidence interval of each main lipoprotein in Masuda’s healthy cohort.**

| Main lipoprotein | Pooled mean (Pooled 95% CI Lower & Upper) |
| --- | --- |
| HDCH (mg/dL) | 58.37 (95% CI: 56.88, 59.86) |
| HDFC (mg/dL) | 13.29 (95% CI: 12.87, 13.71) |
| HDPL (mg/dL) | 83.11 (95% CI: 81.14, 85.08) |
| HDTG (mg/dL) | 10.15 (95% CI: 9.78, 10.52) |
| IDCH (mg/dL) | 10.65 (95% Cl: 10.02, 11.27) |
| IDFC (mg/dL) | 2.65 (95% CI: 2.47, 2.83) |
| IDPL (mg/dL) | 5.36 (95% CI: 5.03, 5.69) |
| IDPN (nmol/L) | 73.35 (95% CI: 69.42, 77.27) |
| IDTG (mg/dL) | 6.35 (95% Cl: 5.51, 7.2) |
| LDCH (mg/dL) | 116.4 (95% Cl: 113.46, 119.35) |
| LDFC (mg/dL) | 33.33 (95% CI: 32.56, 34.09) |
| LDPL (mg/dL) | 66.32 (95% CI: 64.88, 67.76) |
| LDPN (nmol/L) | 1305.88 (95% CI: 1274.8, 1336.97) |
| LDTG (mg/dL) | 16.58 (95% CI: 16.07, 17.1) |
| TAP2 (mg/dL) | 34.53 (95% CI: 33.93, 35.13) |
| TPA1 (mg/dL) | 154.72 (95% CI: 152.09, 157.36) |
| TPAB (mg/dL) | 83.43 (95% CI: 81.44, 85.41) |
| TPCH (mg/dL) | 205.14 (95% CI: 200.67, 209.62) |
| TPTG (mg/dL) | 104.64 (95% CI: 99.7, 109.59) |
| VLCH (mg/dL) | 15.05 (95% CI: 14.01, 16.08) |
| VLFC (mg/dL) | 7.12 (95% CI: 6.67, 7.56) |
| VLPL (mg/dL) | 16.2 (95% CI: 15.19, 17.21) |
| VLPN (nmol/L) | 112.03 (95% CI: 105.39, 118.67) |
| VLTG (mg/dL) | 50.73 (95% CI: 46.88, 54.58) |

Supplementary table 7: Each main lipoprotein of healthy cohort was measured and quantified by Masuda et al (Masuda *et al.*, 2023). we then calculated sex-merged 95% confidence interval of each main lipoprotein. N number of male and female is 154 and 114, respectively.

**Supplementary table 8 – Pooled 95% CI of each main lipoprotein in long COVID-19 cohort.**

| Main lipoprotein (N=426) | Mean (95% CI Lower & Upper) |
| --- | --- |
| HDCH (mg/dL) | 68.82 (95% CI: 67.19, 70.46) |
| HDFC (mg/dL) | 15.81 (95% CI: 15.37, 16.26) |
| HDPL (mg/dL) | 96.49 (95% CI: 94.43, 98.54) |
| HDTG (mg/dL) | 13.32 (95% CI: 12.89, 13.74) |
| IDCH (mg/dL) | 14.75 (95% CI: 13.96, 15.53) |
| IDFC (mg/dL) | 4.264 (95% CI: 4.04, 4.49) |
| IDPL (mg/dL) | 9.089 (95% CI: 8.658, 9.52) |
| IDPN (nmol/L) | 113.2 (95% CI: 108.5, 117.9) |
| IDTG (mg/dL) | 14.85 (95% CI: 13.49, 16.21) |
| LDCH (mg/dL) | 123.1 (95% CI: 119.6, 126.7) |
| LDFC (mg/dL) | 36.59 (95% CI: 35.62, 37.57) |
| LDPL (mg/dL) | 70.15 (95% CI: 68.46, 71.83) |
| LDPN (nmol/L) | 1426 (95% CI: 1390, 1463) |
| LDTG (mg/dL) | 22.48 (95% CI: 21.83, 23.14) |
| TPA1 (mg/dL) | 175.5 (95% CI: 172.5, 178.6) |
| TPA2 (mg/dL) | 38.25 (95% CI: 37.56, 38.94) |
| TPAB (mg/dL) | 94.12 (95% CI: 91.96, 96.29) |
| TPCH (mg/dL) | 228 (95% CI: 223.7, 232.4) |
| TPTG (mg/dL) | 137.5 (95% CI: 129.5, 145.4) |
| VLCH (mg/dL) | 21.21 (95% CI: 19.76, 22.65) |
| VLFC (mg/dL) | 9.593 (95% CI: 9.04, 10.15) |
| VLPL (mg/dL) | 21.81 (95% CI: 20.62, 23) |
| VLPN (nmol/L) | 156.8 (95% CI: 148.5, 165.2) |
| VLTG (mg/dL) | 85.24 (95% CI: 79.55, 90.92) |

Supplementary table 8: We calculated sex-merged 95% confidence interval of each main lipoprotein in long COVID-19 cohort.

**Supplementary table 9 – Mean and standard deviation of the raw concentration of 13 cytokines in acute and long COVID-19 cohorts.**

| Cytokines | Acute (pg/ml) | Long 5 M (pg/ml) | Long 9 M (pg/ml) | Long 12 M (pg/ml) | |
| --- | --- | --- | --- | --- | --- |
| IL-1β | 2.73 ± 9.02 | 4.85 ± 12.56 | 2.34 ± 6.75 | 9.06 ± 16.66 | |
| IFN-α2 | 16.49 ± 28.86 | 19.46 ± 62.34 | 8.72 ± 8.46 | 26.59 ± 97 | |
| IFN-γ | 16.25 ± 33.23 | 9.97 ± 25.65 | 5.67 ± 10.8 | 19.59 ± 29.18 | |
| TNF-α | 7.26 ± 20.57 | 21.97 ± 146.42 | 4.03 ± 9.27 | 29.36 ± 165.69 | |
| MCP-1 | 475.94 ± 438.75 | 394.22 ± 187.85 | 279.71 ± 138.46 | 229.51 ± 127.73 | |
| IL-6 | 45.73 ± 105.61 | 19.24 ± 67.27 | 7.61 ± 10.87 | 20.82 ± 44.58 | |
| IL-8 | 68.44 ± 575.12 | 53.63 ± 306.59 | 9.51 ± 24.51 | 68.26 ± 314.2 | |
| IL-10 | 18.65 ± 42.99 | 13.93 ± 78.06 | 4.43 ± 8.4 | 18.83 ± 84.39 | |
| IL-12p70 | 3.56 ± 8.07 | 11.95 ± 41.72 | 5.4 ± 7.68 | 15.93 ± 44.05 | |
| IL-17A | 0.19 ± 0.23 | 1.64 ± 3.11 | 0.72 ± 1.17 | 2.39 ± 3.3 | |
| IL-18 | 1357.8 ± 2739.06 | 305.51 ± 167.04 | 219.65 ± 128.44 | 310.03 ± 207.07 | |
| IL-23 | 14.76 ± 53.67 | 23 ± 52.03 | 11.89 ± 17.34 | 50.85 ± 125.14 | |
| IL-33 | 64.73 ± 316.49 | 23.39 ± 52.48 | 12.01 ± 15.86 | 32.73 ± 82.86 | |
|  |  |  |  |  |  |
| Cytokines | **Long 16 M (pg/ml)** | **Long 20 M (pg/ml)** | **12 + 16 + 20 M (pg/ml)** | |  |
| IL-1β | 9.36 ± 15.84 | 12.96 ± 29.86 | 10.43 ± 21.71 | |  |
| IFN-α2 | 23.2 ± 41.31 | 35.02 ± 123.27 | 28.34 ± 94.57 | |  |
| IFN- γ | 18.02 ± 28.88 | 24.86 ± 69.32 | 20.85 ± 46.26 | |  |
| TNF-α | 24.58 ± 109.42 | 50.25 ± 339.93 | 34.8 ± 226.97 | |  |
| MCP-1 | 439.93 ± 232.01 | 523.55 ± 275.12 | 390 ± 251.02 | |  |
| IL-6 | 21.02 ± 43.91 | 37.61 ± 151.2 | 26.41 ± 94.01 | |  |
| IL-8 | 64.74 ± 244.9 | 116.44 ± 616.26 | 83.07 ± 422.8 | |  |
| IL-10 | 15.71 ± 54.48 | 32.98 ± 193.77 | 22.55 ± 125.78 | |  |
| IL-12p70 | 15.61 ± 32.7 | 26.01 ± 106.41 | 19.15 ± 68.95 | |  |
| IL-17A | 2.17 ± 2.52 | 2.92 ± 7.14 | 2.5 ± 4.76 | |  |
| IL-18 | 397.24 ± 211.4 | 507.87 ± 355.56 | 401.57 ± 277.91 | |  |
| IL-23 | 36.96 ± 46.91 | 45.7 ± 113.49 | 44.96 ± 103.1 | |  |
| IL-33 | 32.23 ± 45.66 | 45.11 ± 94.24 | 36.66 ± 77.94 | |  |

Supplementary table 9: Concentration of each cytokine in acute and long COVID-19 cohort is described. N number of acute and long COVID-19 cohort is 307 and 426 (Long COVID-19_5M_N = 95, Long COVID-19_9M_N = 73, Long COVID-19_12M_N = 95, Long COVID-19_16M_ N= 78, Long COVID-19_20M_ N = 85), respectively. Another name of MCP-1 is CCL2/MCP-1.

**Supplementary table 10 – Correlation of age with reduced exercise capacity and ANA_1:80 in each cytokine rank group.**

| **MCP-1** | Reduced_Exercise_Capacity (p-value) | | ANA_1:80 (p-value) |
| --- | --- | --- | --- |
| 1 | 0.49 | | 0.06 |
| 2 | 0.87 | | 0.87 |
| 3 | 0.2 | | 0.32 |
| 4 | 0.21 | | 0.88 |
| **IL-6** | Reduced_Exercise_Capacity | | ANA_1:80 |
| 1 | 0.56 | | **0.03 (r = 0.22)** |
| 2 | 0.2 | | 0.5 |
| 3 | 0.74 | | 0.7 |
| 4 | 0.9 | | 0.7 |
| **IL-18** | Reduced_Exercise_Capacity | ANA_1:80 | |
| 1 | 0.45 | 0.53 | |
| 2 | 0.08 | 0.67 | |
| 3 | 0.18 | **0.02(r = -0.23)** | |
| 4 | 0.81 | 0.47 | |
| **IL-23** | Reduced_Exercise_Capacity | ANA_1:80 | |
| 1 | 0.9 | 0.24 | |
| 2 | 0.37 | 0.82 | |
| 3 | 0.18 | 0.45 | |
| 4 | 0.13 | 0.65 | |

Supplementary table 10: Correlation of age and ANA_1:80 with each rank of proinflammatory mediators is calculated. Another name of MCP-1 is CCL2/MCP-1.

**Supplementary table 11 – Raw concentration of each cytokine based on rank in acute and long COVID-19 cohorts.**

| Acute (pg/ml) | | | | Long (pg/ml) | | | |
| --- | --- | --- | --- | --- | --- | --- | --- |
| MCP-1 | Min | Median | Max | **MCP-1** | Min | Median | Max |
| 1 | 0 | 108.81 | 151.43 | 1 | 0 | 182.3 | 273.9 |
| 2 | 161.72 | 251.46 | 321.42 | 2 | 275.47 | 317.16 | 367.84 |
| 3 | 323.18 | 416.71 | 548.72 | 3 | 367.89 | 429.15 | 512.34 |
| 4 | 550.74 | 745.83 | 4895.26 | 4 | 513.65 | 651.03 | 1467.51 |
| IL-6 |  |  |  | **IL-6** |  |  |  |
| 1 | 0 | 0.09 | 1.44 | 1 | 0 | 0 | 2.19 |
| 2 | 1.54 | 6.08 | 11.03 | 2 | 2.38 | 5.25 | 9.12 |
| 3 | 11.19 | 19.65 | 37.74 | 3 | 9.3 | 14.33 | 21.1 |
| 4 | 37.91 | 75.95 | 1308.9 | 4 | 21.19 | 30.08 | 1401.68 |
| IL-18 |  |  |  | **IL-18** |  |  |  |
| 1 | 0 | 241.16 | 417.37 | 1 | 0 | 142.08 | 185.89 |
| 2 | 421.26 | 560.65 | 742.38 | 2 | 186.13 | 237.11 | 278.66 |
| 3 | 745.78 | 1012.29 | 1387.5 | 3 | 283.55 | 348.01 | 438.44 |
| 4 | 1392.35 | 2160.27 | 30565.4 | 4 | 438.91 | 592.13 | 1787.93 |
| IL-23 |  |  |  | **IL-23** |  |  |  |
| 1 | 0 | 0 | 1.67 | 1 | 0 | 0.01 | 1.86 |
| 2 | 1.82 | 3.6 | 7.07 | 2 | 2.03 | 6.3 | 11.66 |
| 3 | 7.15 | 10.33 | 19.76 | 3 | 11.86 | 18.91 | 34.7 |
| 4 | 20.29 | 44.74 | 814.19 | 4 | 34.81 | 57.01 | 1181.86 |

Supplementary table 11: Raw concentration of the respective proinflammatory mediator rank-based group is described. Another name of MCP-1 is CCL2/MCP-1.


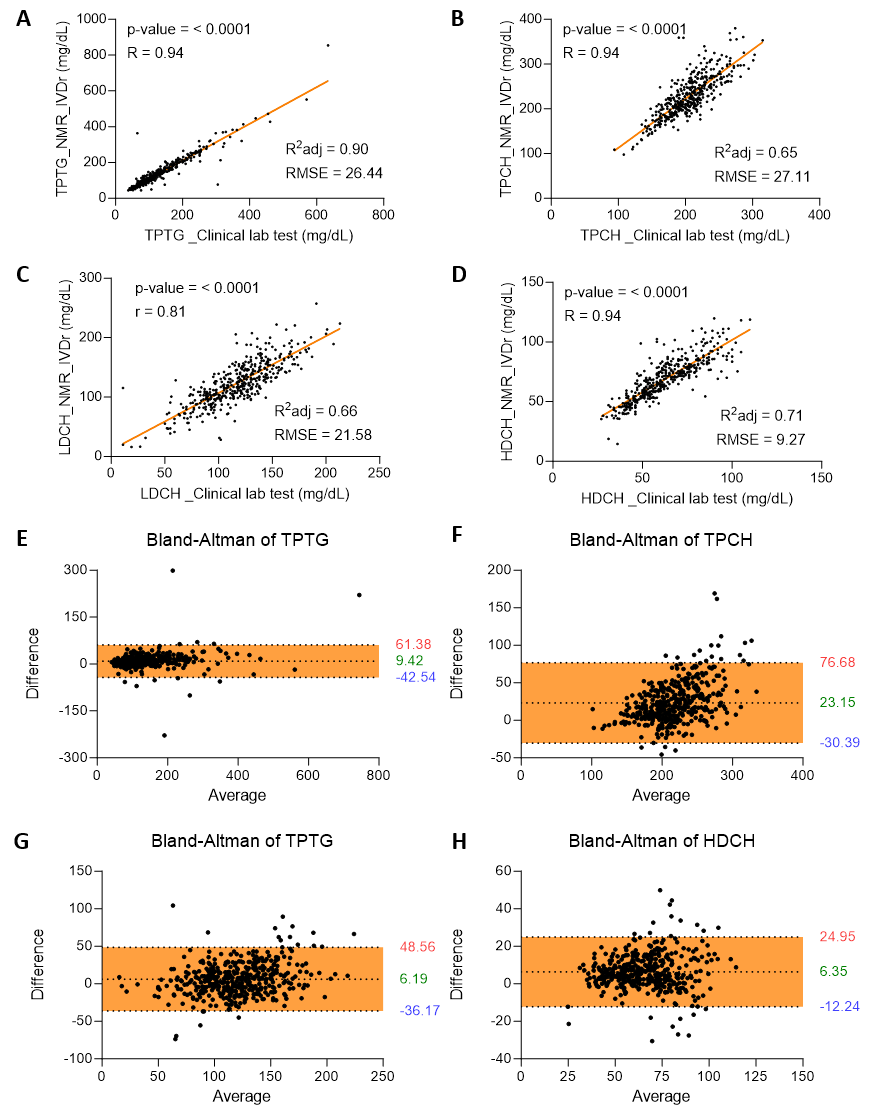


**Supplementary figure 1 – Agreement between NMR IVDr and clinical routine test.**

A) Result of Spearman correlation between TPTG that was measured and quantified by NMR IVDr and clinical routine test. B, C, D) Pearson correlation between TPCH, LDCH, and HDCH that were measured and quantified by NMR IVDr and clinical routine test. A, B, C, and D) X-axis represents the lipoproteins that were measured in a clinical routine test, while Y-axis indicates the lipoproteins that were measured with NMR IVDr. E, F, G and H) Bland-Altman plots of each lipoprotein that was measured by NMR IVDr and clinical routine test. E, F, G and H) X-axis represents average of each lipoprotein measured by the two different methods, while Y-axis shows the difference of each lipoprotein measured by the two different methods. E, F, G and H) Red and blue numbers indicate the 95% confidence interval upper and lower limit of the respective lipoprotein, respectively (limit of agreement) while green number represents the bias of the respective lipoprotein.


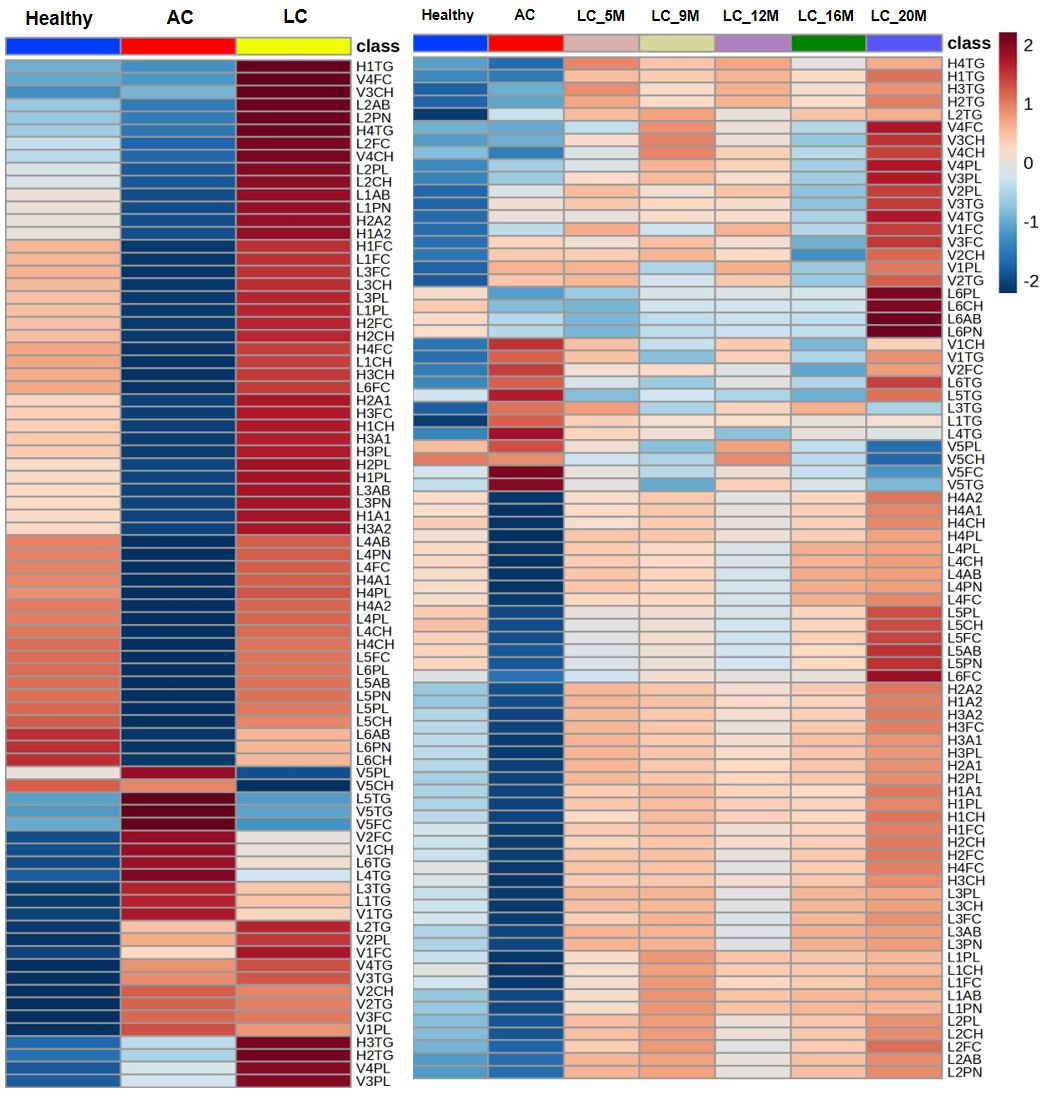


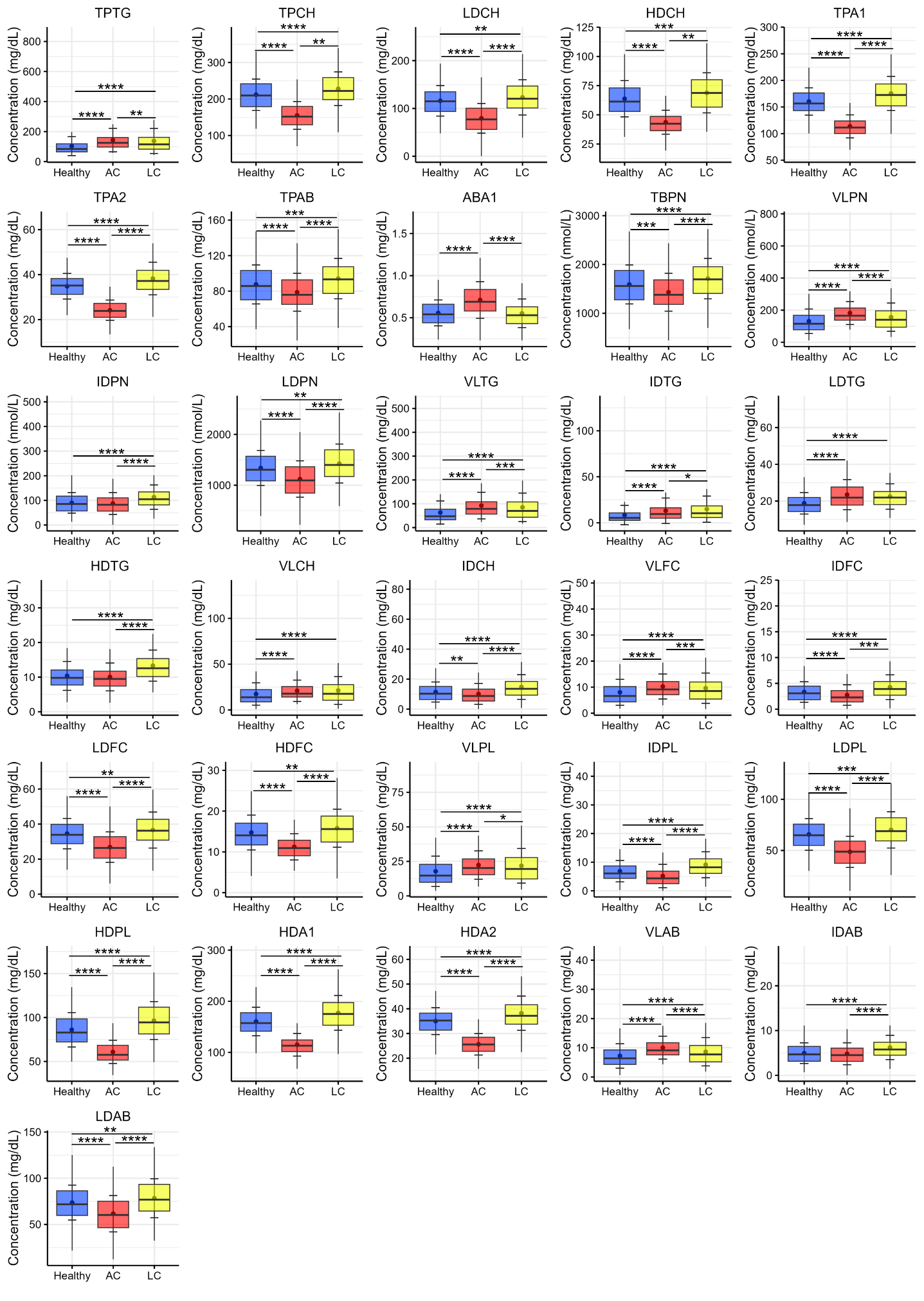


**
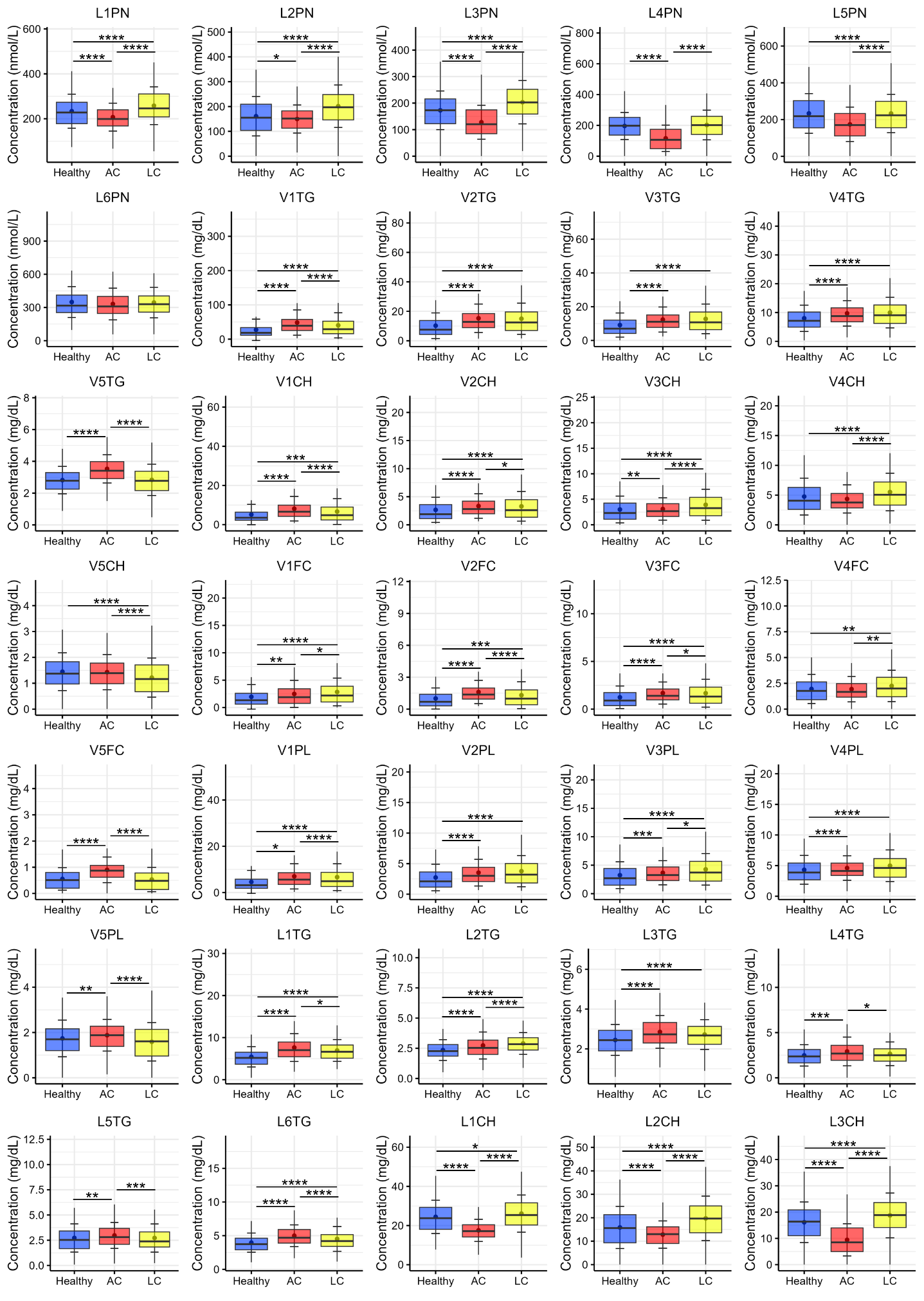
**

**
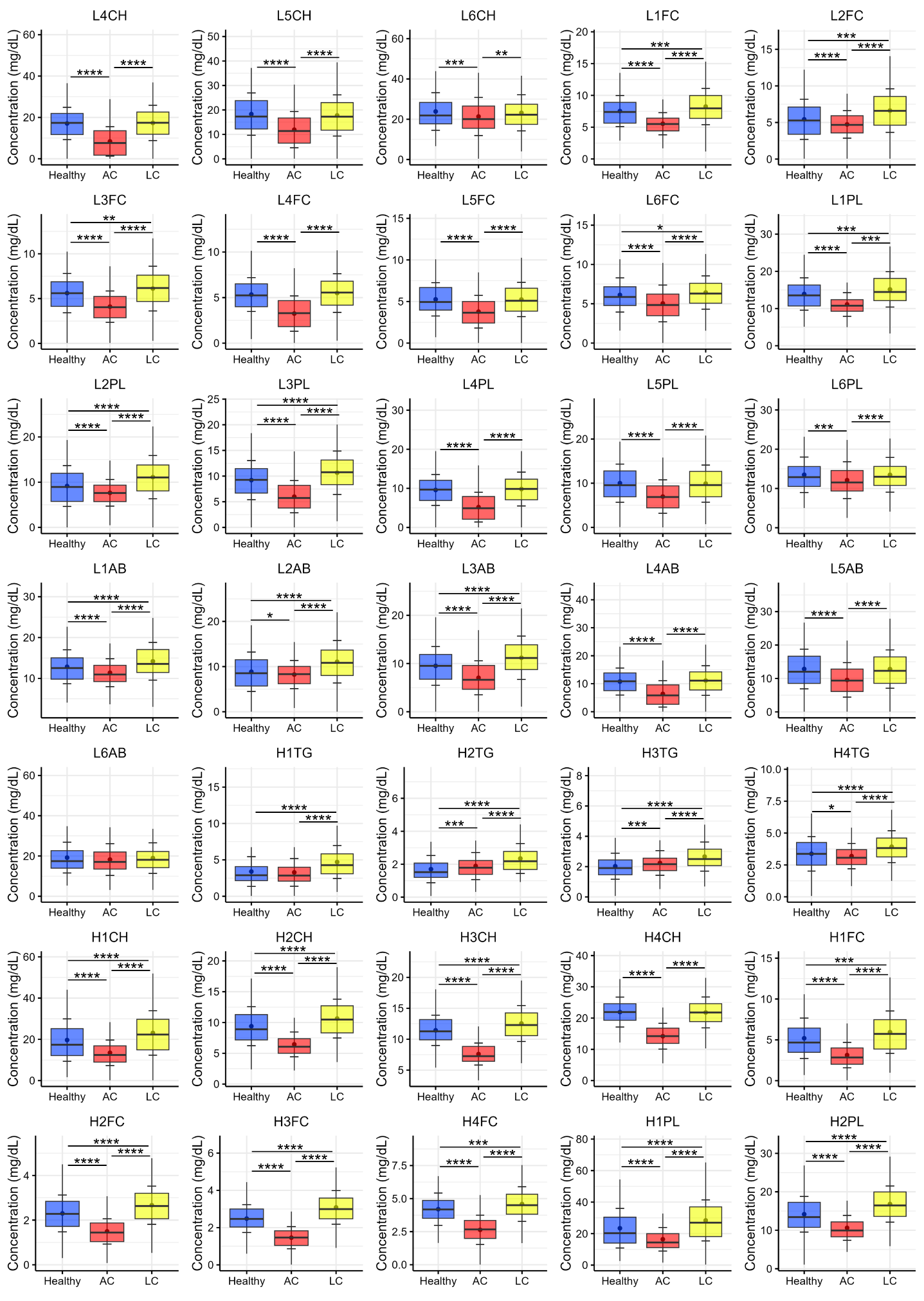
**

**
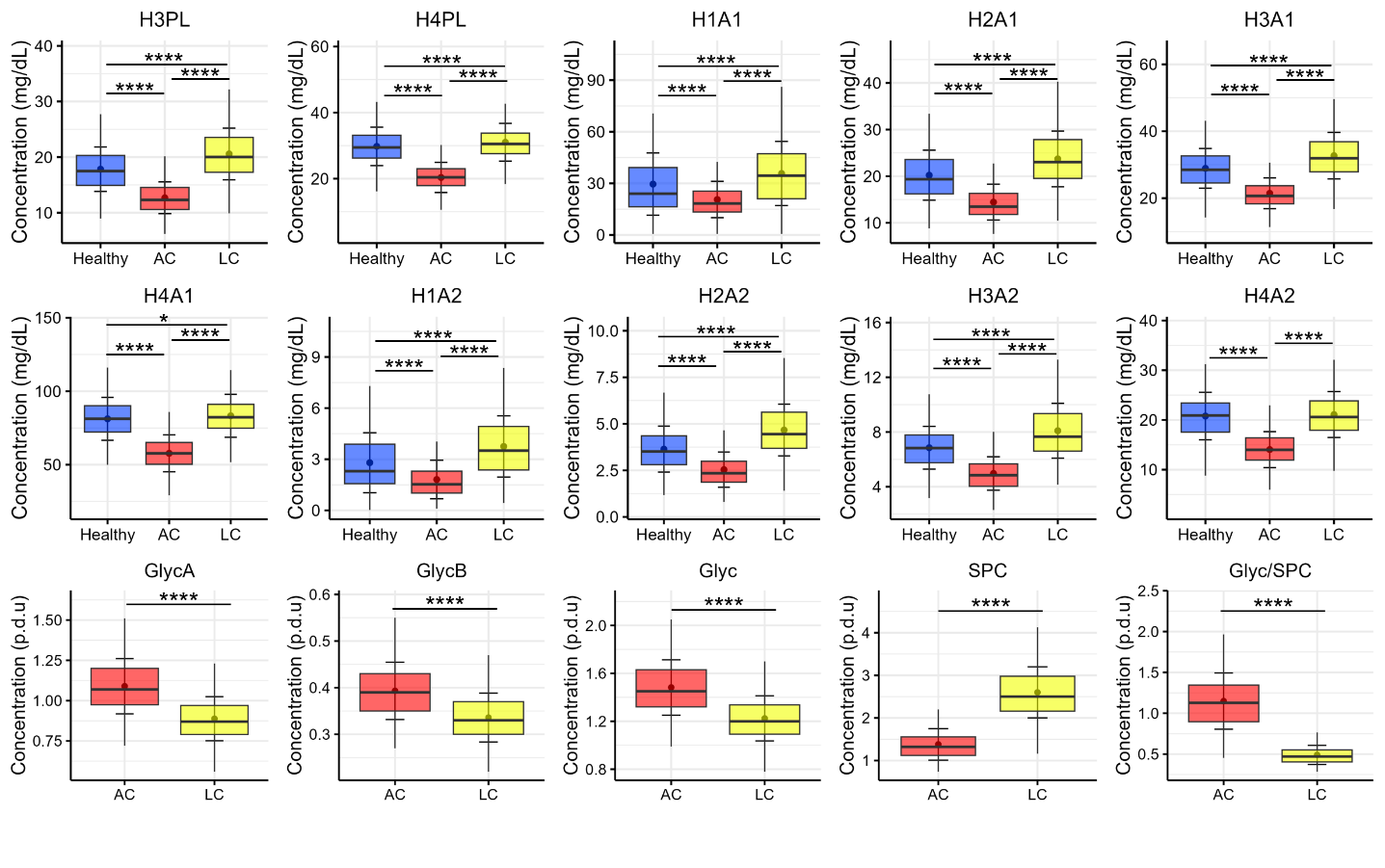
**

**Supplementary figure 2 – Hyperlipidemic phenotype with decreased acute inflammation in long COVID-19 cohort.**

A) Both left and right heatmaps shows 80 significantly altered main NMR lipoproteins (adjusted p-value < 0.05 by Ordinary ANOVA, Kruskal Wallis, and Brown Forsythe & Welch ANOVA tests). Heatmap (left) is the comparison between healthy, acute COVID-19, and long COVID-19 (5, 9, 12, 16, and 20 months merged). The average concentration of each lipoprotein parameter is displayed based on the color scale. A and B) Healthy (N = 305), Acute COVID-19 (N = 307), and long COVID-19 (LC) (N = 426) involves the following subsets: 5 M (N = 95); 9 M (N = 73);12 M (N = 95); 16 M (N = 78); 20 M (N = 85). All NMR parameters were significantly different between acute and long COVID-19 cohorts except L6PN and L6AB (adjusted p-value < 0.05 by Ordinary ANOVA, Kruskal Wallis, and Brown Forsythe & Welch ANOVA tests and post hoc test of the statistics: q-value = *<0.05, **< 0.01, ***<0.001, ****<0.0001). The straight line (inside the boxplot) and dot represent median and average, respectively.


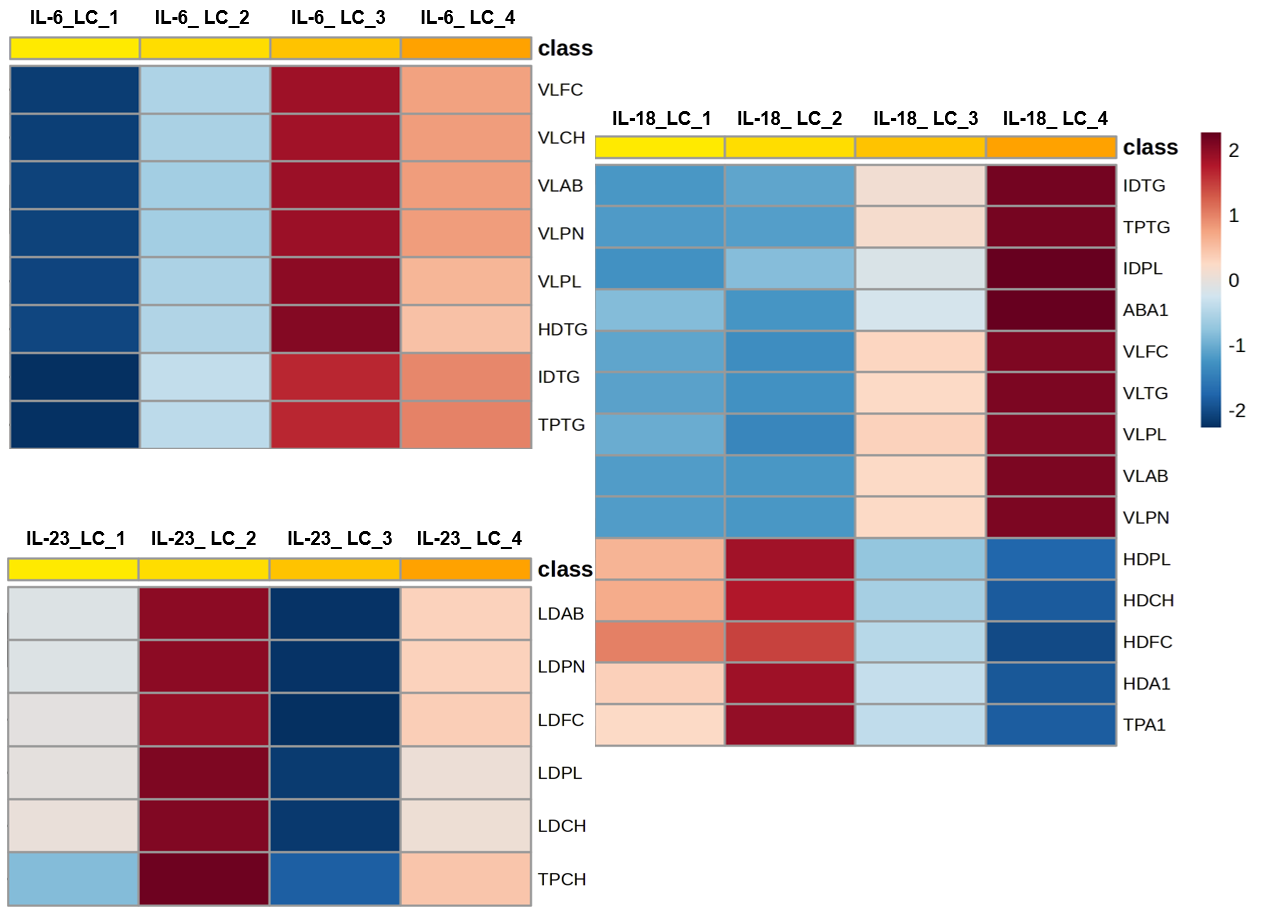


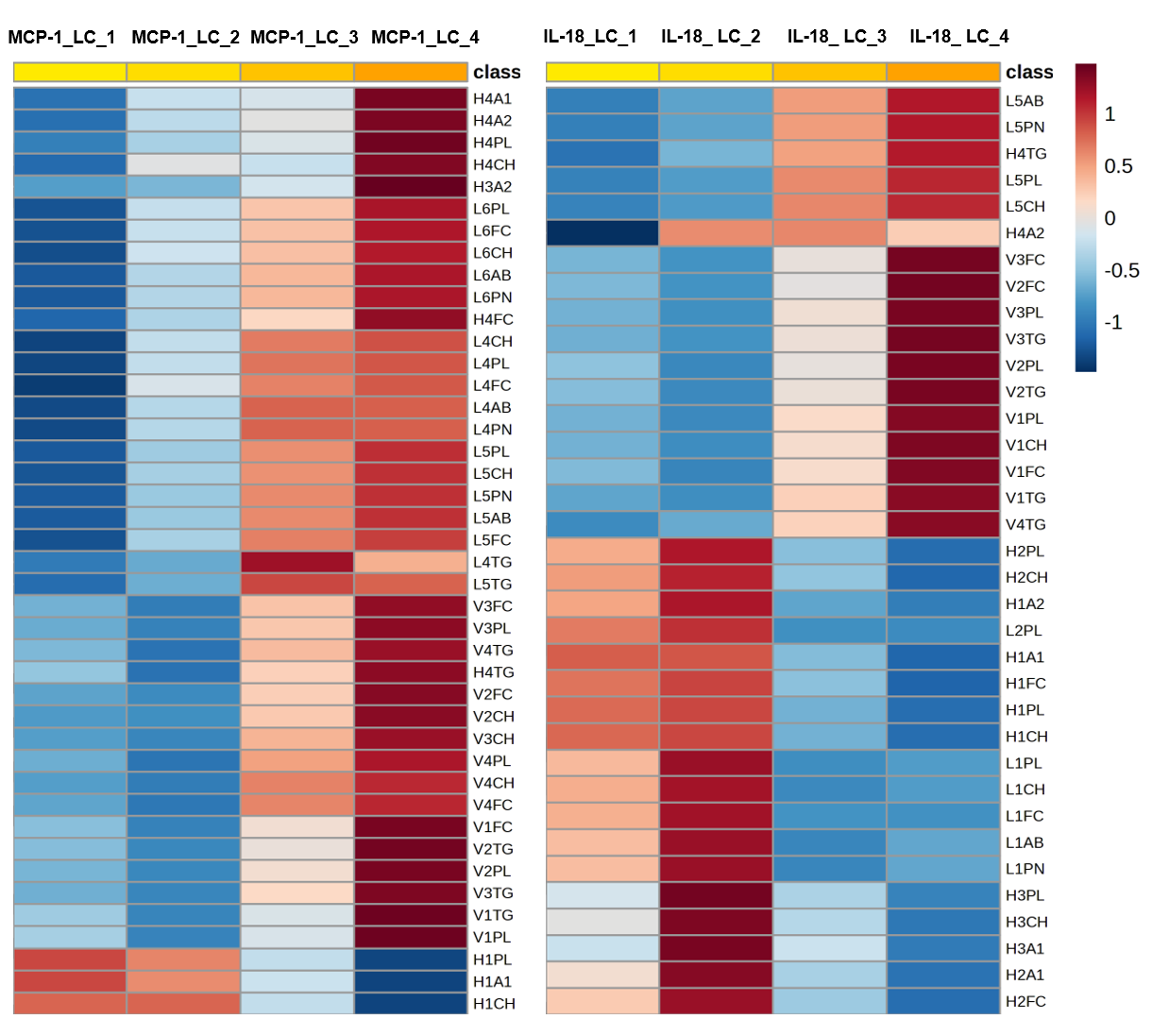


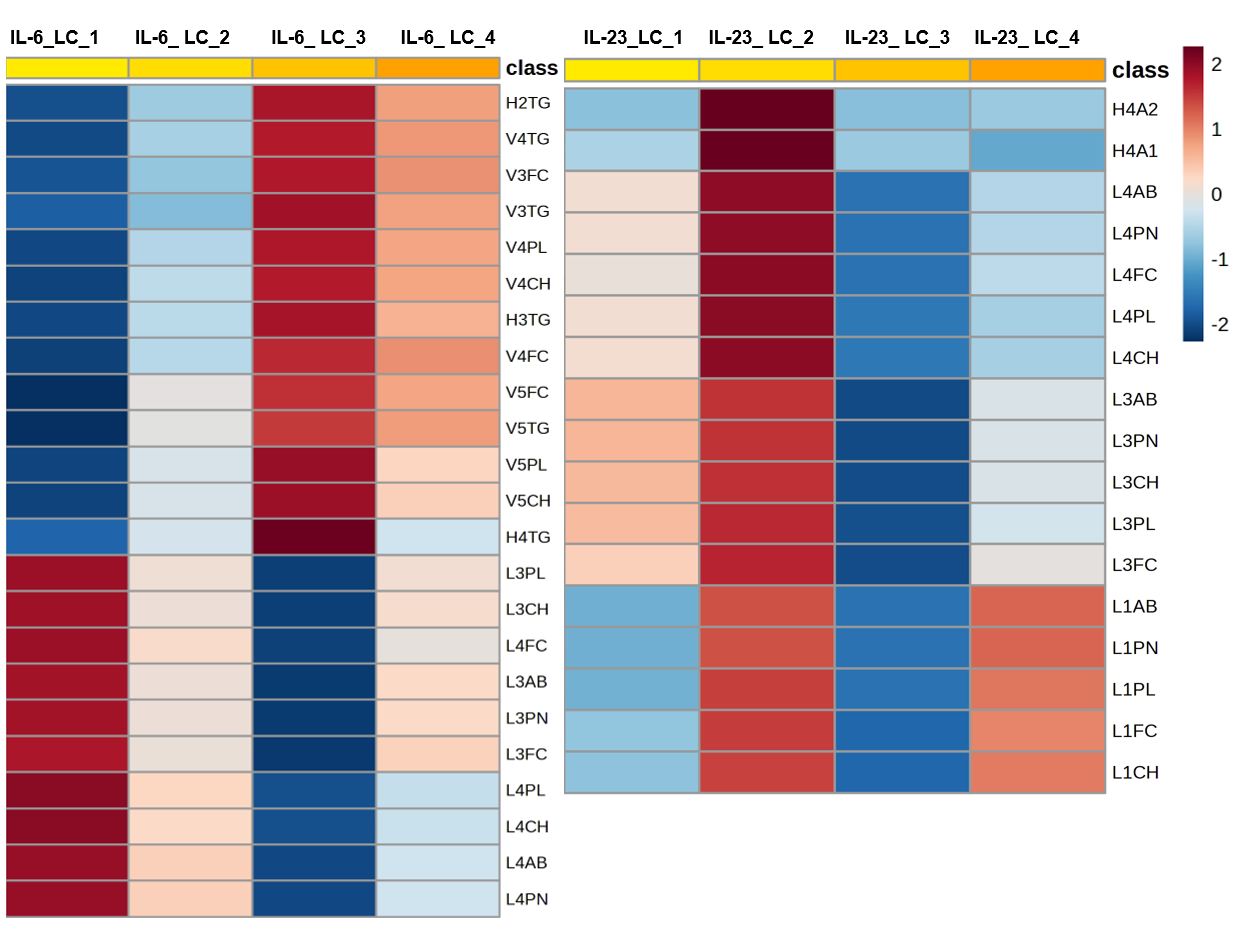


**Supplementary figure 3 – Re-stratified long COVID-19 cohort based on the rank of each cytokine.**

Heatmaps shows significantly altered main and subfraction of NMR lipoprotein (adjusted p-value < 0.05 by Ordinary ANOVA, Kruskal Wallis, and Brown Forsythe & Welch ANOVA tests) The average concentration of each lipoprotein parameter is displayed based on the color scale. Another name of MCP-1 is CCL2/MCP-1.


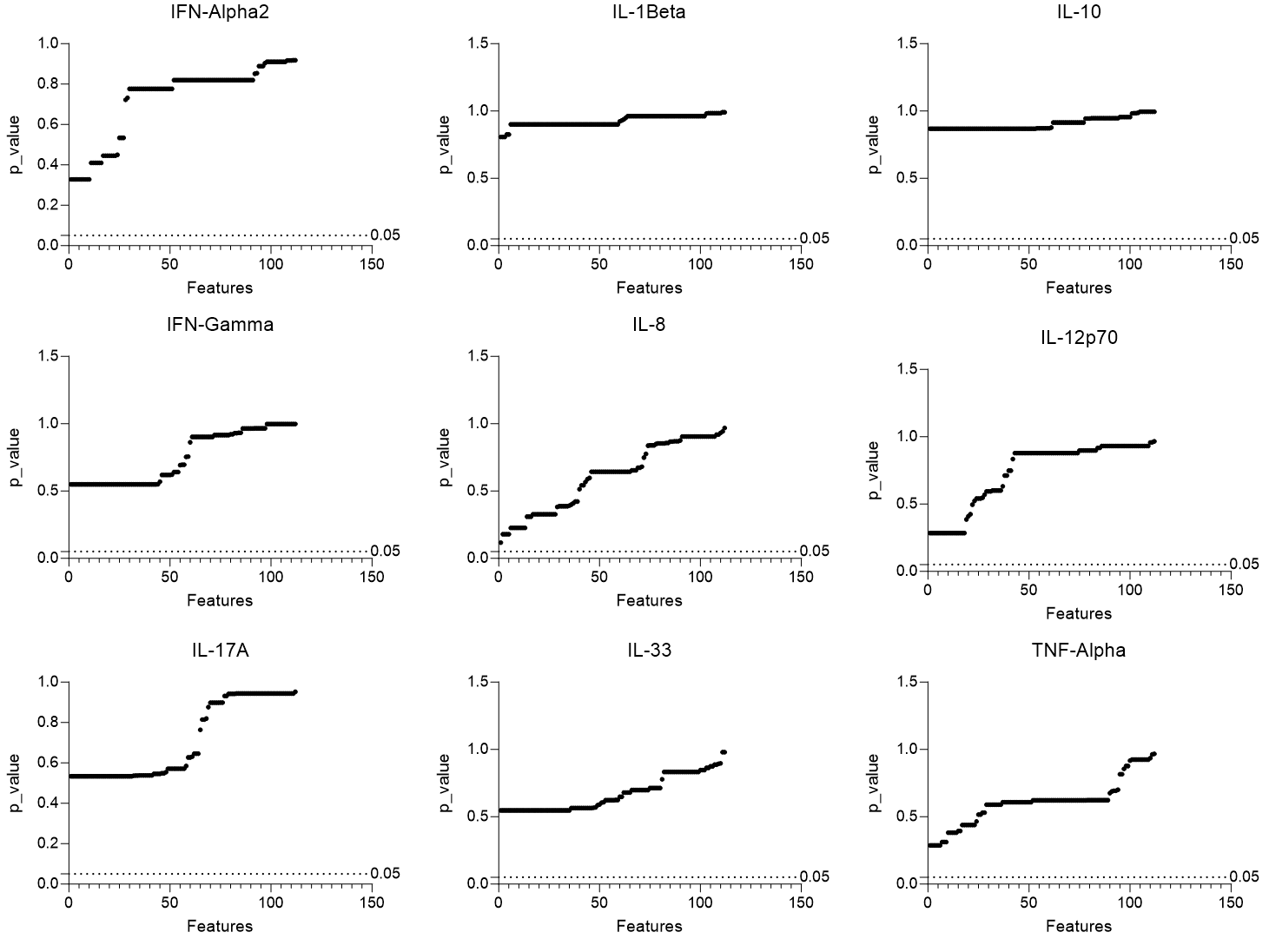


**Supplementary figure 4 – Long COVID-19 cohort based on the rank of the other cytokines.**

Plots were generated to show the rest of the rank of the cytokine failed to achieve refined stratification of long COVID-19 cohort. Another name of IL-8 is CXCL8/IL-8.


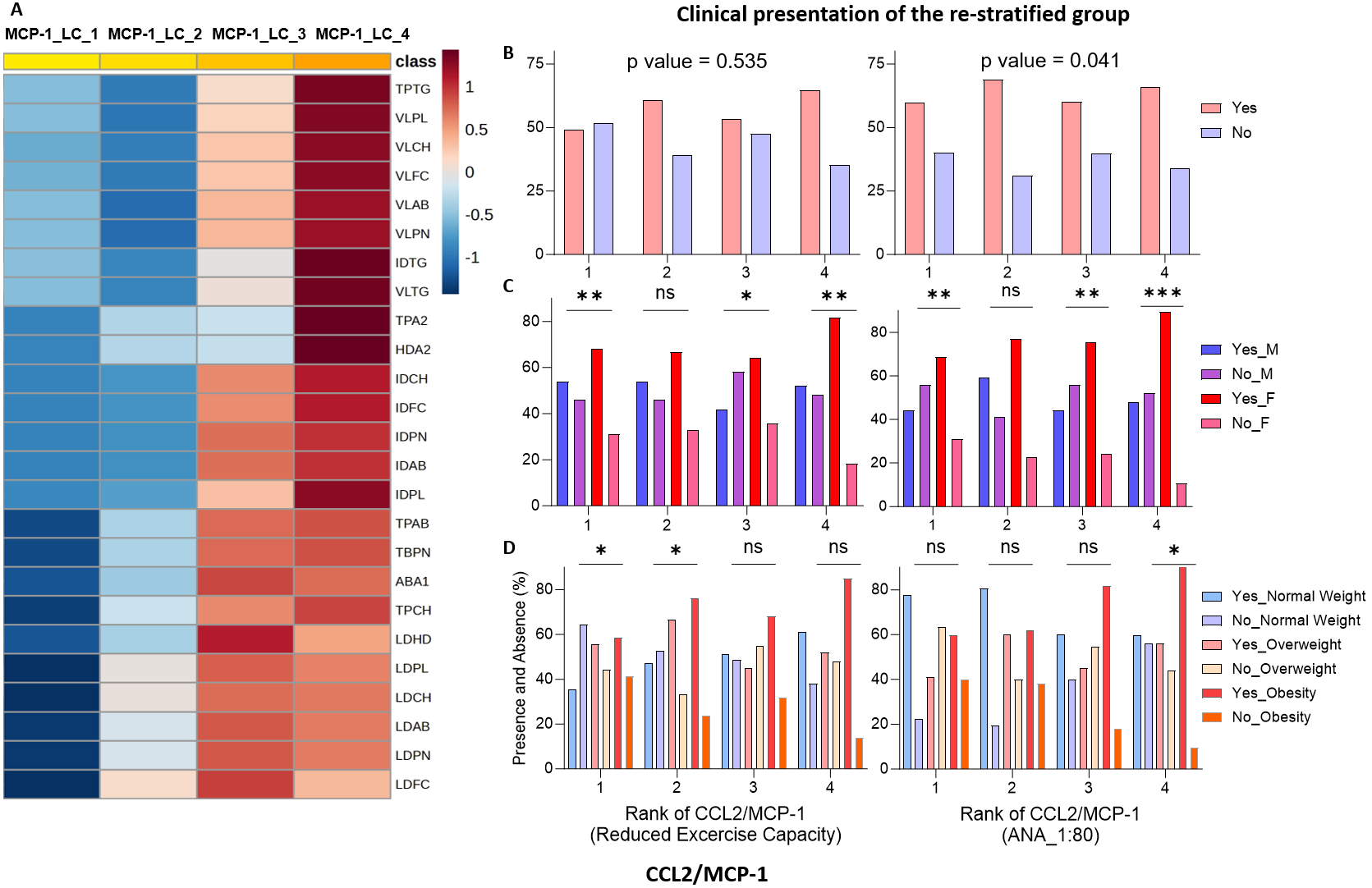
**Supplementary figure 5 – Re-stratified long COVID-19 group based on rank of the MCP-1 concentration.**

A) Heatmap shows 25 significantly altered main NMR lipoprotein parameters between the re-stratified long COVID-19 cohort, which we referred to a MCP-1 level in healthy cohort (de Lemos *et al.*, 2003) (adjusted p-value < 0.05 by one-way ANOVA; Kruskal Wallis and Brown Forsythe & Welch ANOVA tests). The Average concentration of each lipoprotein parameter is displayed based on the color scale. B-D) Linear by linear association test was implemented (p-value = NS >0.05, *<0.05, **< 0.01, ***<0.001). B) The Association of ANA_1:80 and reduced exercise capacity with the rank of MCP-1. C) Sex association with ANA_1:80 and reduced exercise capacity in each rank group. D) BMI association with ANA_1:80 and reduced exercise capacity in each rank group.


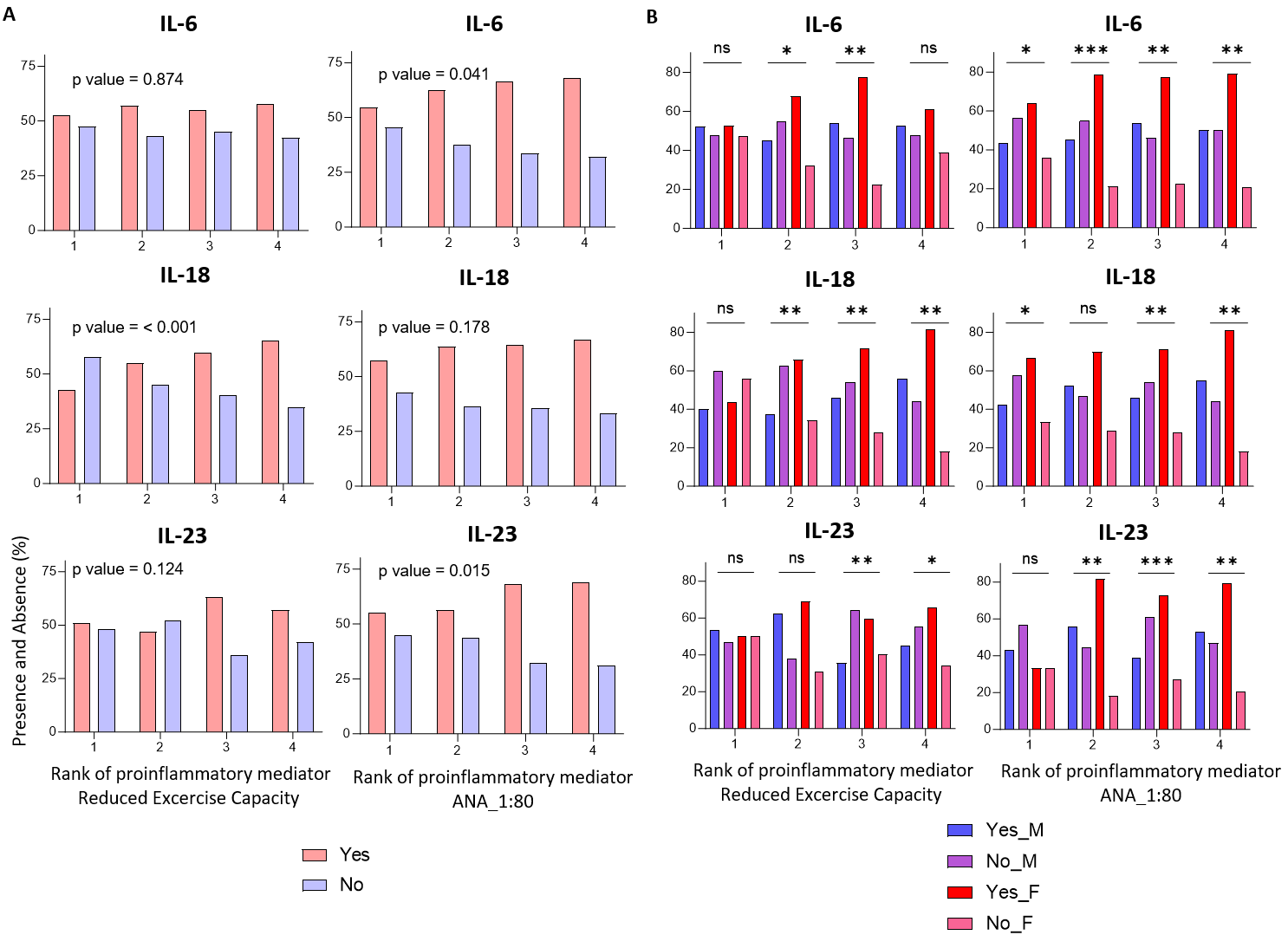


**Supplementary figure 6 – Association of reduced exercise capacity and ANA_1:80 with the rank of each cytokine based on sex.**

A & B) Linear by linear association test was implemented (p-value = NS >0.05, *<0.05, **< 0.01, ***<0.001, ****<0.0001). A) Association of ANA_1:80 and reduced exercise capacity with the rank of IL-6, IL-18, and IL-23. B) Sex association with ANA_1:80 and reduced exercise capacity in each rank group.


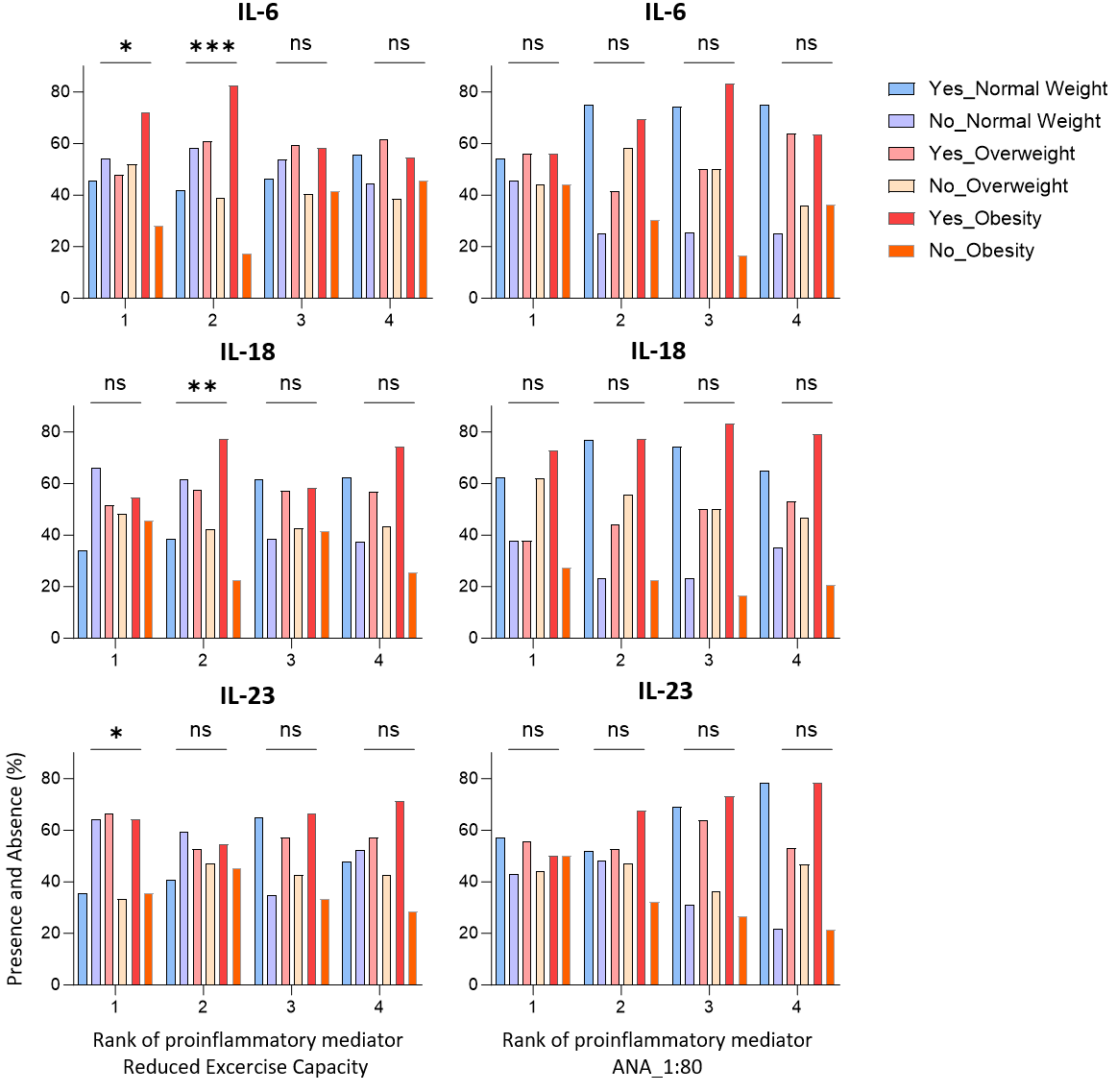


**Supplementary figure 7 – Association of reduced exercise capacity and ANA_1:80 with BMI in rank of each cytokine-based re-stratified group.**

Linear by linear association test was implemented (p-value = NS >0.05, *<0.05, **< 0.01, ***<0.001, ****<0.0001). Association of ANA_1:80 and reduced exercise capacity with BMI in each rank of IL-6, IL-18, and IL-23.


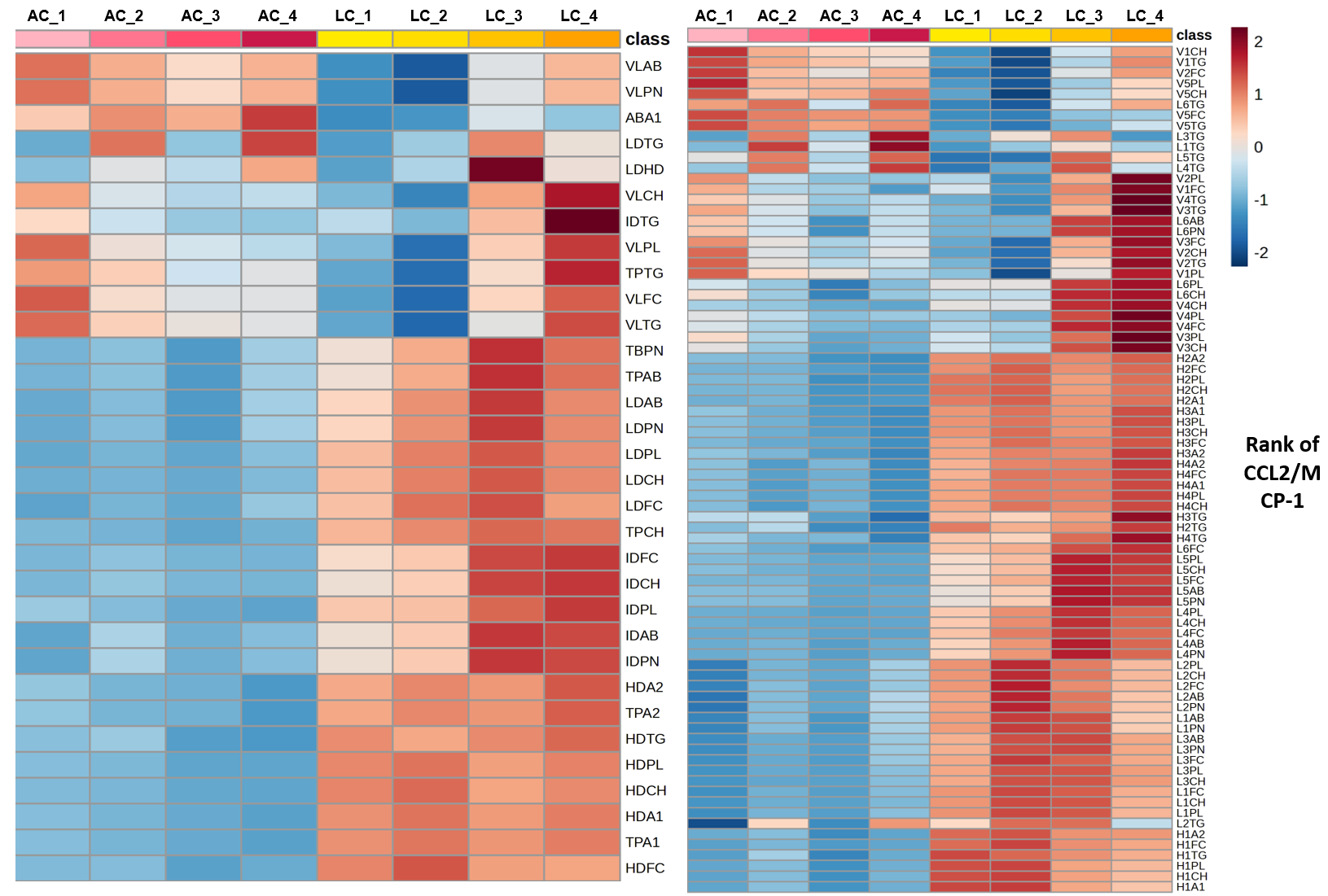


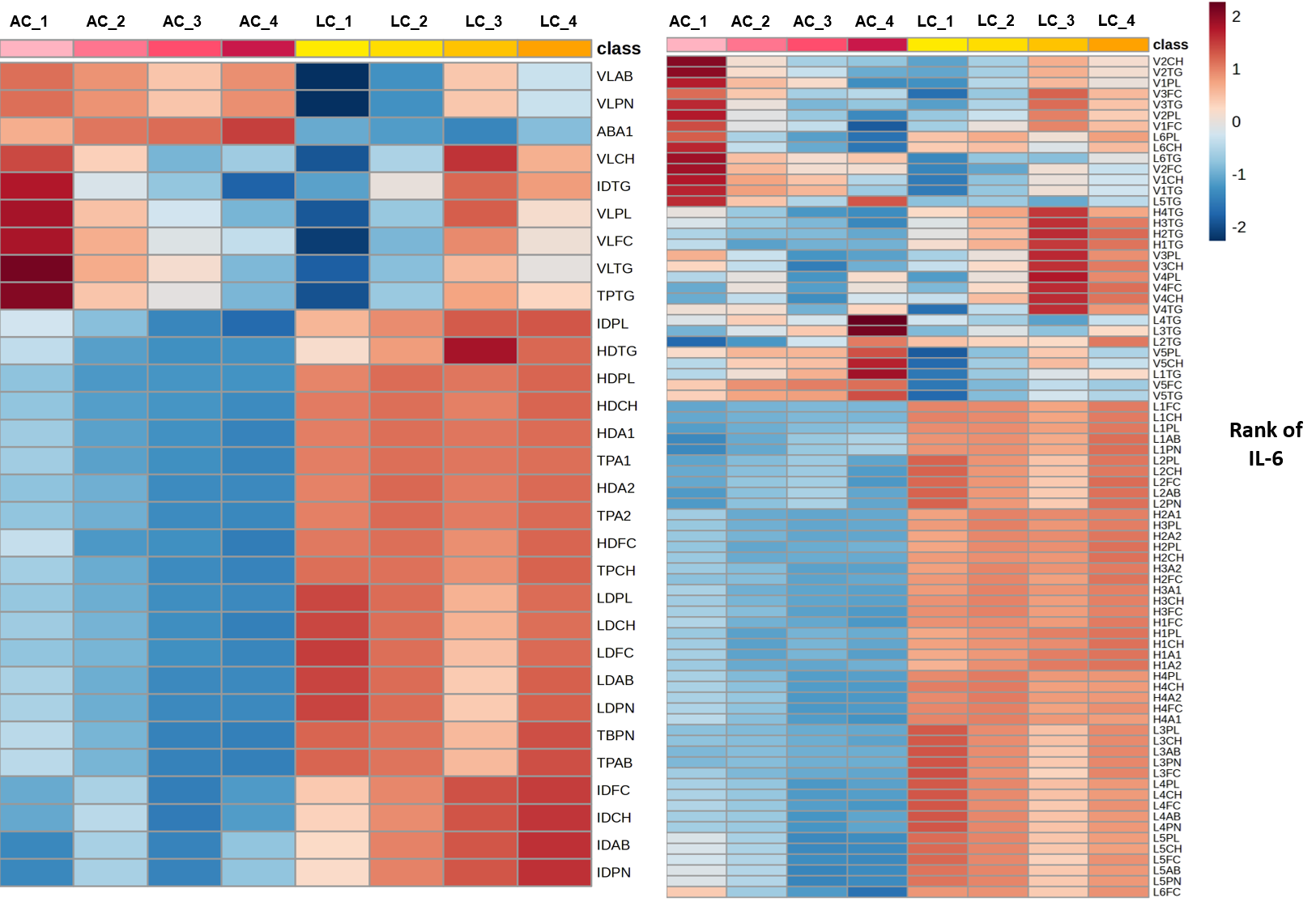


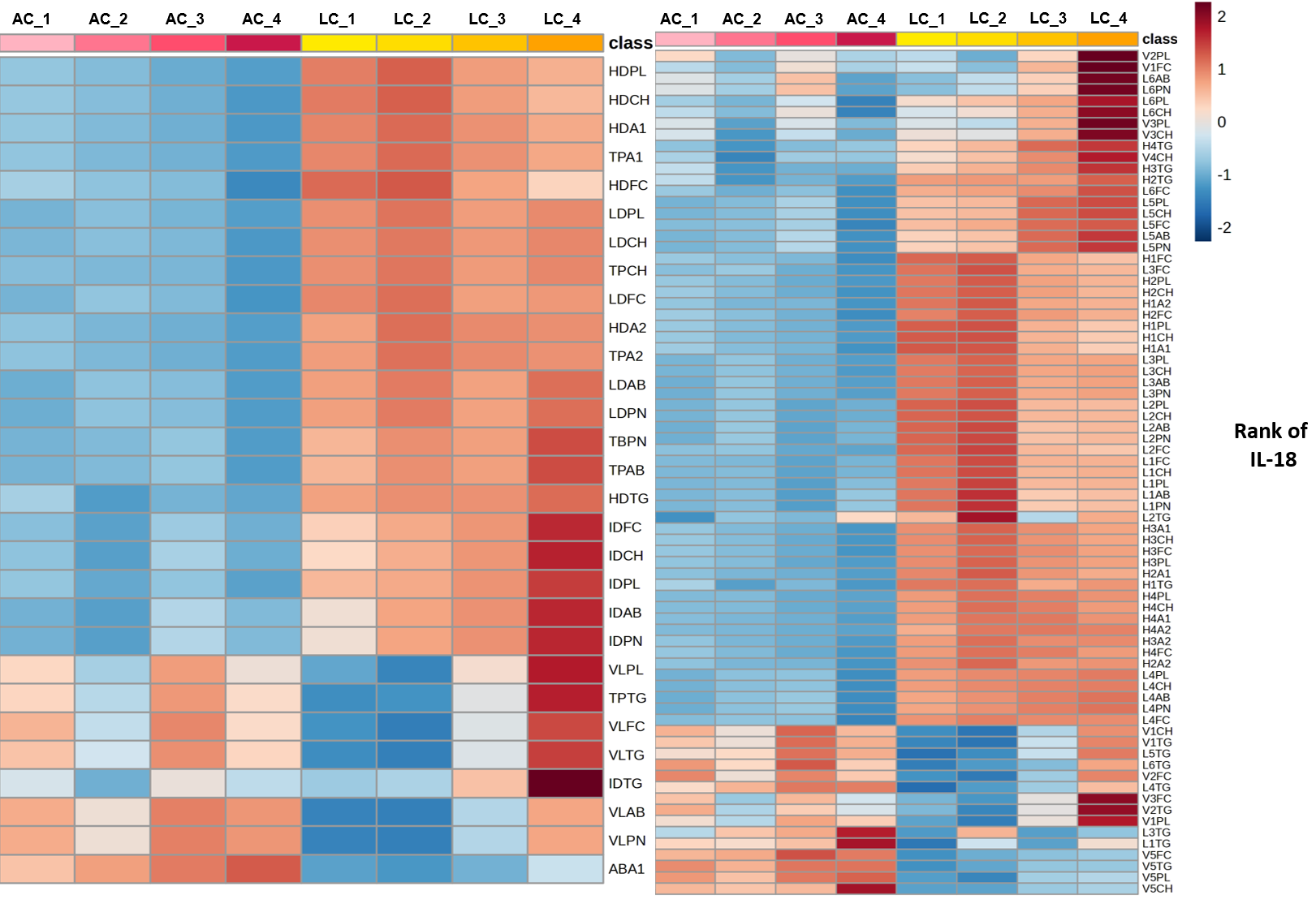


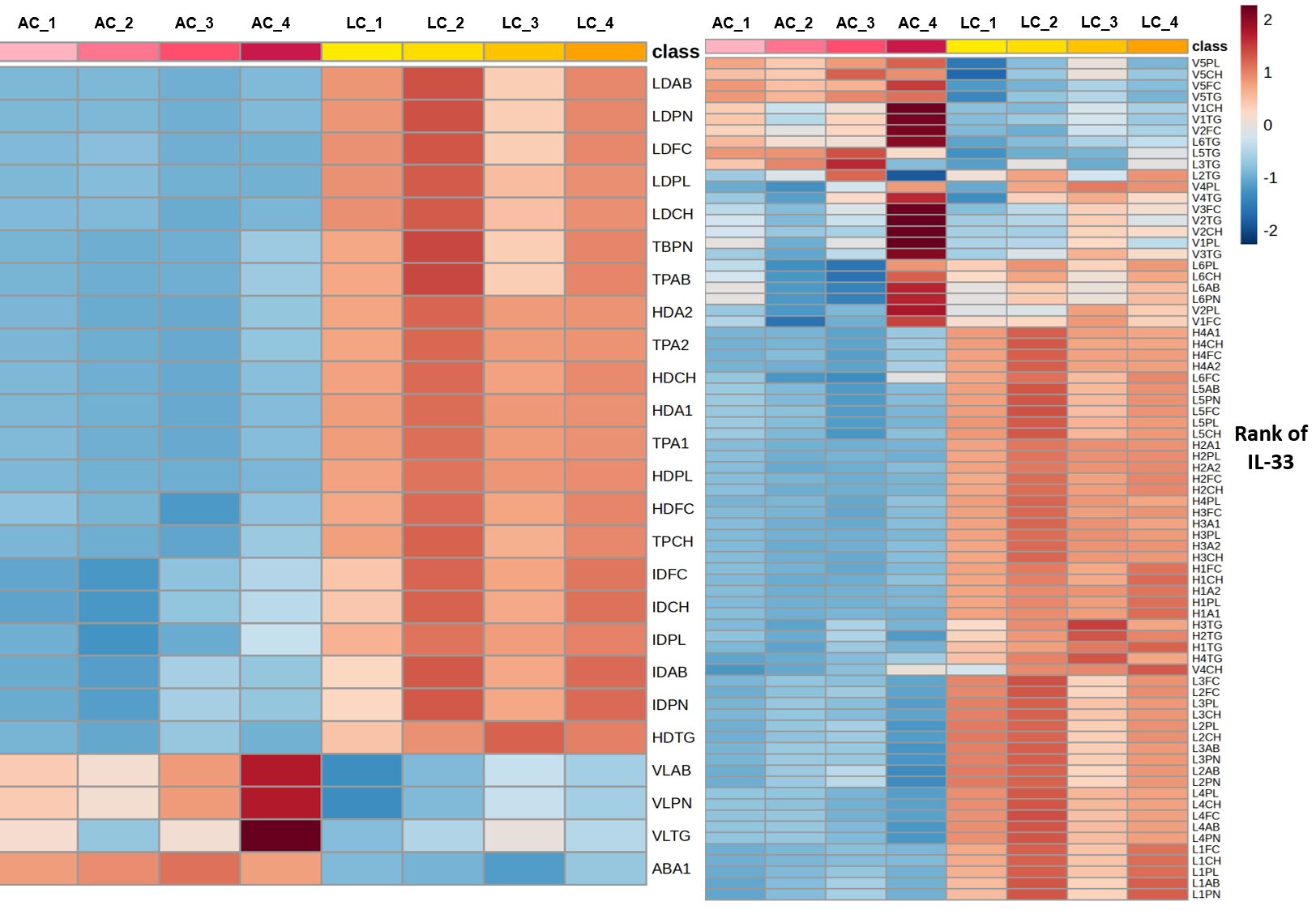


**Supplementary figure 8 – Re-stratified of acute and long COVID-19 cohorts based on the rank of MCP-1, IL-6, IL-18, and IL-33.**

Heatmaps shows significantly altered main and subfraction of NMR lipoprotein (adjusted p-value < 0.05 by Ordinary ANOVA, Kruskal Wallis, and Brown Forsythe & Welch ANOVA tests). The average concentration of each lipoprotein parameter is displayed based on the color scale.

**
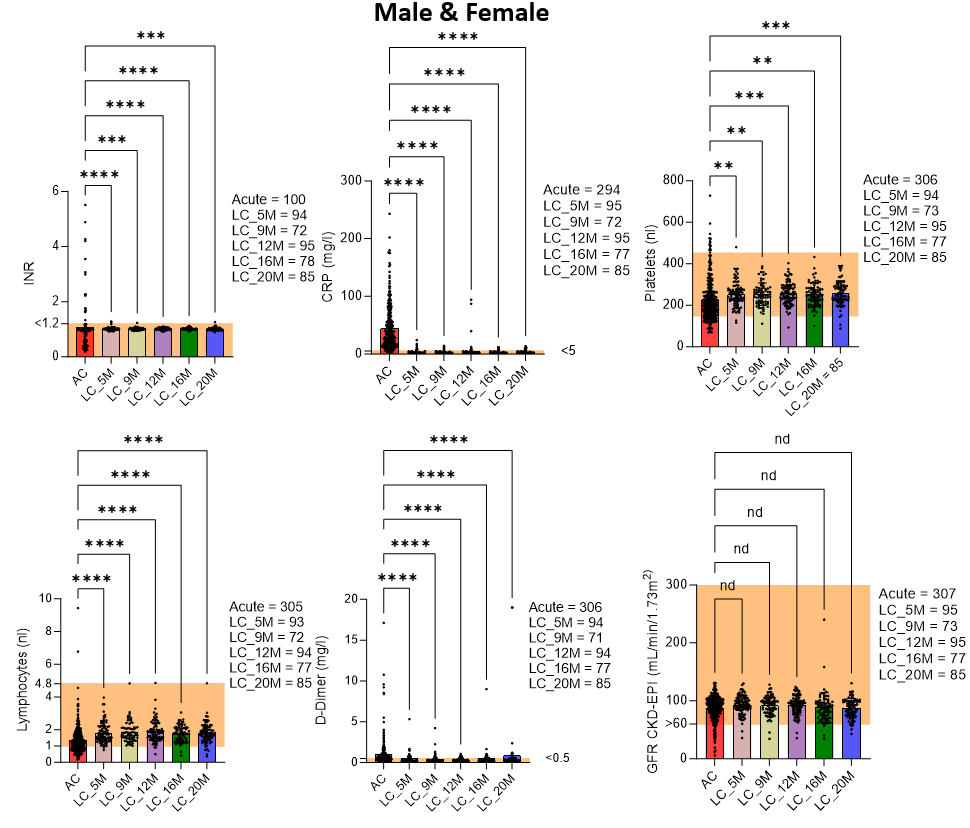
**


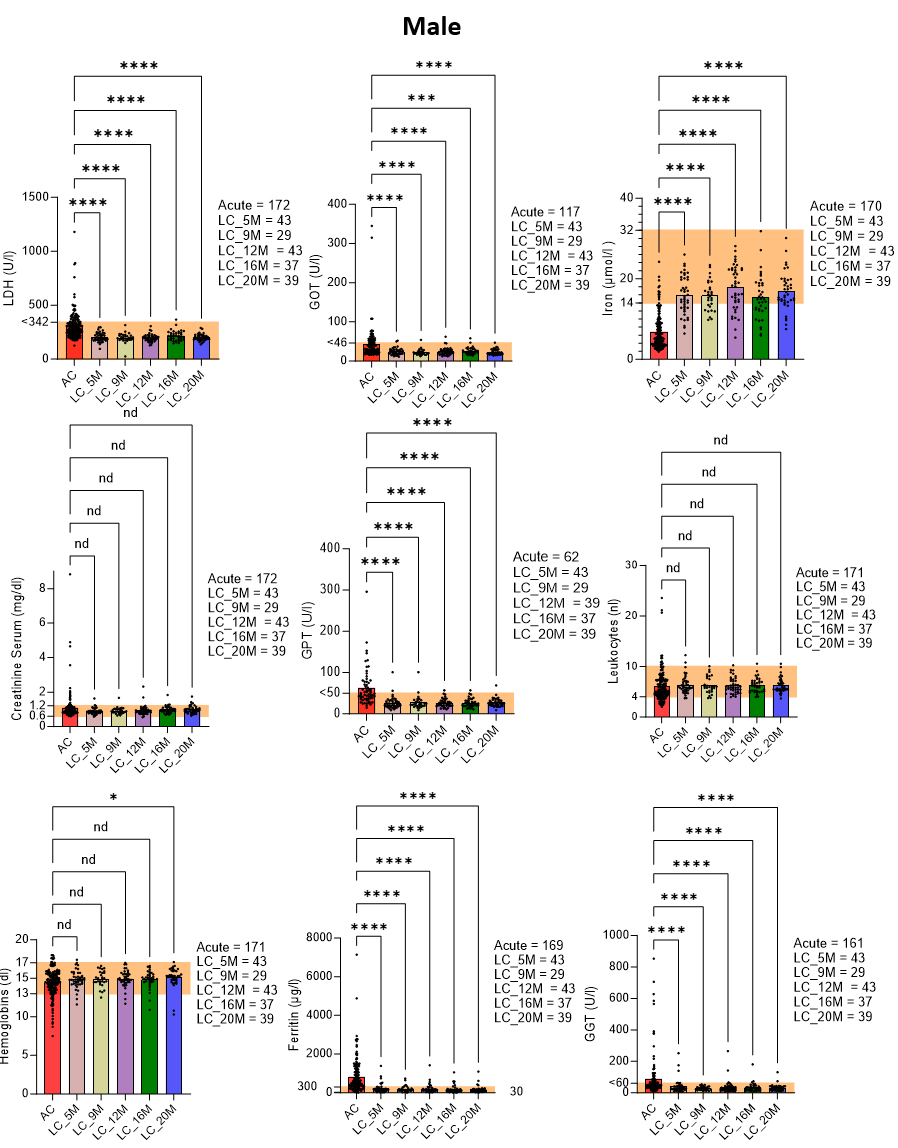


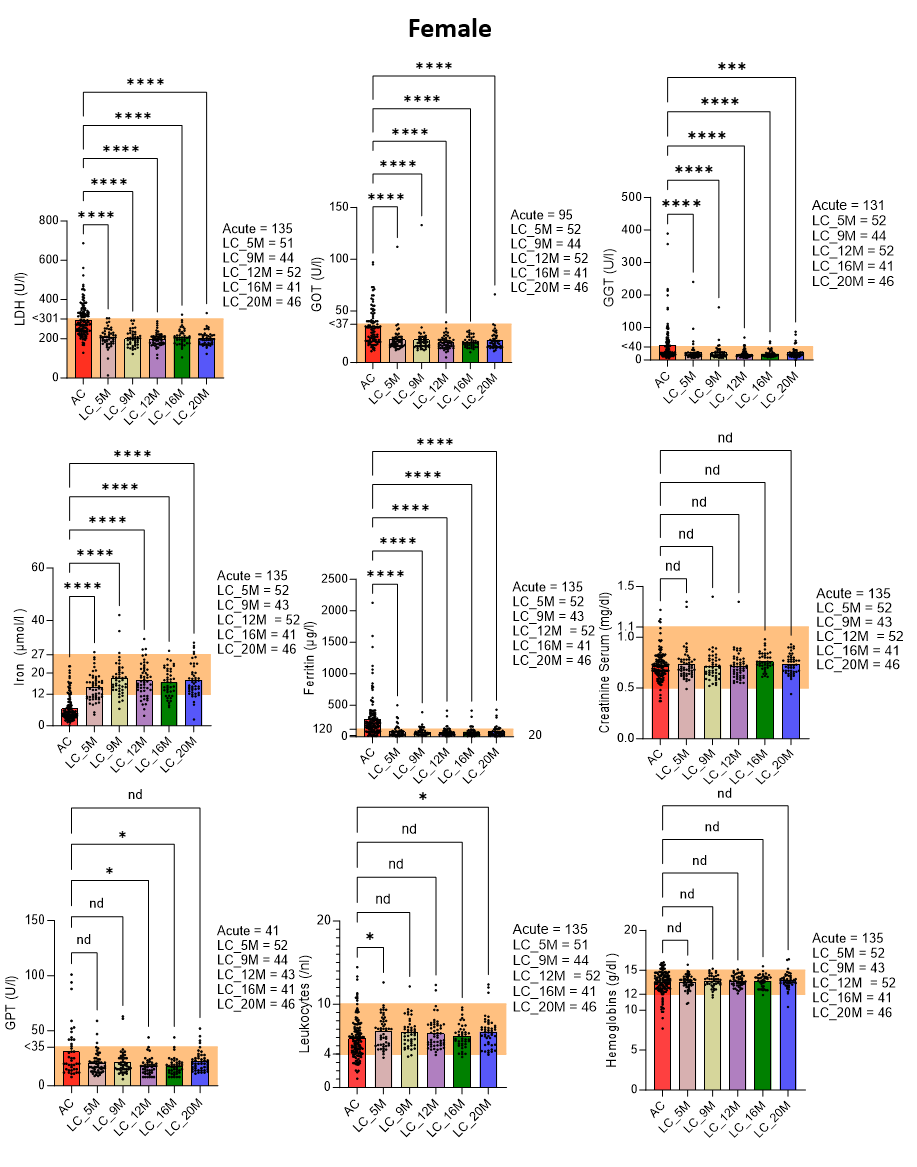


**Supplementary figure 9 – Clinical lab parameters of acute and long COVID-19 cohorts.**

INR, CRP, platelets, lymphocytes, D-dimer, LDH, GOT, iron, GPT, hemoglobin, ferritin, and GGT were significantly different between acute and long COVID-19 cohorts (adjusted p-value < 0.05 by Ordinary ANOVA, Kruskal Wallis, and Brown Forsythe & Welch ANOVA tests and post hoc test of the statistics: q-value = nd > 0.05, *<0.05, **< 0.01, ***<0.001, ****<0.0001). Each shaded line represents the baseline of the clinical lab parameter.


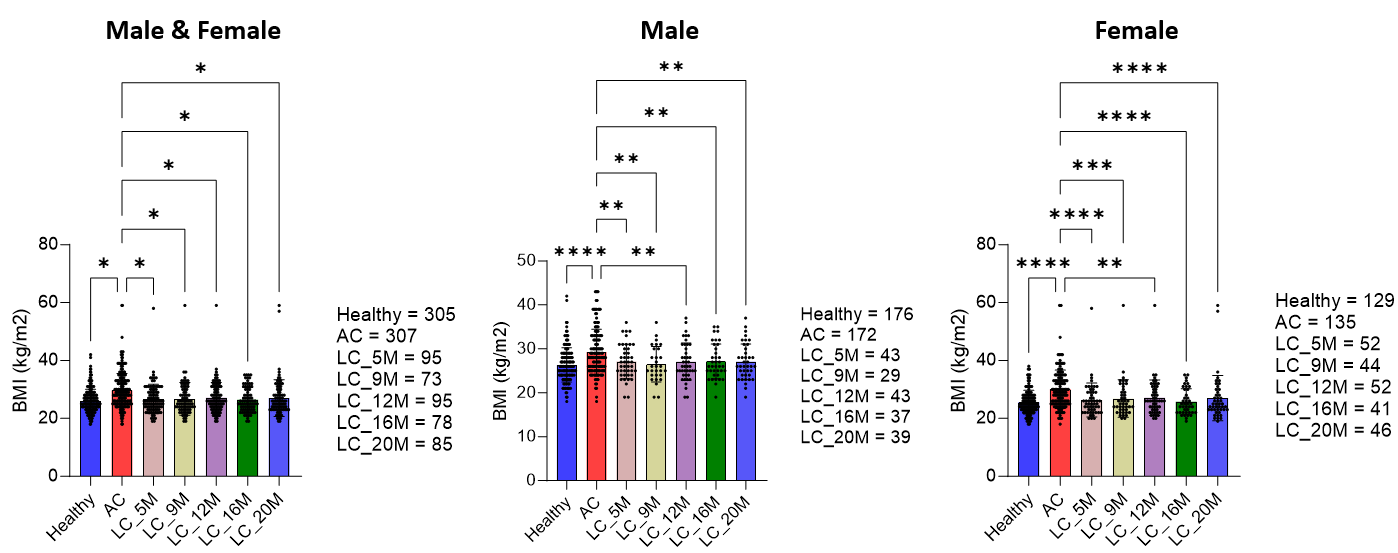


**Supplementary figure 10 – BMI of healthy, acute, and long COVID-19 cohorts based on sex.**

BMI was significantly different between acute and long COVID-19 cohorts (adjusted p-value < 0.05 by Ordinary ANOVA and Kruskal Wallis tests and post hoc test of the statistics: q-value = *<0.05, **< 0.01, ***<0.001, ****<0.0001). Acute COVID-19 cohort exhibited significantly higher BMI compared to healthy and long COVID-19 cohorts. Moreover, BMI remain unaffected in the comparison between healthy and long COVID-19 cohorts.


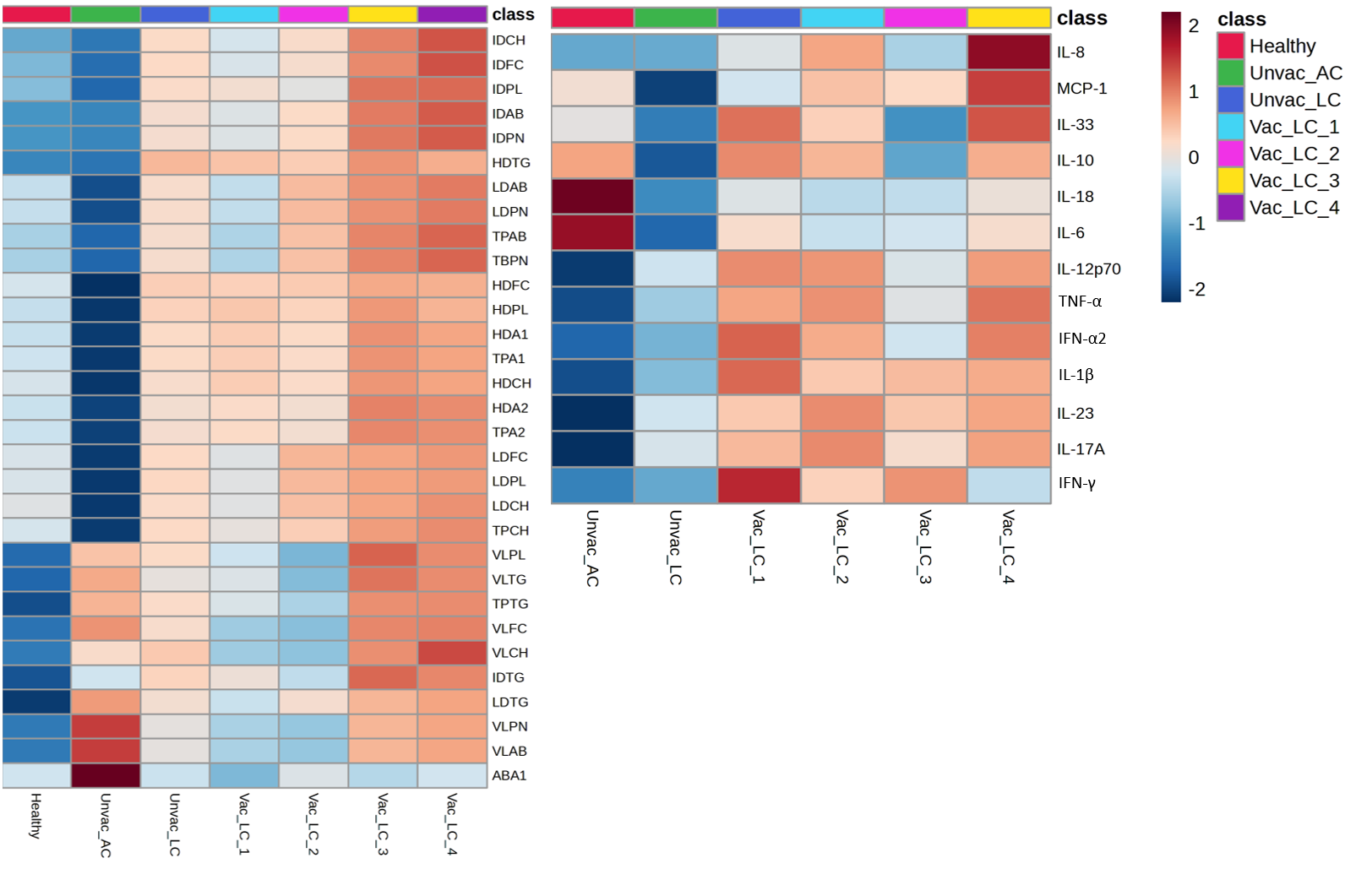


**Supplementary figure 11 – Vaccination effect on lipoprotein metabolism, pro, and anti-inflammatory mediators.**

Heatmaps shows 32 significant main lipoproteins and 13 cytokines parameters (adjusted p-value < 0.05 by Ordinary ANOVA, Kruskal Wallis, and Brown Forsythe & Welch ANOVA tests). The average concentration of each lipoprotein parameter is displayed based on the color scale.


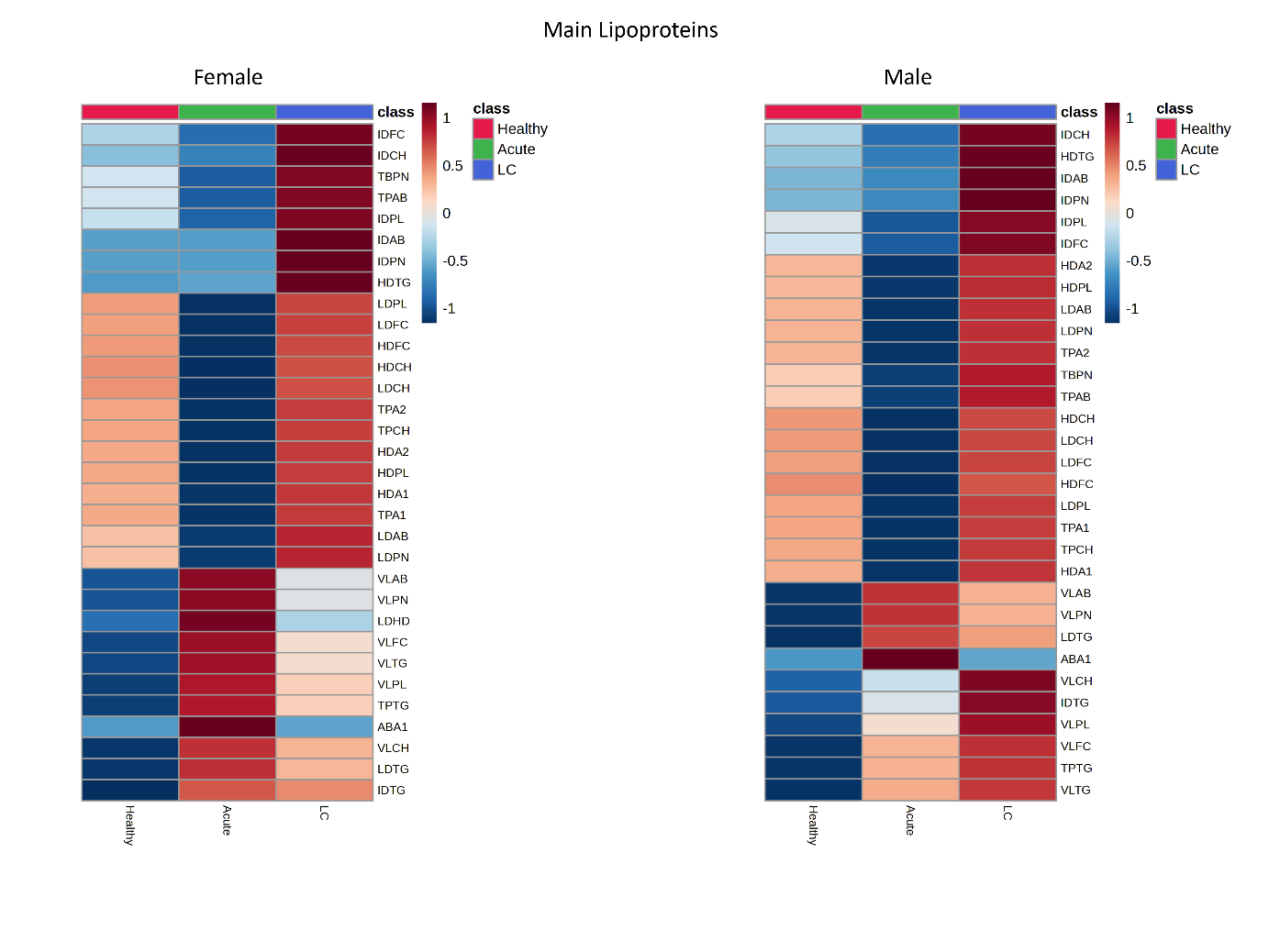

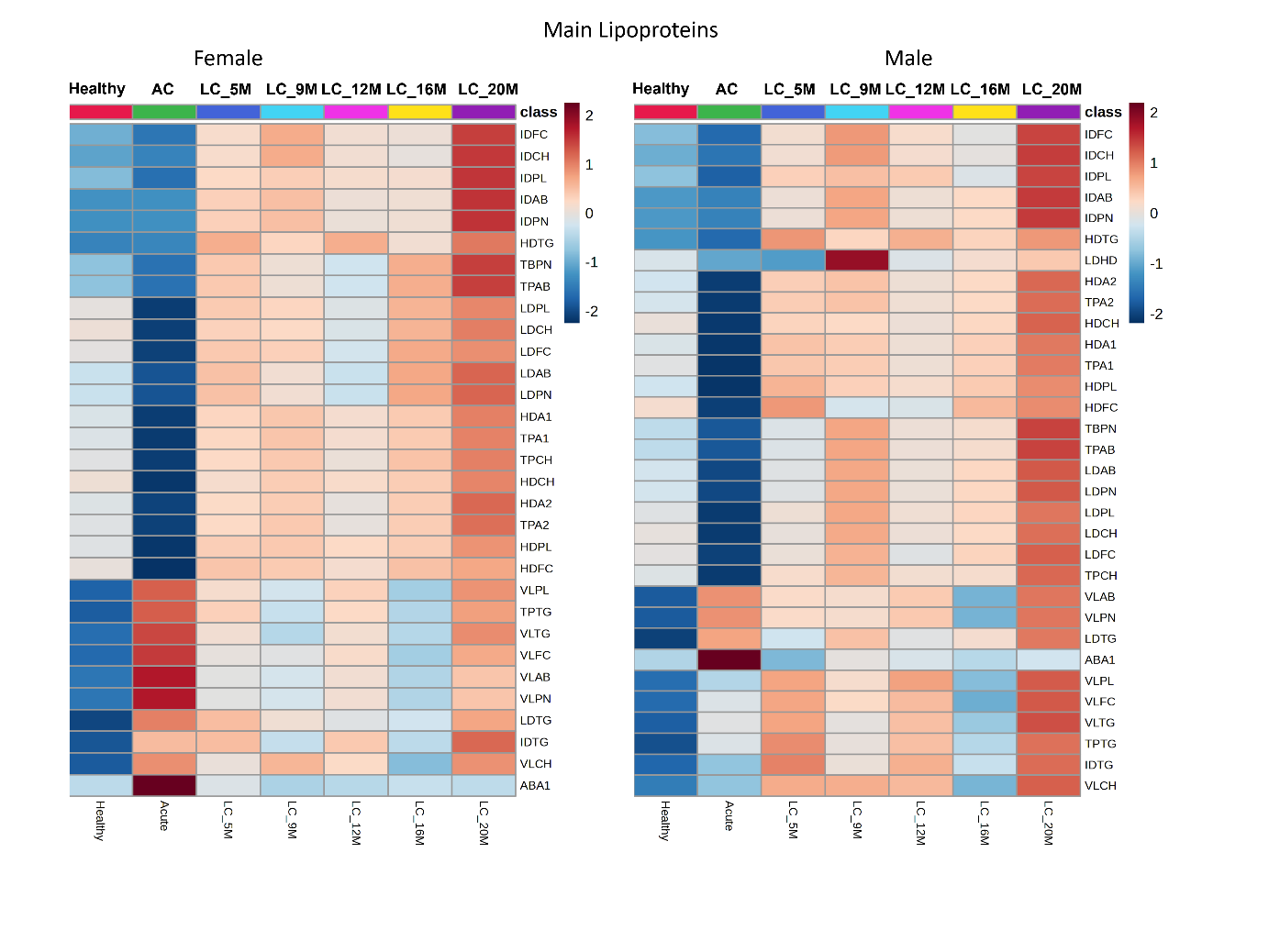

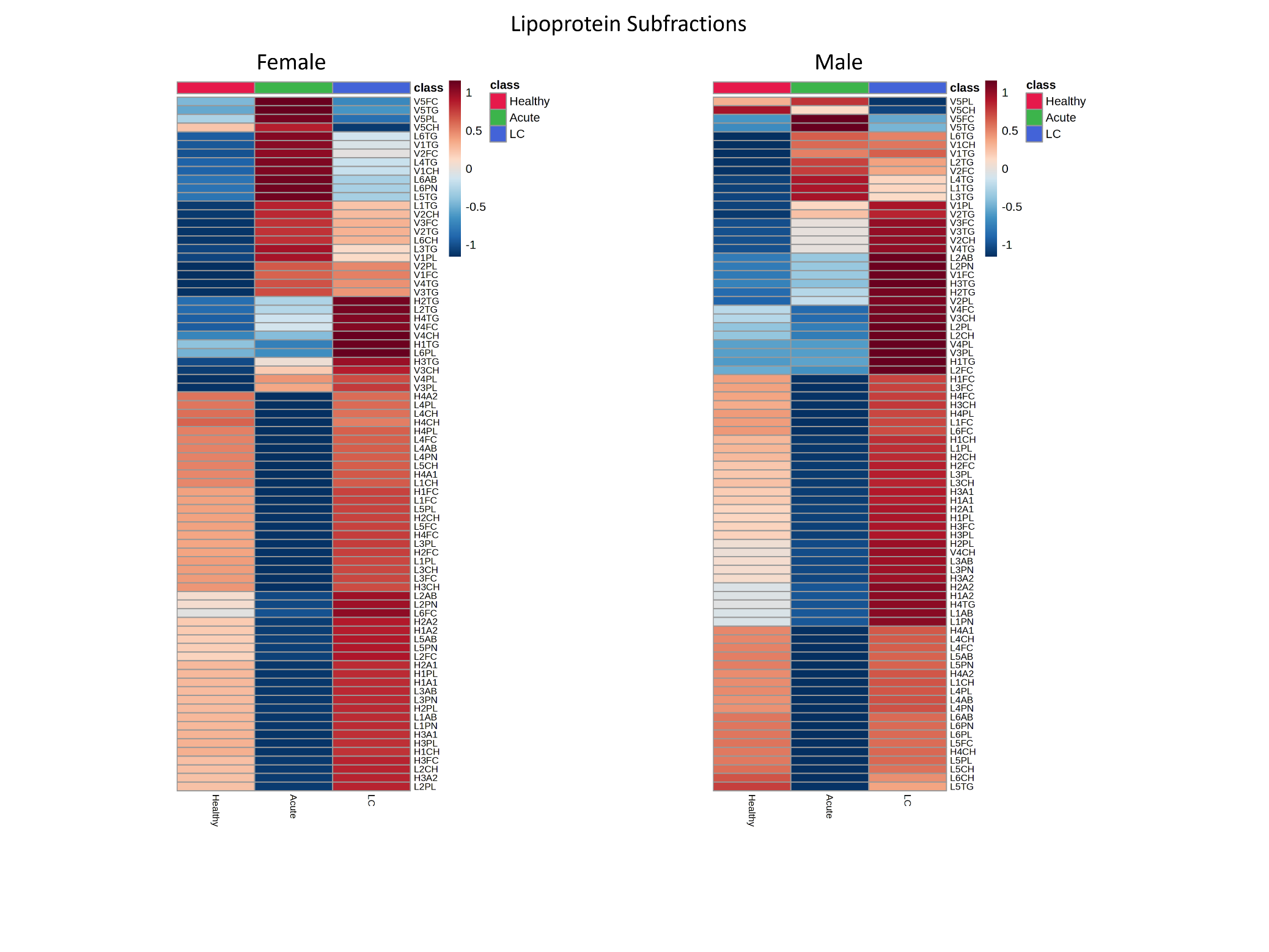

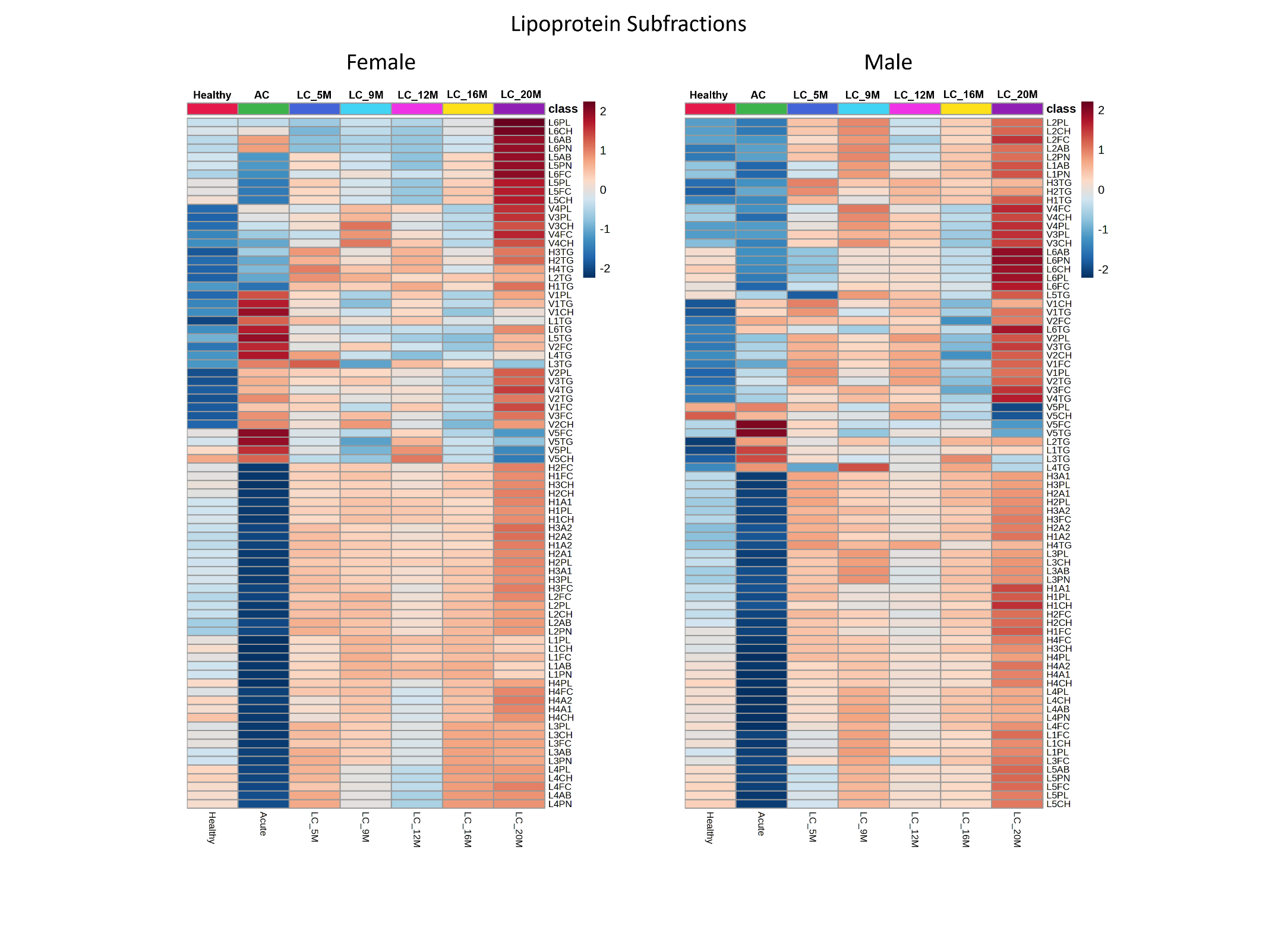

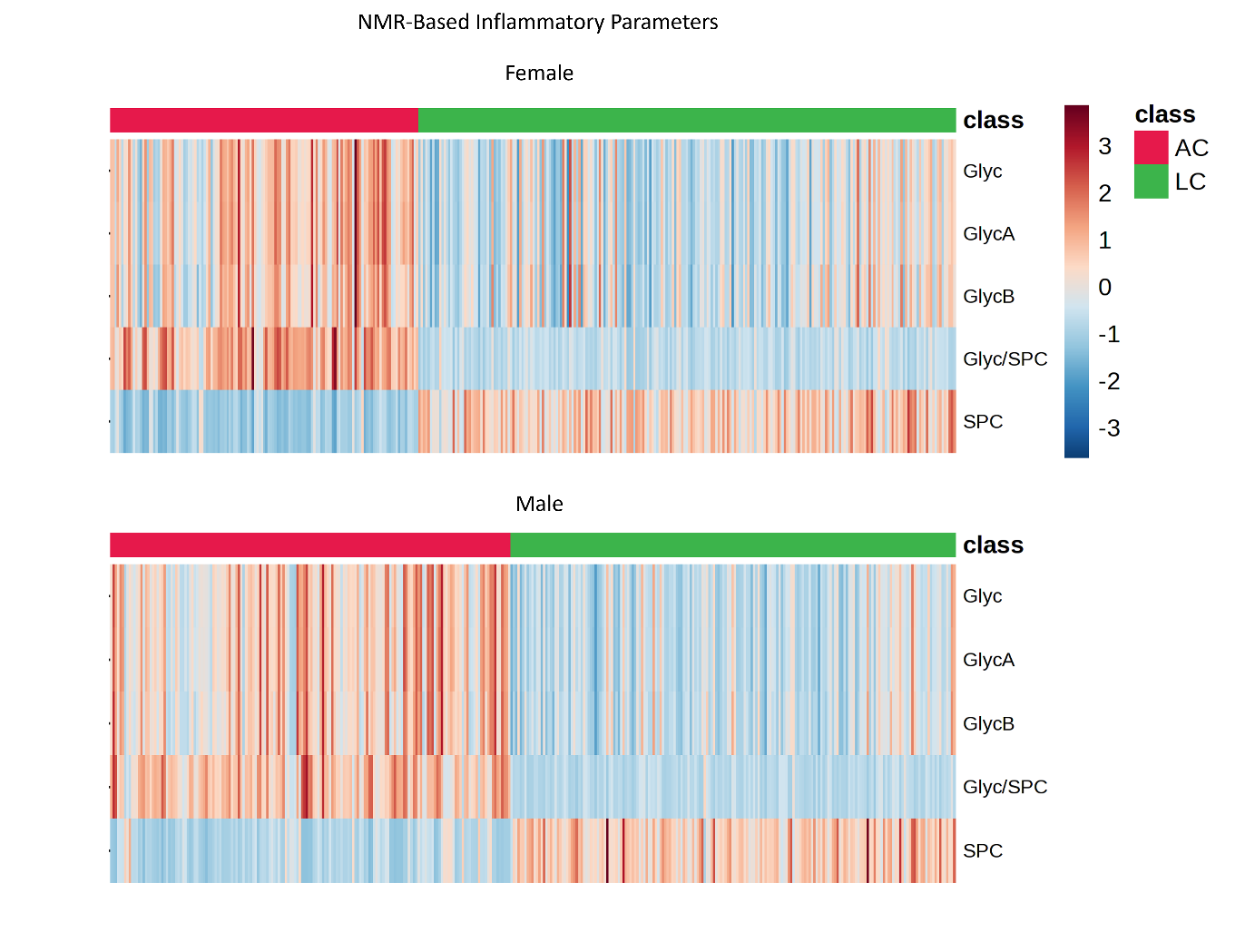

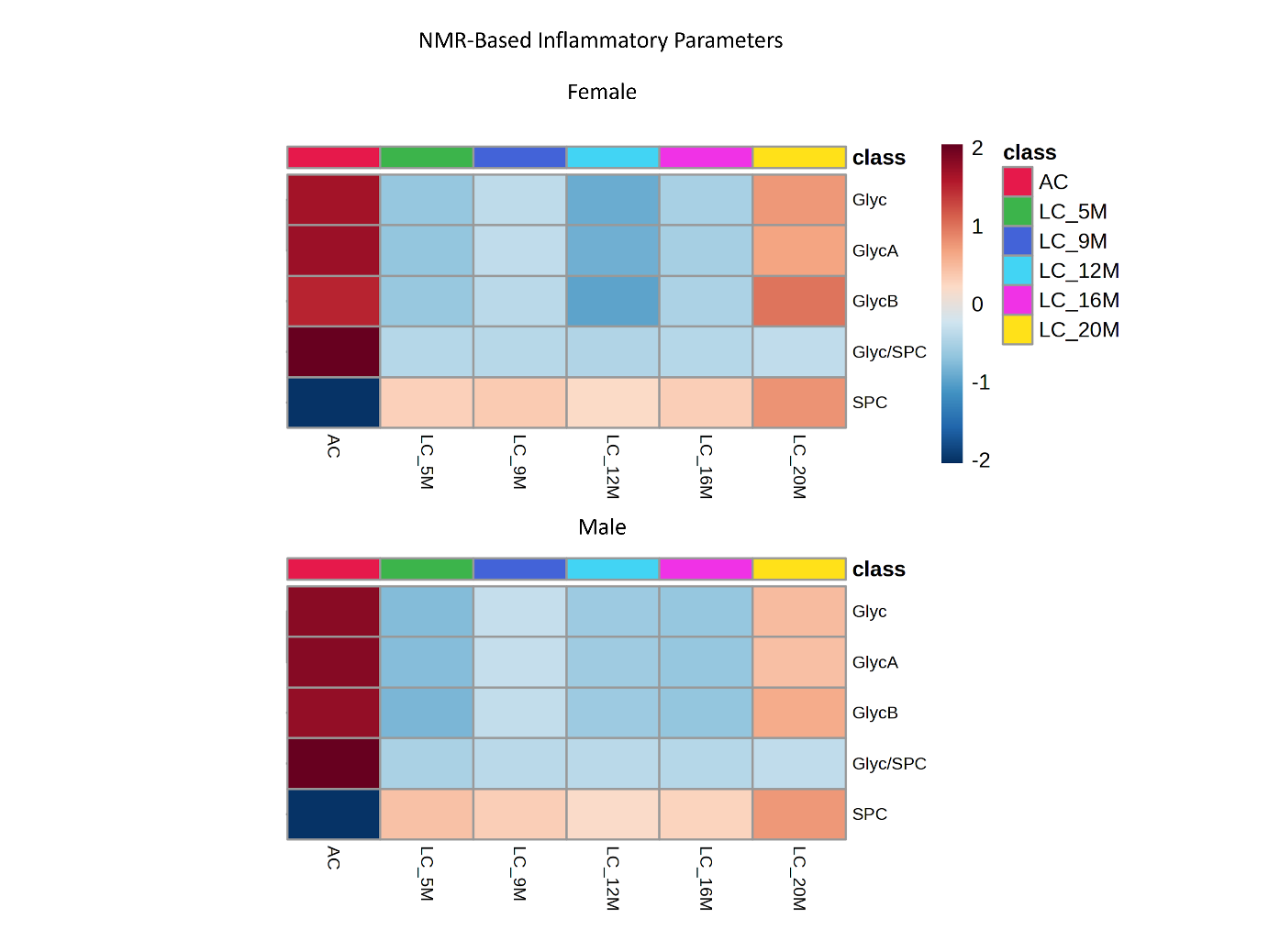

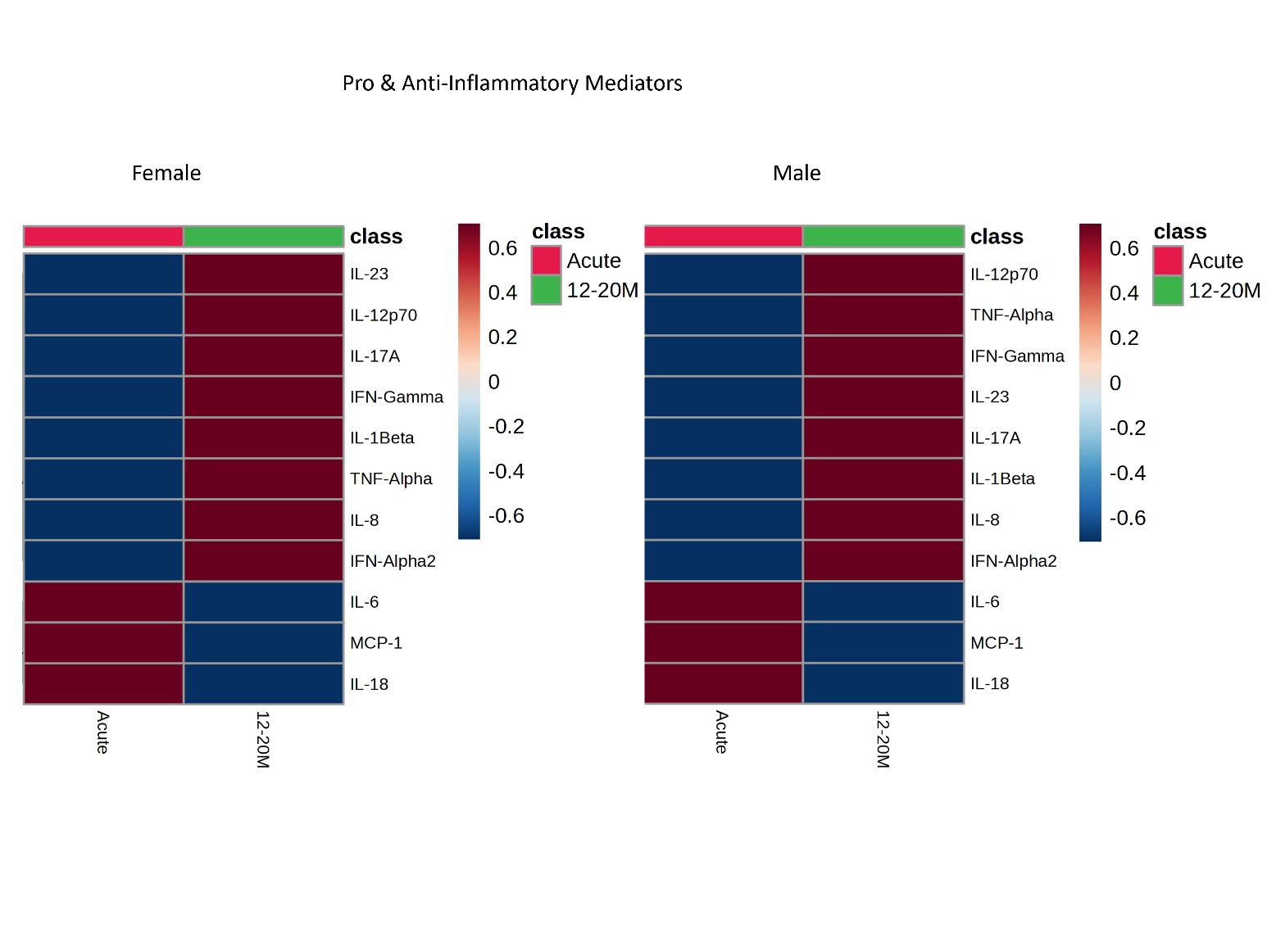

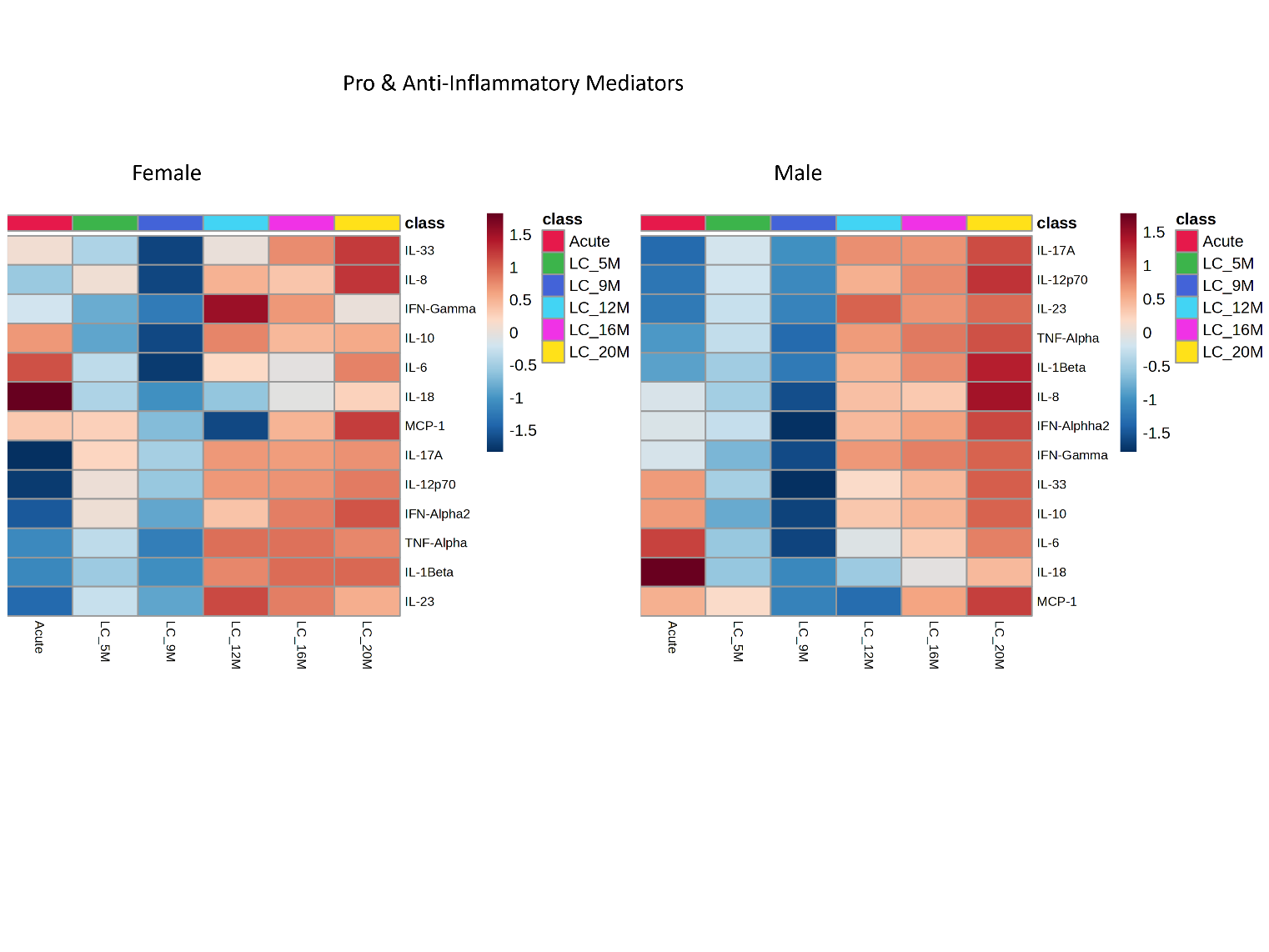


**Supplementary Figure 12 – Sex independent hyperlipidemic phenotype with chronic inflammation in Long COVID-19.**

The average concentration of each lipoprotein and pro & anti-inflammatory parameters is displayed based on the color scale. Profiles of these parameters were almost comparable between male and female Long COVID-19 cohorts.

**References**

Alspach, E., Lussier, D.M. and Schreiber, R.D. (2019) Interferon γ and Its Important Roles in Promoting and Inhibiting Spontaneous and Therapeutic Cancer Immunity. *Cold Spring Harb Perspect Biol* **11**.

Anquetil, F., Sabouri, S., Thivolet, C., Rodriguez-Calvo, T., Zapardiel-Gonzalo, J., Amirian, N., Schneider, D., Castillo, E., Lajevardi, Y. and von Herrath, M.G. (2017) Alpha cells, the main source of IL-1β in human pancreas. *J Autoimmun* **81,** 68-73.

Chen, J., Liao, M.Y., Gao, X.L., Zhong, Q., Tang, T.T., Yu, X., Liao, Y.H. and Cheng, X. (2013) IL-17A induces pro-inflammatory cytokines production in macrophages via MAPKinases, NF-κB and AP-1. *Cell Physiol Biochem* **32,** 1265-74.

Corre, I., Pineau, D. and Hermouet, S. (1999) Interleukin-8: an autocrine/paracrine growth factor for human hematopoietic progenitors acting in synergy with colony stimulating factor-1 to promote monocyte-macrophage growth and differentiation. *Exp Hematol* **27,** 28-36.

D'Andrea, A., Rengaraju, M., Valiante, N.M., Chehimi, J., Kubin, M., Aste, M., Chan, S.H., Kobayashi, M., Young, D., Nickbarg, E. and et al. (1992) Production of natural killer cell stimulatory factor (interleukin 12) by peripheral blood mononuclear cells. *J Exp Med* **176,** 1387-98.

David, J.M., Dominguez, C., Hamilton, D.H. and Palena, C. (2016) The IL-8/IL-8R Axis: A Double Agent in Tumor Immune Resistance. *Vaccines (Basel)* **4**.

de Lemos, J.A., Morrow, D.A., Sabatine, M.S., Murphy, S.A., Gibson, C.M., Antman, E.M., McCabe, C.H., Cannon, C.P. and Braunwald, E. (2003) Association between plasma levels of monocyte chemoattractant protein-1 and long-term clinical outcomes in patients with acute coronary syndromes. *Circulation* **107,** 690-5.

Deshmane, S.L., Kremlev, S., Amini, S. and Sawaya, B.E. (2009) Monocyte chemoattractant protein-1 (MCP-1): an overview. *J Interferon Cytokine Res* **29,** 313-26.

Feingold, K.R. (2022) Lipid and Lipoprotein Metabolism. *Endocrinol Metab Clin North Am* **51,** 437-458.

Florea, G., Tudorache, I.F., Fuior, E.V., Ionita, R., Dumitrescu, M., Fenyo, I.M., Bivol, V.G. and Gafencu, A.V. (2022) Apolipoprotein A-II, a Player in Multiple Processes and Diseases. *Biomedicines* **10**.

Fock, V., Mairhofer, M., Otti, G.R., Hiden, U., Spittler, A., Zeisler, H., Fiala, C., Knöfler, M. and Pollheimer, J. (2013) Macrophage-derived IL-33 is a critical factor for placental growth. *J Immunol* **191,** 3734-43.

Gabay, C. (2006) Interleukin-6 and chronic inflammation. *Arthritis Res Ther* **8 Suppl 2,** S3.

Gaffen, S.L. (2009) Structure and signalling in the IL-17 receptor family. *Nat Rev Immunol* **9,** 556-67.

Guo, J., Chen, S., Zhang, Y., Liu, J., Jiang, L., Hu, L., Yao, K., Yu, Y. and Chen, X. (2024) Cholesterol metabolism: physiological regulation and diseases. *MedComm (2020)* **5,** e476.

Horiuchi, T., Mitoma, H., Harashima, S., Tsukamoto, H. and Shimoda, T. (2010) Transmembrane TNF-alpha: structure, function and interaction with anti-TNF agents. *Rheumatology (Oxford)* **49,** 1215-28.

Hsu, C.L., Neilsen, C.V. and Bryce, P.J. (2010) IL-33 is produced by mast cells and regulates IgE-dependent inflammation. *PLoS One* **5,** e11944.

Idriss, H.T. and Naismith, J.H. (2000) TNF alpha and the TNF receptor superfamily: structure-function relationship(s). *Microsc Res Tech* **50,** 184-95.

Ihim, S.A., Abubakar, S.D., Zian, Z., Sasaki, T., Saffarioun, M., Maleknia, S. and Azizi, G. (2022) Interleukin-18 cytokine in immunity, inflammation, and autoimmunity: Biological role in induction, regulation, and treatment. *Front Immunol* **13,** 919973.

Iyer, S.S. and Cheng, G. (2012) Role of interleukin 10 transcriptional regulation in inflammation and autoimmune disease. *Crit Rev Immunol* **32,** 23-63.

Kamimura, D., Ishihara, K. and Hirano, T. (2003) IL-6 signal transduction and its physiological roles: the signal orchestration model. *Rev Physiol Biochem Pharmacol* **149,** 1-38.

Korn, T. and Hiltensperger, M. (2021) Role of IL-6 in the commitment of T cell subsets. *Cytokine* **146,** 155654.

Liu, J., Cao, S., Kim, S., Chung, E.Y., Homma, Y., Guan, X., Jimenez, V. and Ma, X. (2005) Interleukin-12: an update on its immunological activities, signaling and regulation of gene expression. *Curr Immunol Rev* **1,** 119-137.

Lopez-Castejon, G. and Brough, D. (2011) Understanding the mechanism of IL-1β secretion. *Cytokine Growth Factor Rev* **22,** 189-95.

Macatonia, S.E., Hosken, N.A., Litton, M., Vieira, P., Hsieh, C.S., Culpepper, J.A., Wysocka, M., Trinchieri, G., Murphy, K.M. and O'Garra, A. (1995) Dendritic cells produce IL-12 and direct the development of Th1 cells from naive CD4+ T cells. *J Immunol* **154,** 5071-9.

Masuda, R., Wist, J., Lodge, S., Kimhofer, T., Hunter, M., Hui, J., Beilby, J.P., Burnett, J.R., Dwivedi, G., Schlaich, M.P., Bong, S.H., Loo, R.L., Holmes, E., Nicholson, J.K. and Yeap, B.B. (2023) Plasma lipoprotein subclass variation in middle-aged and older adults: Sex-stratified distributions and associations with health status and cardiometabolic risk factors. *J Clin Lipidol* **17,** 677-687.

McKenzie, B.S., Kastelein, R.A. and Cua, D.J. (2006) Understanding the IL-23-IL-17 immune pathway. *Trends Immunol* **27,** 17-23.

McNab, F., Mayer-Barber, K., Sher, A., Wack, A. and O'Garra, A. (2015) Type I interferons in infectious disease. *Nat Rev Immunol* **15,** 87-103.

Prchal, M., Pilz, A., Simma, O., Lingnau, K., von Gabain, A., Strobl, B., Müller, M. and Decker, T. (2009) Type I interferons as mediators of immune adjuvants for T- and B cell-dependent acquired immunity. *Vaccine* **27 Suppl 6,** G17-20.

Rader, D.J., Castro, G., Zech, L.A., Fruchart, J.C. and Brewer, H.B., Jr. (1991) In vivo metabolism of apolipoprotein A-I on high density lipoprotein particles LpA-I and LpA-I,A-II. *J Lipid Res* **32,** 1849-59.

Schmitz, J., Owyang, A., Oldham, E., Song, Y., Murphy, E., McClanahan, T.K., Zurawski, G., Moshrefi, M., Qin, J., Li, X., Gorman, D.M., Bazan, J.F. and Kastelein, R.A. (2005) IL-33, an interleukin-1-like cytokine that signals via the IL-1 receptor-related protein ST2 and induces T helper type 2-associated cytokines. *Immunity* **23,** 479-90.

Singh, S., Anshita, D. and Ravichandiran, V. (2021) MCP-1: Function, regulation, and involvement in disease. *Int Immunopharmacol* **101,** 107598.

Swain, S.L. (2001) Interleukin 18: tipping the balance towards a T helper cell 1 response. *J Exp Med* **194,** F11-4.

Tang, C., Chen, S., Qian, H. and Huang, W. (2012) Interleukin-23: as a drug target for autoimmune inflammatory diseases. *Immunology* **135,** 112-24.

Watt, M.J. and Cheng, Y. (2017) Triglyceride metabolism in exercising muscle. *Biochim Biophys Acta Mol Cell Biol Lipids* **1862,** 1250-1259.

Yi, X.M., Lian, H. and Li, S. (2022) Signaling and functions of interleukin-33 in immune regulation and diseases. *Cell Insight* **1,** 100042.
